# Supplementary material for: Colonizing multidrug-resistant bacteria and the longitudinal evolution of the intestinal microbiome after liver transplantation
Source: Nat Commun. 2019 Oct 17;10:4715. doi: 10.1038/s41467-019-12633-4 (PMC6797753; doi:10.1038/s41467-019-12633-4)
Supplement: Supplementary file 1 — Supplementary Information [file 41467_2019_12633_MOESM1_ESM.pdf]

Colonizing multidrug-resistant bacteria and the longitudinal evolution of the intestinal microbiome after liver transplantation

Annavajhala et al.

Supplementary Information

Supplementary Methods

Stool sample collection and processing

Inpatient study subjects were asked to provide stool samples which were collected by nursing staff, stored at 4°C, and transported to the laboratory for processing. Outpatients were provided collection kits to sample stool ≤ 24 hours before their next clinic appointment, store at 4°C, and return to the clinic on ice packs. Aliquoted fecal samples were stored at -80°C.

Clinical and sample metadata collection and coding

Donor characteristics: Donor type (living vs. deceased) was coded as a binary variable based on data obtained from the United Network for Organ Sharing (UNOS).

Patient demographics: Patient sex and age at transplant were obtained through chart review. Race and ethnicity data was obtained from UNOS.

Liver disease etiology and severity: Primary liver etiology was determined through careful chart review to determine primary underlying cause for transplantation. For patients with multiple or unclear diagnoses, pathology review was performed by a liver transplant specialist (ECV) prior to coding as one of the above categories. Resulting categories were, in order of incidence in the cohort, (1) hepatitis C virus (HCV), (2) non-alcoholic fatty liver disease (NAFLD), (3) alcohol (ARLD), (4) biliary-related etiologies (BILIARY; see below), (5) autoimmune hepatitis (AIH), (6) hepatitis B virus (HBV), (7) polycystic liver/kidney disease (PCLD), (8) cryptogenic liver disease (CLD), and (9) other. Several etiologies related to biliary complications were grouped together (BILIARY), including primary and secondary sclerosing cholangitis (PSC, SSC), primary biliary cholangitis (PBC), biliary cirrhosis (BC), recurrent cholangitis (RC), and biliary atresia (BA). Alpha-1 antitrypsin deficiency and concurrent hepatocellular carcinoma (HCC) were coded separately as binary variables.

Markers for disease severity included transplant model for end-stage liver disease (MELD) and Child-Turcotte-Pugh (CTP) scores. MELD scores were calculated for each patient according to the Organ Procurement and Transplantation Network (OPTN) guidelines, which were updated in January 2016 to account for serum sodium levels.(1) MELD was included as a continuous variable and as a categorical variable coded as above vs. below or equal to the median value in the cohort (18). CTP scores were calculated according to OPTN guidelines, and were coded as a continuous variable and by commonly applied categories (A: CTP of 5-6, B: 7-9, C: 10-15).

Transplant-related mechanical parameters and complications: Cold, warm, and total ischemia times were obtained from chart review. The units of packed red blood cells (PRBC) transfused during the transplant surgery were also included as a continuous variable. Post-operative bile leak, biliary stricture, and bleeding were coded as binary variables after chart review as described previously.(2) Bleeding was defined as post-operative bleeding occurring within 7 days post-transplant, as evidenced by drop in hemoglobin/hematocrit or hemorrhagic abdominal drainage. Diagnosis of biliary complications was made on the basis of compatible clinical features and by findings on imaging (ultrasonography and/or magnetic resonance cholangiopancreatography (MRCP), end-retroscopic cholangiopancreatography (ERCP) or percutaneous transhepatic cholangiography (PTC) in conjunction with clinical documentation by transplant physicians. Biliary strictures included anastomotic and non-anastomotic biliary strictures. Bile leaks included early and late bile leaks.

Post-transplant long-term outcomes: All-cause death, readmission to the hospital, and readmission to the intensive care unit (ICU) within one year post-transplant were obtained through chart review and coded as binary variables. Only six patients (3%) died within one-year post-transplant, precluding the inclusion of mortality in our statistical models.

Antibiotic usage: Peri-operative antibiotic use was coded as a binary variable defined by use of a standard versus alternative regimen, where the standard regimen was consistent with hospital-

based guidelines and included 3 grams ampicillin/sulbactam administered every 2 hours for creatinine clearance ≥30 mL/min and every 6 hours for creatinine clearance <30 mL/min for the duration of the surgical procedure. Alternative regimens were determined prior to surgery in conjunction with consulting infectious diseases physicians based a documented allergy to the standard regimen or prior or concomitant colonization or infection with bacteria known to be non-susceptible to the standard regimen. In addition, all inpatient antibiotic use during the 6 months prior to and one year after transplant was extracted from medical records, including the date and time of administration and dosage. Outpatient antibiotic usage data was incomplete and thus excluded from our analyses. Antibacterial agents were grouped by class (aminoglycoside, beta-lactam, glyco/lipopeptide, quinolone, tetracycline, and other). Beta-lactam antibiotics were further subdivided into subclasses based on generation and spectrum (group 1: penicillins and 1st/2nd generation cephalosporins; group 2: 3rd generation cephalosporins, monobactams, first-generation beta-lactamase inhibitor combinations (including piperacillin-tazobactam), and ceftaroline; group 3: 4th generation cephalosporins and novel beta-lactamase inhibitor combinations).

Stool samples: We calculated the days pre- or post-transplant for each stool sample based on sample collection date and date of transplant. The samples were also categorized into the following time categories: pre-transplant, week 1, 2, or 3 post-transplant, and months 1, 2, 3, 6, 9, or 12 post-transplant. Additionally, peri-transplant (weeks 1-3) and post-transplant (months 1-12) samples were grouped together for some analyses. Samples were also coded as collected within 7, 14, or 30 days of (a) rejection episode or (b) antibiotic usage.

Multidrug-resistant bacteria (MDRB) colonization: We isolated carbapenem-resistant Enterobacteriaceae (CRE), third-generation cephalosporin-resistant Enterobacteriaceae (Ceph-RE), and/or vancomycin-resistant enterococci (VRE) from stool samples using selective chromogenic agar (DRG International). The isolation of CRE, Ceph-RE, or VRE from a given stool

sample was used to code MDRB colonization status at any point during 1-yr post-transplant as a binary variable. MDRB colonization data was also used to code each sample based on positive culture for CRE, Ceph-RE, and/or VRE at time of fecal sample collection.

Sequencing library preparation

The V3-V4 region of the 16S rRNA gene was amplified using standard Illumina primers (F: 5' TCGTCGGCAGCGTCAGATGTGTATAAGAGACAGCCTACGGGNGGCWGCAG, R: 5' GTCTCGTGGGCTCGGAGATGTGTATAAGAGACAGGACTACHVGGGTATCTAATCC). We purified 16S rRNA PCR products using Agencourt AMPure XP beads (Beckman Coulter) and multiplexed samples with the Illumina Nextera XT Index kit. Indexed libraries were further purified using AMPure XP beads and quantified using the Quant-iT Broad Range dsDNA Assay Kit (Thermo Fisher Scientific).

References

1. Organ Procurement and Transplantation Network: About MELD and PELD. Available at: https://optn.transplant.hrsa.gov/resources/allocation-calculators/about-meld-and-peld/ [Accessed January 9, 2017].
2. Macesic N, Gomez-Simmonds A, Sullivan SB, Giddins MJ, Ferguson SA, Korakavi G, et al. Genomic Surveillance Reveals Diversity of Multidrug-Resistant Organism Colonization and Infection: A Prospective Cohort Study in Liver Transplant Recipients. Clin Infect Dis 2018;67:905-912.

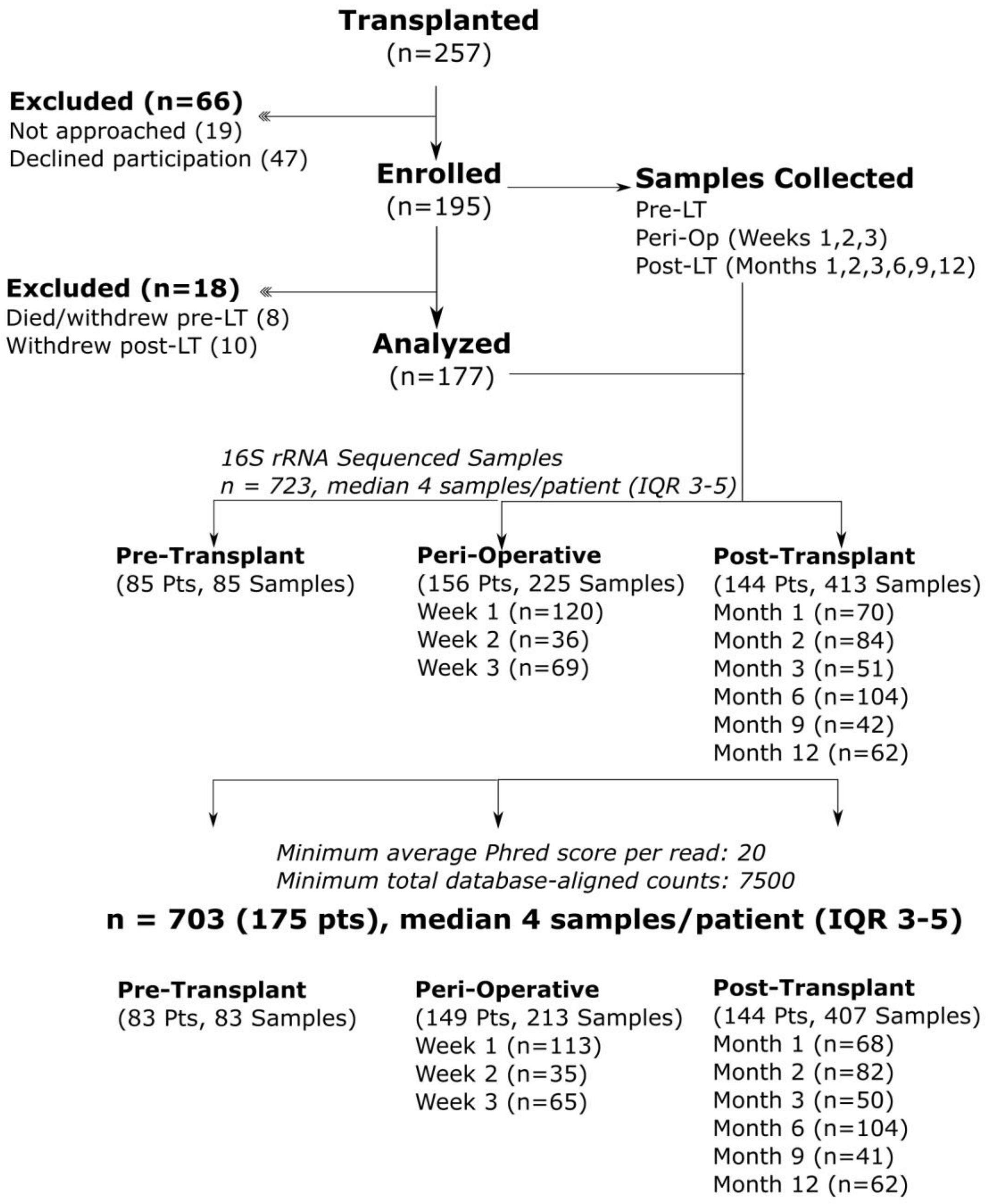

Supplementary Figure 1. Liver transplant study enrollment and sample flowchart. At our tertiary care center, 257 patients were transplanted between March 2014 and January 2017. Of these, 195 consented to our study, 18 of whom were subsequently excluded due to withdrawal or death. From the remaining 177 patients who completed 1-year follow-up after LT, we collected fecal samples at defined timepoints pre-LT, peri-LT (weeks 1, 2, and 3 after LT), and post-LT (months 1, 2, 3, 6, 9, and 12). In total we sequenced 723 fecal samples (median 4 samples per patient). After quality-filtering of sequencing data, our analysis dataset included 703 samples from 175 patients, spread across the sampling timepoints.

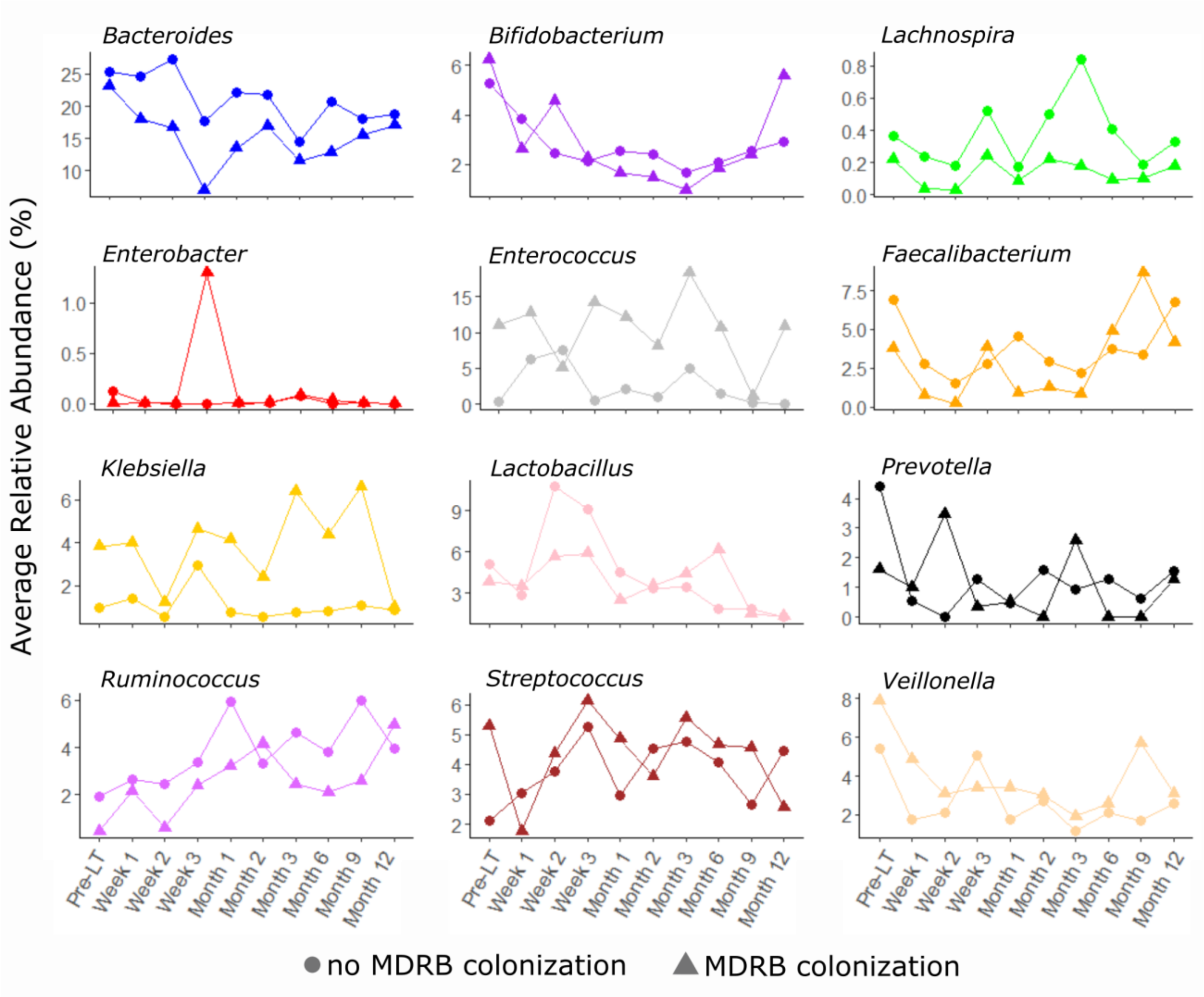

Supplementary Figure 2. Relative abundance of key genera throughout the study period in patients with or without MDRB colonization. Each plot shows the average relative abundance (%) of a specific genus in samples with (triangle) or without (circle) culture-based evidence of CRE, VRE, and/or Ceph-RE colonization at specified time categories throughout the study period. Average relative abundance of each genus is shown at pre-LT (MDRB+ n=24; MDRB- n=59), Week 1 (MDRB+ n=52; MDRB- n=61), Week 2 (MDRB+ n=21; MDRB- n=14), Week 3 (MDRB+ n=33; MDRB- n=32), Month 1 (MDRB+ n=24; MDRB- n=44), Month 2 (MDRB+ n=33; MDRB- n=49), Month 3 (MDRB+ n=14; MDRB- n=36), Month 6 (MDRB+ n=27; MDRB- n=77), Month 9 (MDRB+ n=11; MDRB- n=30), and Month 12 (MDRB+ n=15; MDRB- n=47).
