# Supplementary material for: Colonizing multidrug-resistant bacteria and the longitudinal evolution of the intestinal microbiome after liver transplantation
Source: Nat Commun. 2019 Oct 17;10:4715. doi: 10.1038/s41467-019-12633-4 (PMC6797753; doi:10.1038/s41467-019-12633-4)
Supplement: Supplementary file 3 — Description of Additional Supplementary Files [file 41467_2019_12633_MOESM3_ESM.docx]

# Supplementary Data Legends

**Supplementary Data 1.** Clinical and microbial predictors associated with gut microbiome alpha-diversity before and after liver transplantation using uni- and multivariate linear mixed-effect regression models

**Supplementary Data 2.** Univariate associations between exposure to specific antibiotic classes and gut microbiome alpha-diversity before and after liver transplantation using linear and linear mixed-effect regression models

**Supplementary Data 3.** Significantly differentially abundant gut microbiota pre-LT in patients with vs. without (baseline) alcohol-related liver disease (ARLD) using *DESeq2* (p<0.05; FDR/p_adj_<0.05)

**Supplementary Data 4.** Significantly differentially abundant gut microbiota pre-LT in patients with vs. without (baseline) alcohol-related liver disease (ARLD) using *ANCOM 2.0*

**Supplementary Data 5.** Significantly differentially abundant gut microbiota pre-LT in patients with high (above median; >=18) vs. low (baseline) model for end-stage liver disease (MELD) score at time of transplant using *DESeq2* (p<0.05; FDR/p_adj_<0.05)

**Supplementary Data 6.** Significantly differentially abundant gut microbiota pre-LT in patients with high (above median; >=18) vs. low (baseline) model for end-stage liver disease (MELD) score at time of transplant using *ANCOM 2.0*

**Supplementary Data 7.** Significantly differentially abundant gut microbiota pre-LT in patients with Child-Turcotte-Pugh (CTP) class C vs. class A (baseline) at time of transplant using *DESeq2* (p<0.05; FDR/p_adj_<0.05)

**Supplementary Data 8.** Significantly differentially abundant gut microbiota pre-LT in patients with Child-Turcotte-Pugh (CTP) class C vs. class A (baseline) at time of transplant using *ANCOM 2.0*

**Supplementary Data 9**. Constrained linear mixed-effect (CLME) results for longitudinal analysis of Shannon α-diversity by liver disease etiology

**Supplementary Data 10:** Constrained linear mixed-effect (CLME) results for longitudinal analysis of Chao α-diversity by liver disease etiology

**Supplementary Data 11.** Differential abundance analysis of gut microbiota at peri-LT (Weeks 1-3) vs. pre-LT (baseline) using *DESeq2* (p<0.05; FDR/p_adj_<0.05)

**Supplementary Data 12.** Differential abundance analysis of gut microbiota at early post-LT (Months 1-3) vs. peri-LT (Weeks 1-3; baseline) using *DESeq2* (p<0.05; FDR/p­_adj_<0.05)

**Supplementary Data 13.** Significantly differentially abundant gut microbiota at late post-LT (Months 6-12) vs. early post-LT (Months 1-3; baseline) using *DESeq2* (p<0.05; FDR/p­_adj_<0.05)

**Supplementary Data 14.** Significantly differentially abundant gut microbiota across transplant phase (pre-LT, peri-LT (Weeks 1-3), early post-LT (Months 1-3), and late post-LT (Months 6-12) stratified by primary underlying liver disease using longitudinal *ANCOM* *2.0*

**Supplementary Data 15.** Significantly differentially abundant gut microbiota pre-LT in patients who subsequently developed colonization by multidrug-resistant bacteria (MDRB) within one-year of LT vs. those who did not using *DESeq2* (p<0.05; FDR/p­_adj_<0.05)

**Supplementary Data 16.** Significantly differentially abundant gut microbiota pre-LT in patients who subsequently developed colonization by multidrug-resistant bacteria (MDRB) within one-year of LT vs. those who did not using *ANCOM* *2.0*

**Supplementary Data 17.** Significantly differentially abundant gut microbiota across all timepoints in patients with vs. without carbapenem-resistant Enterobacteriaceae (CRE) colonization at time of sample collection using *DESeq2* (p<0.05; FDR/p­_adj_<0.05)

**Supplementary Data 18.** Significantly differentially abundant gut microbiota across all timepoints in patients with vs. without carbapenem-resistant Enterobacteriaceae (CRE) colonization at time of sample collection using *ANCOM* *2.0*

**Supplementary Data 19.** Significantly differentially abundant gut microbiota across all timepoints in patients with vs. without colonization by Enterobacteriaceae resistant to third-generation cephalosporins (Ceph-RE) at time of sample collection using *DESeq2* (p<0.05; FDR/p­_adj_<0.05)

**Supplementary Data 20.** Significantly differentially abundant gut microbiota across all timepoints in patients with vs. without colonization by Enterobacteriaceae resistant to third-generation cephalosporins (Ceph-RE) at time of sample collection using *ANCOM* *2.0*

**Supplementary Data 21.** Significantly differentially abundant gut microbiota across all timepoints in patients with vs. without vancomycin-resistant enterococci (VRE) colonization at time of sample collection using *DESeq2* (p<0.05; FDR/p­_adj_<0.05)

**Supplementary Data 22.** Significantly differentially abundant gut microbiota across all timepoints in patients with vs. without vancomycin-resistant enterococci (VRE) colonization at time of sample collection using *ANCOM* *2.0*

**Supplementary Data 23.** Significantly differentially abundant gut microbiota across all timepoints in patients who developed colonization by multidrug-resistant bacteria (MDRB) within one-year of LT vs. those who did not using *DESeq2* (p<0.05; FDR/p­_adj_<0.05)

**Supplementary Data 24.** Significantly differentially abundant gut microbiota across all timepoints in patients who developed colonization by multidrug-resistant bacteria (MDRB) within one-year of LT vs. those who did not using *ANCOM* *2.0*
