# Supplementary material for: Colonizing multidrug-resistant bacteria and the longitudinal evolution of the intestinal microbiome after liver transplantation
Source: Nat Commun. 2019 Oct 17;10:4715. doi: 10.1038/s41467-019-12633-4 (PMC6797753; doi:10.1038/s41467-019-12633-4)
Supplement: Supplementary file 28 — Reporting Summary [file 41467_2019_12633_MOESM28_ESM.pdf]

nature research

Corresponding author(s): Anne-Catrin Uhlemann

Last updated by author(s): Sep 9, 2019

Reporting Summary

Nature Research wishes to improve the reproducibility of the work that we publish. This form provides structure for consistency and transparency in reporting. For further information on Nature Research policies, see Authors & Referees and the Editorial Policy Checklist.

Statistics

For all statistical analyses, confirm that the following items are present in the figure legend, table legend, main text, or Methods section.

n/a	Confirmed	
☐	☒	The exact sample size (*n*) for each experimental group/condition, given as a discrete number and unit of measurement
☐	☒	A statement on whether measurements were taken from distinct samples or whether the same sample was measured repeatedly
☐	☒	The statistical test(s) used AND whether they are one- or two-sided<br>*Only common tests should be described solely by name; describe more complex techniques in the Methods section.*
☐	☒	A description of all covariates tested
☐	☒	A description of any assumptions or corrections, such as tests of normality and adjustment for multiple comparisons
☐	☒	A full description of the statistical parameters including central tendency (e.g. means) or other basic estimates (e.g. regression coefficient) AND variation (e.g. standard deviation) or associated estimates of uncertainty (e.g. confidence intervals)
☐	☒	For null hypothesis testing, the test statistic (e.g. *F*, *t*, *r*) with confidence intervals, effect sizes, degrees of freedom and *P* value noted<br>*Give P values as exact values whenever suitable.*
☒	☐	For Bayesian analysis, information on the choice of priors and Markov chain Monte Carlo settings
☒	☐	For hierarchical and complex designs, identification of the appropriate level for tests and full reporting of outcomes
☒	☐	Estimates of effect sizes (e.g. Cohen's *d*, Pearson's *r*), indicating how they were calculated

Our web collection on statistics for biologists contains articles on many of the points above.

Software and code

Policy information about availability of computer code

Data collection	N/A
Data analysis	Custom scripts (R Markdown) and required metadata used for analyses included in this manuscript will be made publicly available at https://github.com/mka2136/lt_microbiome.

For manuscripts utilizing custom algorithms or software that are central to the research but not yet described in published literature, software must be made available to editors/reviewers. We strongly encourage code deposition in a community repository (e.g. GitHub). See the Nature Research guidelines for submitting code & software for further information.

Data

Policy information about availability of data

All manuscripts must include a data availability statement. This statement should provide the following information, where applicable:

- Accession codes, unique identifiers, or web links for publicly available datasets
- A list of figures that have associated raw data
- A description of any restrictions on data availability

Sequencing data is publicly available through the NCBI Sequencing Read Archive (SRA) (accession number SRP185798 after filtering any human-derived sequences. All relevant code and metadata used for analyses included in this manuscript, and generation of Figures 1-4, Supplementary Figure 2, and Supplementary Tables 3 – 24, is published in a public repository at https://github.com/mka2136/lt_microbiome. Data underlying Figures 1-4, Supplementary Figure 2, and Supplementary Data 3-24 are provided as Source Data files. All other data are available from the corresponding author upon reasonable requests.

Field-specific reporting

Please select the one below that is the best fit for your research. If you are not sure, read the appropriate sections before making your selection.

☒ Life sciences ☐ Behavioural & social sciences ☐ Ecological, evolutionary & environmental sciences

For a reference copy of the document with all sections, see nature.com/documents/nr-reporting-summary-flat.pdf

Life sciences study design

All studies must disclose on these points even when the disclosure is negative.

Sample size	Our sample size was predetermined, as we attempted to enroll all adult liver transplant recipients at our tertiary care center over a three-year period (2014-2017). We enrolled 177 patients in total, but our data analysis consisted of 703 samples from 175 patients (see below for Data Exclusions). We have reported all sample sizes and categorized continuous data in order to increase power.
Data exclusions	For 16S rRNA sequencing output from stool samples, we applied a minimum cutoff of 7500 counts for inclusion in the analysis. This cutoff was based on alpha-diversity rarefaction, in which subsampling at different sequencing depths showed that diversity metrics were stable beyond ~5,500-6,000 reads. In total, We sequenced 723 fecal samples; 703 samples (median 4 per patient) passed the minimum count cutoff and were included in this analysis
Replication	Each fecal sample was sequenced successfully once without replication. However, each sequencing run included one library constructed using the ZymoBIOMICS Microbial Community Standards, which were analyzed in order to confirm consistency in output across sequencing runs. To ensure reproducibility of our data analyses, we have uploaded all raw FASTQ sequence files for each sample, along with a publicly available repository including the code and metadata used to generate figures and tables in our manuscript.
Randomization	Samples were not randomized/allocated into experimental groups. This was a prospective cohort, and our longitudinal analyses included both microbial and clinical predictors of microbiome diversity and taxonomic makeup. We constructed multivariate linear mixed-regression models in order to control for relevant covariates and potential confounders.
Blinding	Stool DNA extraction and sequencing library preparation were performed by lab members who did not have access to patient metadata. Key patient phenotypic metadata (i.e. underlying liver disease etiology, post-transplant complications) was independently coded by two infectious diseases physicians and adjudicated by a liver transplant physician.

Reporting for specific materials, systems and methods

We require information from authors about some types of materials, experimental systems and methods used in many studies. Here, indicate whether each material, system or method listed is relevant to your study. If you are not sure if a list item applies to your research, read the appropriate section before selecting a response.

Materials & experimental systems

n/a	Involved in the study
☒	☐ Antibodies
☒	☐ Eukaryotic cell lines
☒	☐ Palaeontology
☒	☐ Animals and other organisms
☐	☒ Human research participants
☒	☐ Clinical data

Methods

n/a	Involved in the study
☒	☐ ChIP-seq
☒	☐ Flow cytometry
☒	☐ MRI-based neuroimaging

Human research participants

Policy information about studies involving human research participants

Population characteristics	We enrolled 195 patients pre-transplant, of whom 177 completed 1-year post-transplant follow-up (Supplementary Figure 1). Patient characteristics are summarized in Table 1. The majority of patients were male (60%) and the median age was 60 years. HCV was the most common reason for transplant (n=71, 40%), followed by NAFLD (n=30, 17%) and ARLD (n=19, 11%), and 69 patients (39%) had concurrent hepatocellular carcinoma (HCC). The median MELD was 18 (IQR 13 – 24) and median CTP score was 9 (IQR 7 – 11) at the time of LT. A high proportion of patients (65%) developed intestinal MDRO colonization at least once over the 1-year study period. This included colonization by CRE (n=31, 18%), Ceph-RE (n=83, 47%), and/or VRE (n=77, 44%).
Recruitment	We prospectively recruited consecutive adult patients (age ≥ 18 years) undergoing LT at a tertiary care hospital between March 2014 and January 2017. Potential biases include the fact that this was a single-center study, and LT cohorts, procedures, and outcomes may differ at other centers. Second, not all patients transplanted during the study period at our center participated; however, enrolled and non-enrolled patients did not differ in their clinical and demographic characteristics.

Ethics oversight	Study procedures were approved by the Columbia University Irving Medical Center Institutional Review Board (IRB-AAAM7704)

Note that full information on the approval of the study protocol must also be provided in the manuscript.
