# Supplementary material for: Colonizing multidrug-resistant bacteria and the longitudinal evolution of the intestinal microbiome after liver transplantation
Source: Nat Commun. 2019 Oct 17;10:4715. doi: 10.1038/s41467-019-12633-4 (PMC6797753; doi:10.1038/s41467-019-12633-4)
Supplement: Supplementary file 29 — Source Data [file 41467_2019_12633_MOESM29_ESM.zip › Source_Data/Differential_Abundance.pdf]

Liver transplant microbiome and MDRO

Medini K. Annavajhala

July 24, 2019

Differential abundance testing using *DESeq2* and Analysis of Composition of Microbiomes (*ANCOM*)

This R Markdown document is part of a series used to analyze data and generate figures for the citation below. The current document includes all code and options used to run differential abundance testing referenced in the manuscript, using both `DESeq2` and `ANCOM 2.0`. These outputs are also provided as Supplementary Data files 3-8 and 11-24 with the citation below.

Title:

Colonizing multidrug-resistant organisms and the longitudinal evolution of the intestinal microbiome after liver transplantation

Authors:

Medini K. Annavajhala, Angela Gomez-Simmonds, Nenad Macesic, Sean B. Sullivan, Anna Kress, Sabrina D. Khan, Marla J. Giddins, Stephania Stump, Grace I. Kim, Ryan Narain, Elizabeth C. Verna, Anne-Catrin Uhlemann

Journal:

Nature communications **2019**

Load Required Libraries and Functions:

```
library("phyloseq"); packageVersion("phyloseq")
```

```
## [1] '1.28.0'
```

```
library("DESeq2"); packageVersion("DESeq2")
```

```
## [1] '1.24.0'
```

```
library("exactRankTests"); packageVersion("exactRankTests")
```

```
## [1] '0.8.30'
```

```
library("nlme"); packageVersion("nlme")
```

```
## [1] '3.1.140'
```

```
library("dplyr"); packageVersion("dplyr")
```

```
## [1] '0.8.3'
```

```
library("reshape2"); packageVersion("reshape2")
```

```
## [1] '1.4.3'
```

```
source("inputs/ANCOM_updated_code.R", echo=F)

gm_mean = function(x, na.rm=TRUE){
```

```r
  exp(sum(log(x[x > 0]), na.rm=na.rm) / length(x))
}
```

Load data:

```r
LT_data <- read.table("inputs/differential_abundance_metadata.txt", header=T)
colnames(LT_data)
```

```
##  [1] "StoolID"           "StudyID"           "Time_Category"
##  [4] "Time_Category2"    "Primary_Diagnosis" "ARLD"
##  [7] "MELD2016_cat"      "CTP_Class"         "ESBL_sample"
## [10] "CRE_sample"        "VRE_sample"        "MDRO_sample"
## [13] "MDRO_within1yr"
```

```r
# Time_Category: One of 10 pre-selected sampling timepoints; pre-LT, then
#                    Weeks 1, 2, 3 and Months 1, 2, 3, 6, 9, and 12 post-LT
#
# Time_Category2: Timepoints split into 4 periods; pre-LT, peri-LT (Weeks 1-3),
#                    early post-LT (Months 1-3), late post-LT (Months 6-12)
#
# Primary_Diagnosis: primary underlying liver disease etiology (reason for LT)
#        AIH: Autoimmune hepatitis
#        ARLD: Alcohol-related liver disease
#        BILIARY: Etiologies related to biliary complications
#        HBV: Hepatitis B virus
#        HCV: Hepatitis C virus
#        NAFLD: Non-alcoholic fatty liver disease
#        PCLD: Polycystic liver/kidney disease
#        OTHER
#
# ARLD: binary variable; yes/no for ARLD as the primary indication
#
# MELD2016_cat: above/below median MELD (18)
#
# CTP_Class: Standard definitions based on CTP score; A (5-6), B (7-9), C(10-13)
#
# ESBL_sample: binary (0=no; 1=yes) variable indicating whether the stool sample was
#               culture-positive for Enterobacteriaceae resistant to third-generation
#               cephalosporins and beta lactam/beta lactamase inhibitor combinations
#               (Ceph-RE), sometimes referred to as ESBLs (extended-spectrum beta-lactam
#               producers)
#
# CRE_sample: binary (0=no; 1=yes) variable indicating whether the stool sample was
#              culture-positive for carbapenem-resistant Enterobacteriaceae (CRE)
#
# VRE_sample: binary (0=no; 1=yes) variable indicating whether the stool sample was
#              culture-positive for vancomycin-resistant enterococci (VRE)
#
# MDRO_sample: binary (0=no; 1=yes) variable indicating whether the stool sample was
#              culture-positive for CRE, VRE, and/or Ceph-RE
#
# MDRO_within1yr: binary (0=no; 1=yes) variable indicating whether the patient associated
#                  with the sample was culture-positive for CRE, VRE, and/or Ceph-RE at any
#                  point up to 1-yr post-LT
```

```r
# Read in phyloseq object (see Phyloseq_Objects.Rmd)
LT_phylo = readRDS("inputs/phylo_filtered.RDS")

df <- data.frame(sample_data(LT_phylo),
                 "StoolID"=rownames(sample_data(LT_phylo))) #Sample names
merged <- merge(df, LT_data, by="StoolID") #Merge metadata with phyloseq object
merged <- sample_data(merged)
sample_names(merged) = merged$StoolID #fix names
sample_data(LT_phylo) <- sample_data(merged)
```

DESeq2

First, we ran DESeq2 to identify potentially differentially abundant taxa across several comparisons.

Supplementary Data 3. Alcohol-related liver disease (ARLD) and pre-LT microbiota

Shannon α-diversity was significantly lower in ARLD patients pre-LT than most other diagnoses (see Figure 2 and Table 2 in the manuscript). We wanted to see which taxa may be differentially abundance in these patients, contributing to their dysbiotic microbiome.

```r
LT_pre = prune_samples(sample_data(LT_phylo)$Time_Category == "a-Pre", LT_phylo)
sample_data(LT_pre)$ARLD = factor(sample_data(LT_pre)$ARLD, levels=c("OTHER","ARLD"))
dds = phyloseq_to_deseq2(LT_pre, ~ARLD) # Here, we just look at ARLD as a binary variable
geoMeans = apply(counts(dds), 1, gm_mean) # Calculate geometric means
dds = estimateSizeFactors(dds, geoMeans = geoMeans)
dds = estimateDispersions(dds)
vst = getVarianceStabilizedData(dds)
deseq = DESeq(dds,fitType="local") # Run DESeq2
resultsNames(deseq) # Should show the comparison of interest (ARLD_vs_OTHER)
```

```
## [1] "Intercept"          "ARLD_ARLD_vs_OTHER"
```

```r
res_arld = results(deseq)
res_arld = res_arld[order(res_arld$padj, na.last=NA),]
res_arld_tax = cbind(as(res_arld, "data.frame"),
                     as(tax_table(LT_pre)[rownames(res_arld), ],
                        "matrix")) # Merge in taxonomic information
alpha = 0.05 # Significant cut-off for FDR-adjusted p-value
sig_arld_tax = res_arld_tax[(res_arld_tax$padj<alpha),]
head(sig_arld_tax)
```

```
##        baseMean log2FoldChange    lfcSE       stat       pvalue
## 186521 31.41782      -21.25718 1.902194 -11.175085 5.399882e-29
## 317135 27.31885      -21.06878 2.128017  -9.900662 4.135301e-23
## 561171 36.44898      -20.31002 2.123464  -9.564567 1.126718e-21
## 359044 47.77328      -21.12638 2.679639  -7.884040 3.169626e-15
## 215670 65.79683      -27.96448 3.626060  -7.712085 1.237782e-14
## 310608 15.72093      -20.34080 2.640981  -7.701990 1.339638e-14
##                padj  Kingdom        Phylum       Class         Order
## 186521 1.387770e-26 Bacteria    Firmicutes  Clostridia Clostridiales
## 317135 5.313862e-21 Bacteria    Firmicutes  Clostridia Clostridiales
## 561171 9.652218e-20 Bacteria    Firmicutes  Clostridia Clostridiales
## 359044 2.036485e-13 Bacteria    Firmicutes  Clostridia Clostridiales
## 215670 5.738115e-13 Bacteria Bacteroidetes Bacteroidia Bacteroidales
```

```
## 310608 5.738115e-13 Bacteria    Firmicutes  Clostridia Clostridiales
##                 Family      Genus Species    Genus_Species
## 186521 Ruminococcaceae       <NA>    <NA>               NA
## 317135 Ruminococcaceae       <NA>    <NA>               NA
## 561171 Lachnospiraceae       <NA>    <NA>               NA
## 359044 Lachnospiraceae    Blautia    <NA>          Blautia
## 215670  Prevotellaceae Prevotella   copri Prevotella copri
## 310608 Lachnospiraceae      Dorea    <NA>            Dorea
```

```
write.table(sig_arld_tax,"differential_abundance/Supp_Data3.DESeq2_ARLD_preLT.txt", sep="\t")
```

We found 83 differentially abundant taxa **pre-LT** in patients with vs. without ARLD.

Supplementary Data 5. MELD at LT and pre-LT microbiota

```
sample_data(LT_pre)$MELD2016_cat = factor(sample_data(LT_pre)$MELD2016_cat, levels=c("LOW","HIGH"))
dds = phyloseq_to_deseq2(LT_pre, ~MELD2016_cat) # Here, we just look at meld as a binary variable
geoMeans = apply(counts(dds), 1, gm_mean) # Calculate geometric means
dds = estimateSizeFactors(dds, geoMeans = geoMeans)
dds = estimateDispersions(dds)
vst = getVarianceStabilizedData(dds)
deseq = DESeq(dds,fitType="local") # Run DESeq2
# resultsNames(deseq)
res_meld = results(deseq)
res_meld = res_meld[order(res_meld$padj, na.last=NA),]
res_meld_tax = cbind(as(res_meld, "data.frame"),
                     as(tax_table(LT_pre)[rownames(res_meld), ],
                        "matrix")) # Merge in taxonomic information
alpha = 0.05 # Significant cut-off for FDR-adjusted p-value
sig_meld_tax = res_meld_tax[(res_meld_tax$padj<alpha),]
head(sig_meld_tax)
```

```
##            baseMean log2FoldChange     lfcSE      stat       pvalue
## 264552     4.884131     -23.680878 2.9048087 -8.152302 3.570605e-16
## 590982  2000.703799       7.025420 0.8873491  7.917313 2.426992e-15
## 579608   323.463161       4.723885 0.7333410  6.441594 1.182255e-10
## 4446943  134.553874       5.688799 0.9012110  6.312394 2.747523e-10
## 508968   117.428207      -5.295776 0.8916515 -5.939289 2.862609e-09
## 4422456  165.839543       5.054034 0.9016807  5.605126 2.081037e-08
##                 padj  Kingdom     Phylum      Class           Order
## 264552  1.506795e-13 Bacteria Firmicutes Clostridia   Clostridiales
## 590982  5.120953e-13 Bacteria Firmicutes    Bacilli Lactobacillales
## 579608  1.663038e-08 Bacteria Firmicutes    Bacilli Lactobacillales
## 4446943 2.898637e-08 Bacteria Firmicutes Clostridia   Clostridiales
## 508968  2.416042e-07 Bacteria Firmicutes Clostridia   Clostridiales
## 4422456 1.463663e-06 Bacteria Firmicutes Clostridia   Clostridiales
##                   Family         Genus Species      Genus_Species
## 264552   Veillonellaceae     Dialister    <NA>          Dialister
## 590982   Enterococcaceae  Enterococcus    <NA>       Enterococcus
## 579608  Streptococcaceae Streptococcus    <NA>      Streptococcus
## 4446943  Veillonellaceae    Veillonella  dispar Veillonella dispar
## 508968   Lachnospiraceae     Roseburia    <NA>          Roseburia
## 4422456  Veillonellaceae    Veillonella  dispar Veillonella dispar
```

```
write.table(sig_meld_tax,"differential_abundance/Supp_Data5.DESeq2_MELD_preLT.txt", sep="\t")
```

Supplementary Data 7. CTP class at LT and pre-LT microbiota

```
sample_data(LT_pre)$CTP_Class = factor(sample_data(LT_pre)$CTP_Class, levels=c("A","B","C"))
dds = phyloseq_to_deseq2(LT_pre, ~CTP_Class) # Here, we just look at ctp as a binary variable
geoMeans = apply(counts(dds), 1, gm_mean) # Calculate geometric means
dds = estimateSizeFactors(dds, geoMeans = geoMeans)
dds = estimateDispersions(dds)
vst = getVarianceStabilizedData(dds)
deseq = DESeq(dds,fitType="local") # Run DESeq2
# resultsNames(deseq) # Should show the comparison of interest (C_vs_A)
res_ctp = results(deseq, contrast=c("CTP_Class","C","A"))
res_ctp = res_ctp[order(res_ctp$padj, na.last=NA),]
res_ctp_tax = cbind(as(res_ctp, "data.frame"),
                    as(tax_table(LT_pre)[rownames(res_ctp), ],
                       "matrix")) # Merge in taxonomic information
alpha = 0.05 # Significant cut-off for FDR-adjusted p-value
sig_ctp_tax = res_ctp_tax[(res_ctp_tax$padj<alpha),]
head(sig_ctp_tax)
```

```
##          baseMean log2FoldChange     lfcSE     stat       pvalue
## 590982  4031.0800      10.744818 1.1365296 9.454059 3.259401e-21
## 4296244  744.8991       7.375852 1.1990982 6.151166 7.691549e-10
## 1995182 1860.4934       7.576337 1.2689324 5.970639 2.363258e-09
## 518033   133.7377       9.407053 1.5939209 5.901832 3.594869e-09
## 579608   356.8502       5.604191 0.9611237 5.830874 5.513788e-09
## 4422456  165.8395       7.133611 1.2401518 5.752208 8.808549e-09
##                 padj  Kingdom     Phylum      Class           Order
## 590982  1.469990e-18 Bacteria Firmicutes    Bacilli Lactobacillales
## 4296244 1.734444e-07 Bacteria Firmicutes Clostridia   Clostridiales
## 1995182 3.552765e-07 Bacteria Firmicutes    Bacilli Lactobacillales
## 518033  4.053215e-07 Bacteria Firmicutes    Bacilli Lactobacillales
## 579608  4.973437e-07 Bacteria Firmicutes    Bacilli Lactobacillales
## 4422456 6.621093e-07 Bacteria Firmicutes Clostridia   Clostridiales
##                   Family         Genus Species      Genus_Species
## 590982   Enterococcaceae  Enterococcus    <NA>       Enterococcus
## 4296244  Veillonellaceae   Veillonella  dispar Veillonella dispar
## 1995182 Lactobacillaceae Lactobacillus    <NA>      Lactobacillus
## 518033  Lactobacillaceae Lactobacillus    zeae Lactobacillus zeae
## 579608  Streptococcaceae Streptococcus    <NA>      Streptococcus
## 4422456  Veillonellaceae   Veillonella  dispar Veillonella dispar
```

```
write.table(sig_ctp_tax,"differential_abundance/Supp_Data7.DESeq2_CTP_CvA_preLT.txt", sep="\t")
```

Supplementary Data 11, 12, 13. Longitudinal changes in microbiota stratified by primary disease etiology

The next example is a little more complicated. We wanted to see which taxa were enriched at different phases of the study period (pre-LT, peri-LT, early post-LT, and late post-LT), while controlling for the primary underlying liver disease etiology.

```
LT_phylo_diags = prune_samples(sample_data(LT_phylo)$Primary_Diagnosis %in%
                c("AIH","ARLD","BILIARY","HBV","HCV","NAFLD"), LT_phylo)
sample_data(LT_phylo_diags)$Time_Category2 = factor(sample_data(LT_phylo_diags)$Time_Category2)
sample_data(LT_phylo_diags)$Primary_Diagnosis = factor(sample_data(LT_phylo_diags)$Primary_Diagnosis)
dds = phyloseq_to_deseq2(LT_phylo_diags,
                         ~ Time_Category2 +
```

```r
                           Primary_Diagnosis)
geoMeans = apply(counts(dds), 1, gm_mean)
dds = estimateSizeFactors(dds, geoMeans = geoMeans)
dds = estimateDispersions(dds)
vst = getVarianceStabilizedData(dds)
deseq = DESeq(dds, fitType="local")
resultsNames(deseq)
```

```
## [1] "Intercept"
## [2] "Time_Category2_b.Peri_vs_a.Pre"
## [3] "Time_Category2_c.Post_M1to3_vs_a.Pre"
## [4] "Time_Category2_d.Post_M6to12_vs_a.Pre"
## [5] "Primary_Diagnosis_ARLD_vs_AIH"
## [6] "Primary_Diagnosis_BILIARY_vs_AIH"
## [7] "Primary_Diagnosis_HBV_vs_AIH"
## [8] "Primary_Diagnosis_HCV_vs_AIH"
## [9] "Primary_Diagnosis_NAFLD_vs_AIH"
```

```r
#First, peri- v pre-LT
res_peripre = results(deseq,
                      contrast = c("Time_Category2","b-Peri","a-Pre"))
res_peripre = res_peripre[order(res_peripre$padj, na.last=NA),]
res_peripre_tax = cbind(as(res_peripre, "data.frame"),
                        as(tax_table(LT_phylo_diags)[rownames(res_peripre), ],
                           "matrix"))
sig_peripre_tax = res_peripre_tax[(res_peripre_tax$padj<alpha),]
head(sig_peripre_tax)
```

```
##          baseMean log2FoldChange     lfcSE      stat       pvalue
## 526682  29.550384       3.686938 0.4428301  8.325853 8.372388e-17
## 295296  16.171823      12.362267 1.5410104  8.022182 1.038828e-15
## 527413   6.372549      17.473413 2.3186891  7.535902 4.849724e-14
## 3864823 23.567599      -8.600008 1.1473617 -7.495464 6.606419e-14
## 342427  15.205184      -4.987388 0.6917129 -7.210200 5.586982e-13
## 313143  28.197885       6.415430 0.8991084  7.135324 9.655913e-13
##                 padj  Kingdom         Phylum          Class
## 526682  5.642990e-14 Bacteria Actinobacteria Actinobacteria
## 295296  3.500850e-13 Bacteria     Firmicutes     Clostridia
## 527413  1.089571e-11 Bacteria     Firmicutes     Clostridia
## 3864823 1.113182e-11 Bacteria     Firmicutes     Clostridia
## 342427  7.531252e-11 Bacteria     Firmicutes     Clostridia
## 313143  1.084681e-10 Bacteria     Firmicutes     Clostridia
##                   Order                Family       Genus Species
## 526682  Actinomycetales      Actinomycetaceae Actinomyces    <NA>
## 295296    Clostridiales                  <NA>        <NA>    <NA>
## 527413    Clostridiales       Lachnospiraceae Coprococcus    <NA>
## 3864823   Clostridiales       Veillonellaceae Veillonella  dispar
## 342427    Clostridiales       Veillonellaceae Veillonella  dispar
## 313143    Clostridiales Peptostreptococcaceae        <NA>    <NA>
##              Genus_Species
## 526682         Actinomyces
## 295296                  NA
## 527413         Coprococcus
## 3864823 Veillonella dispar
```

```
## 342427  Veillonella dispar
## 313143                  NA
```

```
write.table(sig_peripre_tax,"differential_abundance/Supp_Data11.DESeq2_peri_v_preLT.txt", sep="\t")

#Then, early post-LT vs peri-LT
res_earlypostperi = results(deseq,
                      contrast = c("Time_Category2","c-Post_M1to3","b-Peri"))
res_earlypostperi = res_earlypostperi[order(res_earlypostperi$padj, na.last=NA),]
res_earlypostperi_tax = cbind(as(res_earlypostperi, "data.frame"),
                        as(tax_table(LT_phylo_diags)[rownames(res_earlypostperi), ],
                           "matrix"))
sig_earlypostperi_tax = res_earlypostperi_tax[(res_earlypostperi_tax$padj<alpha),]
head(sig_earlypostperi_tax)
```

```
##           baseMean log2FoldChange     lfcSE      stat       pvalue
## 175617   14.967165      56.086045 3.8318345 14.636865 1.634315e-48
## 176750    1.628787      41.467774 3.8320481 10.821308 2.728544e-27
## 548503   18.956831       6.136158 0.7757851  7.909610 2.581966e-15
## 784256    6.866915      24.596232 3.1625202  7.777415 7.402137e-15
## 3801267 130.756877       3.846643 0.5150997  7.467766 8.156801e-14
## 290529   69.468793       5.069034 0.7178961  7.060958 1.653586e-12
##                 padj  Kingdom        Phylum       Class         Order
## 175617  1.183244e-45 Bacteria Bacteroidetes Bacteroidia Bacteroidales
## 176750  9.877330e-25 Bacteria    Firmicutes  Clostridia Clostridiales
## 548503  6.231146e-13 Bacteria    Firmicutes  Clostridia Clostridiales
## 784256  1.339787e-12 Bacteria    Firmicutes  Clostridia Clostridiales
## 3801267 1.181105e-11 Bacteria    Firmicutes  Clostridia Clostridiales
## 290529  1.995327e-10 Bacteria    Firmicutes  Clostridia Clostridiales
##                   Family        Genus  Species       Genus_Species
## 175617    Bacteroidaceae  Bacteroides     <NA>         Bacteroides
## 176750  Ruminococcaceae Ruminococcus     <NA>        Ruminococcus
## 548503  Lachnospiraceae         <NA>     <NA>                  NA
## 784256  Lachnospiraceae         <NA>     <NA>                  NA
## 3801267 Veillonellaceae  Veillonella  parvula Veillonella parvula
## 290529  Lachnospiraceae      Blautia producta    Blautia producta
```

```
write.table(sig_earlypostperi_tax,"differential_abundance/Supp_Data12.DESeq2_earlypost_v_periLT.txt", s

#Lastly, late vs early post-LT
res_latevearlypostLT = results(deseq,
                      contrast = c("Time_Category2","d-Post_M6to12","c-Post_M1to3"))
res_latevearlypostLT = res_latevearlypostLT[order(res_latevearlypostLT$padj, na.last=NA),]
res_latevearlypostLT_tax = cbind(as(res_latevearlypostLT, "data.frame"),
                        as(tax_table(LT_phylo_diags)[rownames(res_latevearlypostLT), ],
                           "matrix"))
sig_latevearlypostLT_tax = res_latevearlypostLT_tax[(res_latevearlypostLT_tax$padj<alpha),]
head(sig_latevearlypostLT_tax)
```

```
##          baseMean log2FoldChange    lfcSE      stat       pvalue
## 295296  16.171823      11.037198 1.185362  9.311248 1.263387e-20
## 182723   7.207160       9.868083 1.238662  7.966728 1.629311e-15
## 1127530  5.038622     -11.450023 1.553353 -7.371168 1.691395e-13
## 4335293  1.709828      26.377260 3.818816  6.907183 4.943721e-12
## 4326870 20.040604      11.527316 1.792080  6.432367 1.256320e-10
```

```
## 360329   4.896736       7.077849 1.123997  6.297034 3.033944e-10
##                 padj  Kingdom        Phylum       Class         Order
## 295296  8.944781e-18 Bacteria    Firmicutes  Clostridia Clostridiales
## 182723  5.767763e-13 Bacteria    Firmicutes  Clostridia Clostridiales
## 1127530 3.991692e-11 Bacteria    Firmicutes  Clostridia Clostridiales
## 4335293 8.750387e-10 Bacteria Bacteroidetes Bacteroidia Bacteroidales
## 4326870 1.778950e-08 Bacteria    Firmicutes  Clostridia Clostridiales
## 360329  3.580054e-08 Bacteria    Firmicutes  Clostridia Clostridiales
##                  Family       Genus Species Genus_Species
## 295296             <NA>        <NA>    <NA>            NA
## 182723  Lachnospiraceae        <NA>    <NA>            NA
## 1127530  Clostridiaceae Clostridium    <NA>   Clostridium
## 4335293  Bacteroidaceae Bacteroides    <NA>   Bacteroides
## 4326870 Veillonellaceae   Dialister    <NA>     Dialister
## 360329  Lachnospiraceae        <NA>    <NA>            NA
```

```
write.table(sig_latevearlypostLT_tax,"differential_abundance/Supp_Data13.DESeq2_late_v_earlypostLT.txt"
```

For example, we found 151 differentially abundant taxa between peri-LT and pre-LT samples, controlling for liver disease.

Supplementary Data 15. Pre-LT microbiota differentially abundant in patients who develop MDRO colonization

We wanted to see which taxa may be differentially abundant **pre-LT** in patients who develop MDRO colonization at any point up to 1 year post-LT.

```
sample_data(LT_pre)$MDRO_within1yr = factor(sample_data(LT_pre)$MDRO_within1yr)
dds = phyloseq_to_deseq2(LT_pre, ~MDRO_within1yr)
geoMeans = apply(counts(dds), 1, gm_mean) # Calculate geometric means
dds = estimateSizeFactors(dds, geoMeans = geoMeans)
dds = estimateDispersions(dds)
vst = getVarianceStabilizedData(dds)
deseq = DESeq(dds,fitType="local") # Run DESeq2
# resultsNames(deseq) # Should show the comparison of interest (1_vs_0)
res_mdro_1yr = results(deseq)
res_mdro_1yr = res_mdro_1yr[order(res_mdro_1yr$padj, na.last=NA),]
res_mdro_1yr_tax = cbind(as(res_mdro_1yr, "data.frame"),
                         as(tax_table(LT_pre)[rownames(res_mdro_1yr), ],
                            "matrix")) # Merge in taxonomic information
alpha = 0.05 # Significant cut-off for FDR-adjusted p-value
sig_mdro_1yr_tax = res_mdro_1yr_tax[(res_mdro_1yr_tax$padj<alpha),]
head(sig_mdro_1yr_tax)
```

```
##          baseMean log2FoldChange     lfcSE     stat       pvalue
## 590982  1155.2162       6.997109 0.9735824 7.186972 6.624421e-13
## 4422456  255.9842       7.020869 1.0332966 6.794631 1.085899e-11
## 4446943  152.6635       6.475942 1.0347201 6.258642 3.883444e-10
## 137580   219.4983       8.341716 1.5158365 5.503045 3.732871e-08
## 579608   360.3891       4.620336 0.8355545 5.529664 3.208442e-08
## 588216  1638.1491       4.441344 0.8493192 5.229299 1.701539e-07
##                 padj  Kingdom         Phylum               Class
## 590982  2.497407e-10 Bacteria     Firmicutes             Bacilli
## 4422456 2.046919e-09 Bacteria     Firmicutes          Clostridia
## 4446943 4.880194e-08 Bacteria     Firmicutes          Clostridia
## 137580  2.814584e-06 Bacteria     Firmicutes             Bacilli
```

```
## 579608  2.814584e-06 Bacteria     Firmicutes             Bacilli
## 588216  1.069134e-05 Bacteria Proteobacteria Gammaproteobacteria
##                    Order               Family         Genus Species
## 590982    Lactobacillales    Enterococcaceae  Enterococcus    <NA>
## 4422456     Clostridiales    Veillonellaceae   Veillonella  dispar
## 4446943     Clostridiales    Veillonellaceae   Veillonella  dispar
## 137580    Lactobacillales   Lactobacillaceae Lactobacillus    <NA>
## 579608    Lactobacillales   Streptococcaceae Streptococcus    <NA>
## 588216  Enterobacteriales Enterobacteriaceae          <NA>    <NA>
##              Genus_Species
## 590982        Enterococcus
## 4422456 Veillonella dispar
## 4446943 Veillonella dispar
## 137580       Lactobacillus
## 579608       Streptococcus
## 588216                  NA
```

```
write.table(sig_mdro_1yr_tax,"differential_abundance/Supp_Data15.DESeq2_preLT_MDRO_1yr.txt", sep="\t")
```

Supplementary Data 17, 19, 21, and 23. Microbiota differentially abundant in patients with colonizing CRE, Ceph-RE, VRE, and any MDRO (all time-points)

First, we wanted to see which taxa may be differentially abundant in patients during CRE colonization.

```
sample_data(LT_phylo)$CRE_sample = factor(sample_data(LT_phylo)$CRE_sample)
dds = phyloseq_to_deseq2(LT_phylo, ~CRE_sample)
geoMeans = apply(counts(dds), 1, gm_mean) # Calculate geometric means
dds = estimateSizeFactors(dds, geoMeans = geoMeans)
dds = estimateDispersions(dds)
vst = getVarianceStabilizedData(dds)
deseq = DESeq(dds,fitType="local") # Run DESeq2
# resultsNames(deseq) # Should show the comparison of interest (1_vs_0)
res_cre = results(deseq)
res_cre = res_cre[order(res_cre$padj, na.last=NA),]
res_cre_tax = cbind(as(res_cre, "data.frame"),
                     as(tax_table(LT_phylo)[rownames(res_cre), ],
                        "matrix")) # Merge in taxonomic information
alpha = 0.05 # Significant cut-off for FDR-adjusted p-value
sig_cre_tax = res_cre_tax[(res_cre_tax$padj<alpha),]
head(sig_cre_tax)
```

```
##          baseMean log2FoldChange     lfcSE      stat       pvalue
## 335701 1712.30574      -4.084598 0.4647300 -8.789184 1.506501e-18
## 533969  156.33969      -4.867284 0.5674452 -8.577541 9.692324e-18
## 317205  215.25224      -5.565628 0.7297027 -7.627254 2.398070e-14
## 357312   20.22590      -7.882233 1.0412658 -7.569857 3.736364e-14
## 851865   99.07955      -5.289650 0.7197860 -7.348921 1.998138e-13
## 310608   63.72334      -6.179776 0.8698211 -7.104652 1.206262e-12
##                padj  Kingdom     Phylum      Class         Order
## 335701 9.852513e-16 Bacteria Firmicutes Clostridia Clostridiales
## 533969 3.169390e-15 Bacteria Firmicutes Clostridia Clostridiales
## 317205 5.227792e-12 Bacteria Firmicutes Clostridia Clostridiales
## 357312 6.108955e-12 Bacteria Firmicutes Clostridia Clostridiales
## 851865 2.613565e-11 Bacteria Firmicutes Clostridia Clostridiales
## 310608 1.314826e-10 Bacteria Firmicutes Clostridia Clostridiales
```

```
##                  Family             Genus     Species
## 335701 Lachnospiraceae   [Ruminococcus]      gnavus
## 533969 Lachnospiraceae              <NA>        <NA>
## 317205 Lachnospiraceae           Blautia        <NA>
## 357312 Lachnospiraceae              <NA>        <NA>
## 851865 Ruminococcaceae Faecalibacterium prausnitzii
## 310608 Lachnospiraceae             Dorea        <NA>
##                     Genus_Species
## 335701        [Ruminococcus] gnavus
## 533969                         NA
## 317205                    Blautia
## 357312                         NA
## 851865 Faecalibacterium prausnitzii
## 310608                      Dorea
```

```
write.table(sig_cre_tax,"differential_abundance/Supp_Data17.DESeq2_CRE.txt", sep="\t")
```

Next, we wanted to identify differentially abundant taxa in patients during Ceph-RE colonization.

```
sample_data(LT_phylo)$ESBL_sample = factor(sample_data(LT_phylo)$ESBL_sample)
dds = phyloseq_to_deseq2(LT_phylo, ~ESBL_sample)
geoMeans = apply(counts(dds), 1, gm_mean) # Calculate geometric means
dds = estimateSizeFactors(dds, geoMeans = geoMeans)
dds = estimateDispersions(dds)
vst = getVarianceStabilizedData(dds)
deseq = DESeq(dds,fitType="local") # Run DESeq2
# resultsNames(deseq) # Should show the comparison of interest (1_vs_0)
res_cephre = results(deseq)
res_cephre = res_cephre[order(res_cephre$padj, na.last=NA),]
res_cephre_tax = cbind(as(res_cephre, "data.frame"),
                       as(tax_table(LT_phylo)[rownames(res_cephre), ],
                          "matrix")) # Merge in taxonomic information
alpha = 0.05 # Significant cut-off for FDR-adjusted p-value
sig_cephre_tax = res_cephre_tax[(res_cephre_tax$padj<alpha),]
head(sig_cephre_tax)
```

```
##           baseMean log2FoldChange     lfcSE      stat       pvalue
## 345362 7946.038730       3.234296 0.3362758  9.617986 6.713262e-22
## 588216 4147.227440       2.988939 0.3118708  9.583903 9.344570e-22
## 297057  227.209118      -4.363408 0.4529859 -9.632548 5.826616e-22
## 518002   29.025382       3.774284 0.4054713  9.308387 1.297888e-20
## 581782    8.655817       4.493068 0.4830942  9.300604 1.396503e-20
## 813217 2859.743633       3.256169 0.3679494  8.849501 8.791111e-19
##                padj  Kingdom         Phylum               Class
## 345362 2.248926e-19 Bacteria Proteobacteria Gammaproteobacteria
## 588216 2.248926e-19 Bacteria Proteobacteria Gammaproteobacteria
## 297057 2.248926e-19 Bacteria  Bacteroidetes         Bacteroidia
## 518002 2.016550e-18 Bacteria Proteobacteria Gammaproteobacteria
## 581782 2.016550e-18 Bacteria Proteobacteria Gammaproteobacteria
## 813217 1.057864e-16 Bacteria Proteobacteria Gammaproteobacteria
##                    Order             Family       Genus Species
## 345362 Enterobacteriales Enterobacteriaceae        <NA>    <NA>
## 588216 Enterobacteriales Enterobacteriaceae        <NA>    <NA>
## 297057     Bacteroidales     Bacteroidaceae Bacteroides    <NA>
## 518002 Enterobacteriales Enterobacteriaceae        <NA>    <NA>
```

```
## 581782 Enterobacteriales Enterobacteriaceae        <NA>    <NA>
## 813217 Enterobacteriales Enterobacteriaceae  Klebsiella    <NA>
##        Genus_Species
## 345362            NA
## 588216            NA
## 297057   Bacteroides
## 518002            NA
## 581782            NA
## 813217    Klebsiella
```

```
write.table(sig_cephre_tax,"differential_abundance/Supp_Data19.DESeq2_CephRE.txt", sep="\t")
```

Then during VRE colonization.

```
sample_data(LT_phylo)$VRE_sample = factor(sample_data(LT_phylo)$VRE_sample)
dds = phyloseq_to_deseq2(LT_phylo, ~VRE_sample)
geoMeans = apply(counts(dds), 1, gm_mean) # Calculate geometric means
dds = estimateSizeFactors(dds, geoMeans = geoMeans)
dds = estimateDispersions(dds)
vst = getVarianceStabilizedData(dds)
deseq = DESeq(dds,fitType="local") # Run DESeq2
# resultsNames(deseq) # Should show the comparison of interest (1_vs_0)
res_vre = results(deseq)
res_vre = res_vre[order(res_vre$padj, na.last=NA),]
res_vre_tax = cbind(as(res_vre, "data.frame"),
                    as(tax_table(LT_phylo)[rownames(res_vre), ],
                       "matrix")) # Merge in taxonomic information
alpha = 0.05 # Significant cut-off for FDR-adjusted p-value
sig_vre_tax = res_vre_tax[(res_vre_tax$padj<alpha),]
head(sig_vre_tax)
```

```
##         baseMean log2FoldChange     lfcSE      stat       pvalue
## 310608  70.66786      -7.607777 0.5088217 -14.95175 1.516989e-50
## 361722 300.43802      -4.586736 0.3863482 -11.87202 1.654154e-32
## 522511 176.56291      -4.945791 0.4384889 -11.27917 1.663036e-29
## 175751  17.40364      -5.851040 0.5299905 -11.03990 2.453142e-28
## 347382 159.94380      -4.414656 0.4005223 -11.02225 2.985088e-28
## 456774  33.26326      -5.878549 0.5356994 -10.97359 5.119572e-28
##                padj  Kingdom     Phylum      Class         Order
## 310608 1.146843e-47 Bacteria Firmicutes Clostridia Clostridiales
## 361722 6.252701e-30 Bacteria Firmicutes Clostridia Clostridiales
## 522511 4.190850e-27 Bacteria Firmicutes Clostridia Clostridiales
## 175751 4.513453e-26 Bacteria Firmicutes Clostridia Clostridiales
## 347382 4.513453e-26 Bacteria Firmicutes Clostridia Clostridiales
## 456774 6.450661e-26 Bacteria Firmicutes Clostridia Clostridiales
##                  Family             Genus     Species
## 310608 Lachnospiraceae             Dorea        <NA>
## 361722 Ruminococcaceae Faecalibacterium prausnitzii
## 522511 Ruminococcaceae Faecalibacterium prausnitzii
## 175751 Lachnospiraceae              <NA>        <NA>
## 347382 Lachnospiraceae              <NA>        <NA>
## 456774 Lachnospiraceae      Coprococcus        <NA>
##                         Genus_Species
## 310608                          Dorea
## 361722 Faecalibacterium prausnitzii
```

```
## 522511 Faecalibacterium prausnitzii
## 175751                           NA
## 347382                           NA
## 456774                  Coprococcus
```

```
write.table(sig_vre_tax,"differential_abundance/Supp_Data21.DESeq2_VRE.txt", sep="\t")
```

Lastly, during colonization with any MDRO (CRE, Ceph-RE, and/or VRE)

```
sample_data(LT_phylo)$MDRO_sample = factor(sample_data(LT_phylo)$MDRO_sample)
dds = phyloseq_to_deseq2(LT_phylo, ~MDRO_sample)
geoMeans = apply(counts(dds), 1, gm_mean) # Calculate geometric means
dds = estimateSizeFactors(dds, geoMeans = geoMeans)
dds = estimateDispersions(dds)
vst = getVarianceStabilizedData(dds)
deseq = DESeq(dds,fitType="local") # Run DESeq2
# resultsNames(deseq) # Should show the comparison of interest (1_vs_0)
res_mdro = results(deseq)
res_mdro = res_mdro[order(res_mdro$padj, na.last=NA),]
res_mdro_tax = cbind(as(res_mdro, "data.frame"),
                     as(tax_table(LT_phylo)[rownames(res_mdro), ],
                        "matrix")) # Merge in taxonomic information
alpha = 0.05 # Significant cut-off for FDR-adjusted p-value
sig_mdro_tax = res_mdro_tax[(res_mdro_tax$padj<alpha),]
head(sig_mdro_tax)
```

```
##          baseMean log2FoldChange     lfcSE      stat       pvalue
## 590982 11292.4634       4.321021 0.3140973 13.756948 4.626476e-43
## 144814  1022.8058       3.435099 0.3265234 10.520224 6.970799e-26
## 345362  7723.4620       3.012619 0.2955962 10.191672 2.160162e-24
## 579608   418.7601       2.360265 0.2355521 10.020139 1.243276e-23
## 297057   257.2128      -3.771442 0.3953952 -9.538413 1.450347e-21
## 588216  4147.2274       2.603640 0.2761719  9.427606 4.195555e-21
##                padj  Kingdom         Phylum               Class
## 590982 3.497616e-40 Bacteria     Firmicutes             Bacilli
## 144814 2.634962e-23 Bacteria Proteobacteria Gammaproteobacteria
## 345362 5.443609e-22 Bacteria Proteobacteria Gammaproteobacteria
## 579608 2.349791e-21 Bacteria     Firmicutes             Bacilli
## 297057 2.192925e-19 Bacteria  Bacteroidetes         Bacteroidia
## 588216 5.286399e-19 Bacteria Proteobacteria Gammaproteobacteria
##                      Order             Family         Genus Species
## 590982   Lactobacillales    Enterococcaceae  Enterococcus    <NA>
## 144814 Enterobacteriales Enterobacteriaceae          <NA>    <NA>
## 345362 Enterobacteriales Enterobacteriaceae          <NA>    <NA>
## 579608   Lactobacillales   Streptococcaceae Streptococcus    <NA>
## 297057     Bacteroidales     Bacteroidaceae   Bacteroides    <NA>
## 588216 Enterobacteriales Enterobacteriaceae          <NA>    <NA>
##        Genus_Species
## 590982  Enterococcus
## 144814            NA
## 345362            NA
## 579608 Streptococcus
## 297057   Bacteroides
## 588216            NA
```

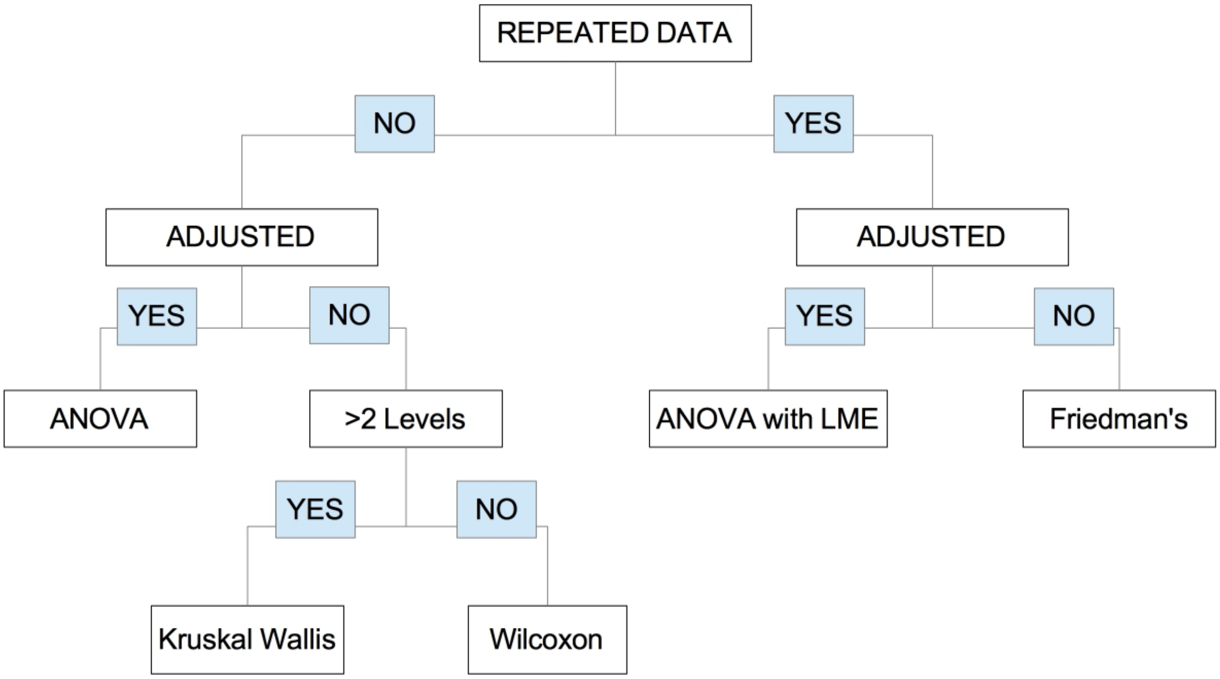

Figure 1: ANCOM flowchart

```
write.table(sig_mdro_tax,"differential_abundance/Supp_Data23.DESeq2_MDRO.txt", sep="\t")
```

`ANCOM`

Due to the potential for high false-positive rates using `DESeq2` (Weiss et al., *Microbiome* **2017**), we also performed Analysis of Composition of Microbiomes (`ANCOM`) to refine our identification of bacterial taxa enriched in selected groups (Mandal et al., *Microb Ecol Health Dis* **2015**).

To figure out the appropriate parameters for our ANCOM analysis, we can look at the flowchart provided in the `ANCOM` manual. Big shout-out to my co-author Angela Gomez-Simmonds for repeatedly walking through this decision tree with me!

Below are the same analyses as shown for `DESeq2`, to highlight the difference between these methodologies.

Supplementary Data 4. Alcohol-related liver disease (ARLD) and pre-LT microbiota

Referring to the flowchart above, we know that since we are only looking at pre-LT samples this subset is not repeated data (only 1 sample per patient). Also, we first ran this analysis without adjusting for any covariates, as with the `DESeq2` above.

```
otus = t(data.frame(otu_table(LT_phylo))) #transposed OTU table (rows are samples)
rownames(otus) = substr(rownames(otus), start=2, stop=6)
```

```r
otus = data.frame(Sample.ID=rownames(otus), otus)

metadata = data.frame(sample_data(LT_phylo))
metadata$Sample.ID = metadata$StoolID
taxa <- data.frame(tax_table(LT_phylo))
taxa$OTUs = rownames(taxa)


metadata_preLT = metadata[which(metadata$Time_Category2=="a-Pre"),]
otus_preLT = otus[which(otus$Sample.ID %in% metadata_preLT$StoolID),]
metadata_preLT$Sample.ID = metadata_preLT$StoolID


comparison_test=ANCOM.main(OTUdat=otus_preLT,
                           Vardat=metadata_preLT,
                           adjusted=F, # Not adjusting for anything
                           repeated=F, # Not repeated data
                           main.var="ARLD",
                           adj.formula=NULL, # Not adjusting for anything
                           repeat.var=NULL, # Not repeated data
                           longitudinal=FALSE, # Just 1 timepoint
                           random.formula=NULL,
                           multcorr=2, # Taxa-based p-value correction (recommended)
                           sig=0.05, # $\alpha$ = 0.05
                           prev.cut=0.90) # Remove taxa absent from >=90% of samples

W.taxa.arld = comparison_test$W.taxa
W.taxa.arld$OTUs = substring(W.taxa.arld$otu.names,2)
W.taxa.arld.sig <- W.taxa.arld[which(W.taxa.arld$detected_0.6=="TRUE"),]
taxa.sig <- taxa[which(taxa$OTUs %in% W.taxa.arld.sig$OTUs),]
W.taxa.merged.arld <- merge(W.taxa.arld.sig, taxa, by="OTUs") #Merge results with taxonomic info

head(W.taxa.merged.arld)
```

```
##      OTUs otu.names W_stat detected_0.9 detected_0.8 detected_0.7
## 1  137580   X137580    480         TRUE         TRUE         TRUE
## 2  202131   X202131    488         TRUE         TRUE         TRUE
## 3  255367   X255367    368        FALSE        FALSE         TRUE
## 4  298120   X298120    411        FALSE        FALSE         TRUE
## 5  318320   X318320    402        FALSE        FALSE         TRUE
## 6 4480189  X4480189    481         TRUE         TRUE         TRUE
##   detected_0.6  Kingdom     Phylum   Class           Order
## 1         TRUE Bacteria Firmicutes Bacilli Lactobacillales
## 2         TRUE Bacteria Firmicutes Bacilli Lactobacillales
## 3         TRUE Bacteria Firmicutes Bacilli Lactobacillales
## 4         TRUE Bacteria Firmicutes Bacilli Lactobacillales
## 5         TRUE Bacteria Firmicutes Bacilli Lactobacillales
## 6         TRUE Bacteria Firmicutes Bacilli Lactobacillales
##             Family         Genus Species      Genus_Species
## 1 Lactobacillaceae Lactobacillus    <NA>      Lactobacillus
## 2 Lactobacillaceae Lactobacillus    <NA>      Lactobacillus
## 3 Lactobacillaceae Lactobacillus    <NA>      Lactobacillus
## 4 Leuconostocaceae          <NA>    <NA>                 NA
## 5 Lactobacillaceae Lactobacillus    <NA>      Lactobacillus
```

```
## 6 Lactobacillaceae Lactobacillus    zeae Lactobacillus zeae
```

```
write.table(W.taxa.merged.arld, "differential_abundance/Supp_Data4.ANCOM_ARLD_preLT.txt", sep="\t")
```

Multiple *Lactobacillus* spp. (including *L. zeae*) and *Streptococcus* spp. OTUs were differentially abundant in the ARLD group. This mostly aligns with the most differentially abundant OTUs identified by DESeq2.

Supplementary Data 6. MELD at LT and pre-LT microbiota

```
metadata_preLT$MELD2016_cat = factor(metadata_preLT$MELD2016_cat, levels=c("LOW","HIGH"))

comparison_test=ANCOM.main(OTUdat=otus_preLT,
                           Vardat=metadata_preLT,
                           adjusted=F, # Not adjusting for anything
                           repeated=F, # Not repeated data
                           main.var="MELD2016_cat",
                           adj.formula=NULL, # Not adjusting for anything
                           repeat.var=NULL, # Not repeated data
                           longitudinal=FALSE, # Just 1 timepoint
                           random.formula=NULL,
                           multcorr=2, # Taxa-based p-value correction (recommended)
                           sig=0.05, # $\alpha$ = 0.05
                           prev.cut=0.90) # Remove taxa absent from >=90% of samples

W.taxa.meld = comparison_test$W.taxa
W.taxa.meld$OTUs = substring(W.taxa.meld$otu.names,2)
W.taxa.meld.sig <- W.taxa.meld[which(W.taxa.meld$detected_0.6=="TRUE"),]
taxa.sig <- taxa[which(taxa$OTUs %in% W.taxa.meld.sig$OTUs),]
W.taxa.merged.meld <- merge(W.taxa.meld.sig, taxa, by="OTUs") #Merge results with taxonomic info

head(W.taxa.merged.meld)
```

```
##      OTUs otu.names W_stat detected_0.9 detected_0.8 detected_0.7
## 1 1076587  X1076587    463        FALSE         TRUE         TRUE
## 2  174493   X174493    458        FALSE         TRUE         TRUE
## 3  328105   X328105    480         TRUE         TRUE         TRUE
## 4  342380   X342380    499         TRUE         TRUE         TRUE
## 5  347382   X347382    390        FALSE        FALSE         TRUE
## 6  359158   X359158    322        FALSE        FALSE        FALSE
##   detected_0.6  Kingdom     Phylum      Class         Order
## 1         TRUE Bacteria Firmicutes Clostridia Clostridiales
## 2         TRUE Bacteria Firmicutes Clostridia Clostridiales
## 3         TRUE Bacteria Firmicutes Clostridia Clostridiales
## 4         TRUE Bacteria Firmicutes Clostridia Clostridiales
## 5         TRUE Bacteria Firmicutes Clostridia Clostridiales
## 6         TRUE Bacteria Firmicutes Clostridia Clostridiales
##             Family   Genus         Species         Genus_Species
## 1 Lachnospiraceae   Dorea formicigenerans Dorea formicigenerans
## 2 Ruminococcaceae    <NA>            <NA>                    NA
## 3 Lachnospiraceae    <NA>            <NA>                    NA
## 4 Lachnospiraceae Blautia            <NA>               Blautia
## 5 Lachnospiraceae    <NA>            <NA>                    NA
## 6 Lachnospiraceae    <NA>            <NA>                    NA
```

```
write.table(W.taxa.merged.meld, "differential_abundance/Supp_Data6.ANCOM_MELD_preLT.txt", sep="\t")
```

Supplementary Data 8. CTP class and pre-LT microbiota

```
metadata_preLT_CTP = metadata_preLT[(which(metadata_preLT$CTP_Class != "B")),]
metadata_preLT_CTP$CTP_Class = factor(metadata_preLT_CTP$CTP_Class, levels=c("A","C"))
otus_preLT_CTP = otus_preLT[(which(otus_preLT$Sample.ID %in% metadata_preLT_CTP$Sample.ID)),]


comparison_test=ANCOM.main(OTUdat=otus_preLT_CTP,
                           Vardat=metadata_preLT_CTP,
                           adjusted=F, # Not adjusting for anything
                           repeated=F, # Not repeated data
                           main.var="CTP_Class",
                           adj.formula=NULL, # Not adjusting for anything
                           repeat.var=NULL, # Not repeated data
                           longitudinal=FALSE, # Just 1 timepoint
                           random.formula=NULL,
                           multcorr=2, # Taxa-based p-value correction (recommended)
                           sig=0.05, # $\alpha$ = 0.05
                           prev.cut=0.90) # Remove taxa absent from >=90% of samples

W.taxa.ctp = comparison_test$W.taxa
W.taxa.ctp$OTUs = substring(W.taxa.ctp$otu.names,2)
W.taxa.ctp.sig <- W.taxa.ctp[which(W.taxa.ctp$detected_0.6=="TRUE"),]
taxa.sig <- taxa[which(taxa$OTUs %in% W.taxa.ctp.sig$OTUs),]
W.taxa.merged.ctp <- merge(W.taxa.ctp.sig, taxa, by="OTUs") #Merge results with taxonomic info

head(W.taxa.merged.ctp)
```

```
##      OTUs otu.names W_stat detected_0.9 detected_0.8 detected_0.7
## 1 1076587  X1076587    345        FALSE        FALSE        FALSE
## 2  174493   X174493    332        FALSE        FALSE        FALSE
## 3  213885   X213885    348        FALSE        FALSE        FALSE
## 4 2979308  X2979308    393        FALSE        FALSE         TRUE
## 5  309720   X309720    421        FALSE        FALSE         TRUE
## 6  313968   X313968    327        FALSE        FALSE        FALSE
##   detected_0.6  Kingdom     Phylum      Class          Order
## 1         TRUE Bacteria Firmicutes Clostridia Clostridiales
## 2         TRUE Bacteria Firmicutes Clostridia Clostridiales
## 3         TRUE Bacteria Firmicutes Clostridia Clostridiales
## 4         TRUE Bacteria Firmicutes Clostridia Clostridiales
## 5         TRUE Bacteria Firmicutes Clostridia Clostridiales
## 6         TRUE Bacteria Firmicutes Clostridia Clostridiales
##             Family        Genus         Species        Genus_Species
## 1 Lachnospiraceae        Dorea formicigenerans Dorea formicigenerans
## 2 Ruminococcaceae          <NA>            <NA>                   NA
## 3 Ruminococcaceae Oscillospira            <NA>         Oscillospira
## 4 Ruminococcaceae Ruminococcus            <NA>         Ruminococcus
## 5 Ruminococcaceae          <NA>            <NA>                   NA
## 6 Ruminococcaceae          <NA>            <NA>                   NA
```

```
write.table(W.taxa.merged.ctp, "differential_abundance/Supp_Data8.ANCOM_CTP_CvA_preLT.txt", sep="\t")
```

Supplementary Data 14. Longitudinal changes in microbiota stratified by primary disease etiology

Unlike the prior examples, we are now adjusting for liver disease etiology as the major covariate (`adjusted=T`, `adj.formula="Primary_Diagnosis"`). This also represents longitudinal data, so we set `longitudinal=T` in the `ANCOM.main` function. Since there are multiple timepoints, we set `repeated=T`, but `repeat.var` to `NULL` since we are running an adjusted analysis (`repeat.var` is used for the Friedman's test). The random effects of StudyID (repeated measurements per patient) are taken account in the linear mixed-effect (LME) testing using `random.formula="~1|StudyID"`.

```
comparison_test=ANCOM.main(OTUdat=otus,
                           Vardat=metadata,
                           adjusted=T,
                           repeated=T,
                           main.var="Time_Category2",
                           adj.formula="Primary_Diagnosis",
                           repeat.var=NULL, # see manual; only for Friedman's
                           longitudinal=T,
                           random.formula="~1|StudyID",
                           multcorr=2, # Taxa-based p-value correction (recommended)
                           sig=0.05, # $\alpha$ = 0.05
                           prev.cut=0.90) # Remove taxa absent from >=90% of samples

W.taxa.timecat = comparison_test$W.taxa
W.taxa.timecat$OTUs = substring(W.taxa.timecat$otu.names,2)
W.taxa.timecat.sig <- W.taxa.timecat[which(W.taxa.timecat$detected_0.6=="TRUE"),]
taxa.sig <- taxa[which(taxa$OTUs %in% W.taxa.timecat.sig$OTUs),]
W.taxa.merged.timecat <- merge(W.taxa.timecat.sig, taxa, by="OTUs") #Merge results with taxonomic info

head(W.taxa.merged.timecat)
```

```
##      OTUs otu.names W_stat detected_0.9 detected_0.8 detected_0.7
## 1 1017181  X1017181    442        FALSE         TRUE         TRUE
## 2  102222   X102222    383        FALSE        FALSE        FALSE
## 3 1066410  X1066410    423        FALSE        FALSE         TRUE
## 4 1076587  X1076587    475        FALSE         TRUE         TRUE
## 5 1089121  X1089121    471        FALSE         TRUE         TRUE
## 6 1111582  X1111582    403        FALSE        FALSE         TRUE
##   detected_0.6  Kingdom         Phylum          Class            Order
## 1         TRUE Bacteria Actinobacteria Actinobacteria Actinomycetales
## 2         TRUE Bacteria     Firmicutes        Bacilli Lactobacillales
## 3         TRUE Bacteria Actinobacteria Actinobacteria Actinomycetales
## 4         TRUE Bacteria     Firmicutes     Clostridia   Clostridiales
## 5         TRUE Bacteria Actinobacteria Actinobacteria Actinomycetales
## 6         TRUE Bacteria     Firmicutes        Bacilli Lactobacillales
##             Family        Genus         Species            Genus_Species
## 1   Micrococcaceae       Rothia    mucilaginosa      Rothia mucilaginosa
## 2 Lactobacillaceae  Pediococcus    acidilactici Pediococcus acidilactici
## 3 Actinomycetaceae  Actinomyces            <NA>              Actinomyces
## 4  Lachnospiraceae        Dorea formicigenerans    Dorea formicigenerans
## 5 Actinomycetaceae  Actinomyces            <NA>              Actinomyces
## 6  Enterococcaceae Enterococcus            <NA>             Enterococcus
```

```
write.table(W.taxa.merged.timecat, "differential_abundance/Supp_Data14.ANCOM_timecategory.txt", sep="\t
```

Supplementary Data 16. Pre-LT microbiota differentially abundant in patients who develop MDRO colonization

```
metadata_preLT$MDRO_within1yr = factor(metadata_preLT$MDRO_within1yr)

comparison_test=ANCOM.main(OTUdat=otus_preLT,
                           Vardat=metadata_preLT,
                           adjusted=F, # Not adjusting for anything
                           repeated=F, # Not repeated data
                           main.var="MDRO_within1yr",
                           adj.formula=NULL, # Not adjusting for anything
                           repeat.var=NULL, # Not repeated data
                           longitudinal=FALSE, # Just 1 timepoint
                           random.formula=NULL,
                           multcorr=2, # Taxa-based p-value correction (recommended)
                           sig=0.05, # $\alpha$ = 0.05
                           prev.cut=0.90) # Remove taxa absent from >=90% of samples

W.taxa.mdro_1yr = comparison_test$W.taxa
W.taxa.mdro_1yr$OTUs = substring(W.taxa.mdro_1yr$otu.names,2)
W.taxa.mdro_1yr.sig <- W.taxa.mdro_1yr[which(W.taxa.mdro_1yr$detected_0.6=="TRUE"),]
taxa.sig <- taxa[which(taxa$OTUs %in% W.taxa.mdro_1yr.sig$OTUs),]
W.taxa.merged.mdro_1yr <- merge(W.taxa.mdro_1yr.sig, taxa, by="OTUs") #Merge results with taxonomic inf

head(W.taxa.merged.mdro_1yr)
```

```
##      OTUs otu.names W_stat detected_0.9 detected_0.8 detected_0.7
## 1 1076587  X1076587    368        FALSE        FALSE         TRUE
## 2  331850   X331850    350        FALSE        FALSE        FALSE
## 3  342380   X342380    408        FALSE        FALSE         TRUE
## 4  364824   X364824    449        FALSE         TRUE         TRUE
## 5  590982   X590982    385        FALSE        FALSE         TRUE
##   detected_0.6  Kingdom     Phylum      Class           Order
## 1         TRUE Bacteria Firmicutes Clostridia   Clostridiales
## 2         TRUE Bacteria Firmicutes Clostridia   Clostridiales
## 3         TRUE Bacteria Firmicutes Clostridia   Clostridiales
## 4         TRUE Bacteria Firmicutes Clostridia   Clostridiales
## 5         TRUE Bacteria Firmicutes    Bacilli Lactobacillales
##            Family        Genus         Species         Genus_Species
## 1 Lachnospiraceae        Dorea formicigenerans Dorea formicigenerans
## 2 Lachnospiraceae      Blautia            <NA>               Blautia
## 3 Lachnospiraceae      Blautia            <NA>               Blautia
## 4 Lachnospiraceae      Blautia           obeum         Blautia obeum
## 5 Enterococcaceae Enterococcus            <NA>          Enterococcus
```

```
write.table(W.taxa.merged.mdro_1yr, "differential_abundance/Supp_Data16.ANCOM_MDRO_1yr_preLT.txt", sep=
```

Supplementary Data 18, 20, 22, and 24. Microbiota differentially abundant in patients with colonizing CRE, Ceph-RE, VRE, and any MDRO (all time-points)

```
metadata$CRE_sample = factor(metadata$CRE_sample)

comparison_test=ANCOM.main(OTUdat=otus,
                           Vardat=metadata,
                           adjusted=T,
                           repeated=F,
                           main.var="CRE_sample",
```

```
                            adj.formula="Time_Category2",
                            repeat.var=NULL,
                            longitudinal=F,
                            random.formula="~1|StudyID",
                            multcorr=2,
                            sig=0.05,
                            prev.cut=0.90)

W.taxa.cre = comparison_test$W.taxa
W.taxa.cre$OTUs = substring(W.taxa.cre$otu.names,2)
W.taxa.cre.sig <- W.taxa.cre[which(W.taxa.cre$detected_0.6=="TRUE"),]
taxa.sig <- taxa[which(taxa$OTUs %in% W.taxa.cre.sig$OTUs),]
W.taxa.merged.cre <- merge(W.taxa.cre.sig, taxa, by="OTUs") #Merge results with taxonomic info

head(W.taxa.merged.cre)
```

```
##     OTUs otu.names W_stat detected_0.9 detected_0.8 detected_0.7
## 1 144814   X144814    543         TRUE         TRUE         TRUE
## 2 145801   X145801    352        FALSE        FALSE        FALSE
## 3 183162   X183162    349        FALSE        FALSE        FALSE
## 4 197286   X197286    524         TRUE         TRUE         TRUE
## 5 203579   X203579    432        FALSE        FALSE         TRUE
## 6 210269   X210269    476        FALSE         TRUE         TRUE
##   detected_0.6  Kingdom         Phylum               Class
## 1         TRUE Bacteria Proteobacteria Gammaproteobacteria
## 2         TRUE Bacteria     Firmicutes     Erysipelotrichi
## 3         TRUE Bacteria     Firmicutes          Clostridia
## 4         TRUE Bacteria Proteobacteria Gammaproteobacteria
## 5         TRUE Bacteria Proteobacteria Gammaproteobacteria
## 6         TRUE Bacteria Proteobacteria Gammaproteobacteria
##                Order              Family        Genus Species
## 1  Enterobacteriales  Enterobacteriaceae         <NA>    <NA>
## 2 Erysipelotrichales Erysipelotrichaceae         <NA>    <NA>
## 3      Clostridiales     Ruminococcaceae Oscillospira    <NA>
## 4  Enterobacteriales  Enterobacteriaceae         <NA>    <NA>
## 5  Enterobacteriales  Enterobacteriaceae         <NA>    <NA>
## 6  Enterobacteriales  Enterobacteriaceae Enterobacter cloacae
##          Genus_Species
## 1                   NA
## 2                   NA
## 3         Oscillospira
## 4                   NA
## 5                   NA
## 6 Enterobacter cloacae
```

```
write.table(W.taxa.merged.cre, "differential_abundance/Supp_Data18.ANCOM_CRE.txt", sep="\t")
```

```
metadata$ESBL_sample = factor(metadata$ESBL_sample)

comparison_test=ANCOM.main(OTUdat=otus,
                           Vardat=metadata,
                           adjusted=T,
                           repeated=F,
                           main.var="ESBL_sample",
```

```
                           adj.formula="Time_Category2",
                           repeat.var=NULL,
                           longitudinal=F,
                           random.formula="~1|StudyID",
                           multcorr=2,
                           sig=0.05,
                           prev.cut=0.90)

W.taxa.esbl = comparison_test$W.taxa
W.taxa.esbl$OTUs = substring(W.taxa.esbl$otu.names,2)
W.taxa.esbl.sig <- W.taxa.esbl[which(W.taxa.esbl$detected_0.6=="TRUE"),]
taxa.sig <- taxa[which(taxa$OTUs %in% W.taxa.esbl.sig$OTUs),]
W.taxa.merged.esbl <- merge(W.taxa.esbl.sig, taxa, by="OTUs") #Merge results with taxonomic info

head(W.taxa.merged.esbl)
```

```
##      OTUs otu.names W_stat detected_0.9 detected_0.8 detected_0.7
## 1 1076587  X1076587    410        FALSE        FALSE         TRUE
## 2  138389   X138389    431        FALSE        FALSE         TRUE
## 3  144814   X144814    542         TRUE         TRUE         TRUE
## 4 1504042  X1504042    415        FALSE        FALSE         TRUE
## 5   15728    X15728    445        FALSE         TRUE         TRUE
## 6  167950   X167950    331        FALSE        FALSE        FALSE
##   detected_0.6  Kingdom         Phylum               Class
## 1         TRUE Bacteria     Firmicutes          Clostridia
## 2         TRUE Bacteria     Firmicutes             Bacilli
## 3         TRUE Bacteria Proteobacteria Gammaproteobacteria
## 4         TRUE Bacteria     Firmicutes          Clostridia
## 5         TRUE Bacteria     Firmicutes     Erysipelotrichi
## 6         TRUE Bacteria Proteobacteria Gammaproteobacteria
##                 Order                Family        Genus         Species
## 1      Clostridiales     Lachnospiraceae        Dorea formicigenerans
## 2    Lactobacillales     Enterococcaceae         <NA>            <NA>
## 3  Enterobacteriales  Enterobacteriaceae         <NA>            <NA>
## 4      Clostridiales     Ruminococcaceae Oscillospira            <NA>
## 5 Erysipelotrichales Erysipelotrichaceae    Holdemania            <NA>
## 6  Enterobacteriales  Enterobacteriaceae   Citrobacter            <NA>
##           Genus_Species
## 1 Dorea formicigenerans
## 2                    NA
## 3                    NA
## 4          Oscillospira
## 5            Holdemania
## 6           Citrobacter
```

```
write.table(W.taxa.merged.esbl, "differential_abundance/Supp_Data20.ANCOM_CephRE.txt", sep="\t")
```

```
metadata$VRE_sample = factor(metadata$VRE_sample)

comparison_test=ANCOM.main(OTUdat=otus,
                           Vardat=metadata,
                           adjusted=T,
                           repeated=F,
                           main.var="VRE_sample",
```

```
                              adj.formula="Time_Category2",
                              repeat.var=NULL,
                              longitudinal=F,
                              random.formula="~1|StudyID",
                              multcorr=2,
                              sig=0.05,
                              prev.cut=0.90)

W.taxa.vre = comparison_test$W.taxa
W.taxa.vre$OTUs = substring(W.taxa.vre$otu.names,2)
W.taxa.vre.sig <- W.taxa.vre[which(W.taxa.vre$detected_0.6=="TRUE"),]
taxa.sig <- taxa[which(taxa$OTUs %in% W.taxa.vre.sig$OTUs),]
W.taxa.merged.vre <- merge(W.taxa.vre.sig, taxa, by="OTUs") #Merge results with taxonomic info

head(W.taxa.merged.vre)
```

```
##      OTUs otu.names W_stat detected_0.9 detected_0.8 detected_0.7
## 1  102222   X102222    450        FALSE         TRUE         TRUE
## 2 1066410  X1066410    366        FALSE        FALSE        FALSE
## 3 1076587  X1076587    438        FALSE        FALSE         TRUE
## 4 1111582  X1111582    522         TRUE         TRUE         TRUE
## 5 1131894  X1131894    415        FALSE        FALSE         TRUE
## 6 1143045  X1143045    335        FALSE        FALSE        FALSE
##   detected_0.6  Kingdom         Phylum          Class            Order
## 1         TRUE Bacteria     Firmicutes        Bacilli Lactobacillales
## 2         TRUE Bacteria Actinobacteria Actinobacteria Actinomycetales
## 3         TRUE Bacteria     Firmicutes     Clostridia   Clostridiales
## 4         TRUE Bacteria     Firmicutes        Bacilli Lactobacillales
## 5         TRUE Bacteria  Cyanobacteria    Chloroplast    Streptophyta
## 6         TRUE Bacteria     Firmicutes     Clostridia   Clostridiales
##               Family        Genus          Species            Genus_Species
## 1 Lactobacillaceae  Pediococcus    acidilactici Pediococcus acidilactici
## 2 Actinomycetaceae  Actinomyces            <NA>              Actinomyces
## 3  Lachnospiraceae        Dorea formicigenerans    Dorea formicigenerans
## 4  Enterococcaceae Enterococcus            <NA>             Enterococcus
## 5             <NA>         <NA>            <NA>                       NA
## 6  Veillonellaceae  Veillonella          dispar       Veillonella dispar
```

```
write.table(W.taxa.merged.vre, "differential_abundance/Supp_Data22.ANCOM_VRE.txt", sep="\t")
```

```
metadata$MDRO_sample = factor(metadata$MDRO_sample)

comparison_test=ANCOM.main(OTUdat=otus,
                              Vardat=metadata,
                              adjusted=T,
                              repeated=F,
                              main.var="MDRO_sample",
                              adj.formula="Time_Category2",
                              repeat.var=NULL,
                              longitudinal=F,
                              random.formula="~1|StudyID",
                              multcorr=2,
                              sig=0.05,
                              prev.cut=0.90)
```

```r
W.taxa.mdro = comparison_test$W.taxa
W.taxa.mdro$OTUs = substring(W.taxa.mdro$otu.names,2)
W.taxa.mdro.sig <- W.taxa.mdro[which(W.taxa.mdro$detected_0.6=="TRUE"),]
taxa.sig <- taxa[which(taxa$OTUs %in% W.taxa.mdro.sig$OTUs),]
W.taxa.merged.mdro <- merge(W.taxa.mdro.sig, taxa, by="OTUs") #Merge results with taxonomic info

head(W.taxa.merged.mdro)
```

```
##      OTUs otu.names W_stat detected_0.9 detected_0.8 detected_0.7
## 1  102222   X102222    352        FALSE        FALSE        FALSE
## 2 1076587  X1076587    474        FALSE         TRUE         TRUE
## 3 1111582  X1111582    502         TRUE         TRUE         TRUE
## 4 1131894  X1131894    451        FALSE         TRUE         TRUE
## 5 1143045  X1143045    359        FALSE        FALSE        FALSE
## 6  138389   X138389    500         TRUE         TRUE         TRUE
##   detected_0.6  Kingdom        Phylum       Class           Order
## 1         TRUE Bacteria    Firmicutes     Bacilli Lactobacillales
## 2         TRUE Bacteria    Firmicutes  Clostridia   Clostridiales
## 3         TRUE Bacteria    Firmicutes     Bacilli Lactobacillales
## 4         TRUE Bacteria Cyanobacteria Chloroplast    Streptophyta
## 5         TRUE Bacteria    Firmicutes  Clostridia   Clostridiales
## 6         TRUE Bacteria    Firmicutes     Bacilli Lactobacillales
##             Family        Genus         Species            Genus_Species
## 1 Lactobacillaceae  Pediococcus    acidilactici Pediococcus acidilactici
## 2  Lachnospiraceae        Dorea formicigenerans    Dorea formicigenerans
## 3  Enterococcaceae Enterococcus            <NA>             Enterococcus
## 4             <NA>         <NA>            <NA>                       NA
## 5  Veillonellaceae  Veillonella          dispar       Veillonella dispar
## 6  Enterococcaceae         <NA>            <NA>                       NA
```

```r
write.table(W.taxa.merged.mdro, "differential_abundance/Supp_Data24.ANCOM_MDRO.txt", sep="\t")
```
