# Supplementary material for: Colonizing multidrug-resistant bacteria and the longitudinal evolution of the intestinal microbiome after liver transplantation
Source: Nat Commun. 2019 Oct 17;10:4715. doi: 10.1038/s41467-019-12633-4 (PMC6797753; doi:10.1038/s41467-019-12633-4)

Liver transplant microbiome and MDRO

Medini K. Annavajhala

July 19, 2019

Generating Figure 1: Liver transplant microbiome community (β-diversity) and MDRO

This R Markdown document is part of a series used to analyze data and generate figures for the citation below. The current document includes all code and options used to generate Figure 1 from the manuscript, which looks at pre- and post-liver transplant β-diversity and colonization by multidrug-resistant organisms (MDRO), defined as culture-positive for Enterobacteriaceae resistant to third-generation cephalosporins and beta lactam/beta lactamase inhibitor combinations (Ceph-RE), carbapenem-resistant Enterobacteriaceae (CRE), and/or vancomycin-resistant enterococci.

Title:

Colonizing multidrug-resistant organisms and the longitudinal evolution of the intestinal microbiome after liver transplantation

Authors:

Medini K. Annavajhala, Angela Gomez-Simmonds, Nenad Macesic, Sean B. Sullivan, Anna Kress, Sabrina D. Khan, Marla J. Giddins, Stephania Stump, Grace I. Kim, Ryan Narain, Elizabeth C. Verna, Anne-Catrin Uhlemann

Journal:

Nature Communications **2019**

Load Required Libraries:

```
library("phyloseq"); packageVersion("phyloseq")
```

```
## [1] '1.28.0'
```

```
library("vegan"); packageVersion("vegan")
```

```
## [1] '2.5.5'
```

```
library("grid"); packageVersion("grid")
```

```
## [1] '3.6.1'
```

```
library("gridExtra"); packageVersion("gridExtra")
```

```
## [1] '2.3'
```

```
library("data.table"); packageVersion("data.table")
```

```
## [1] '1.12.2'
```

```
library("ggplot2"); packageVersion("ggplot2")
```

```
## [1] '3.2.1'
```

```r
library("ggrepel"); packageVersion("ggrepel")
```

```
## [1] '0.8.1'
```

```r
library("egg"); packageVersion("egg")
```

```
## [1] '0.4.5'
```

Generate Figure 1

Import phyloseq object and metadata

```r
LT_data <- read.table("inputs/Fig1_metadata.txt", header=T)
colnames(LT_data)
```

```
## [1] "StoolID"        "ESBL_sample"    "CRE_sample"     "VRE_sample"
## [5] "MDRO_sample"    "MDRO_within1yr"
```

```r
# StoolID : sample ID
#
# ESBL_sample: binary (0=no; 1=yes) variable indicating whether the stool sample was
#              culture-positive for Enterobacteriaceae resistant to third-generation
#              cephalosporins and beta lactam/beta lactamase inhibitor combinations
#              (Ceph-RE), sometimes referred to as ESBLs (extended-spectrum beta-lactam
#              producers)
#
# CRE_sample: binary (0=no; 1=yes) variable indicating whether the stool sample was
#             culture-positive for carbapenem-resistant Enterobacteriaceae (CRE)
#
# VRE_sample: binary (0=no; 1=yes) variable indicating whether the stool sample was
#             culture-positive for vancomycin-resistant enterococci (VRE)
#
# MDRO_sample: binary (0=no; 1=yes) variable indicating whether the stool sample was
#             culture-positive for CRE, VRE, and/or Ceph-RE
#
# ESBL_within1yr: binary (0=no; 1=yes) variable indicating whether the patient associated
#                 with the sample was culture-positive for Ceph-RE at any point up to 1-yr
#                 post-LT
#
# CREL_within1yr: binary (0=no; 1=yes) variable indicating whether the patient associated
#                 with the sample was culture-positive for CRE at any point up to 1-yr
#                 post-LT
#
# VRE_within1yr: binary (0=no; 1=yes) variable indicating whether the patient associated
#                 with the sample was culture-positive for VRE at any point up to 1-yr
#                 post-LT
#
# MDRO_within1yr: binary (0=no; 1=yes) variable indicating whether the patient associated
#                 with the sample was culture-positive for CRE, VRE, and/or Ceph-RE at any
#                 point up to 1-yr post-LT
```

```r
phylo_relabun_filtered = readRDS("inputs/phylo_relabun_filtered.RDS")

LT_relabun = prune_samples(sample_names(phylo_relabun_filtered) %in%
                               LT_data$StoolID, phylo_relabun_filtered)
```

```
df1 <- LT_data[,1:6] #MDRO-related metadata
df2 <- data.frame(sample_data(LT_relabun),
                  "StoolID"=rownames(sample_data(LT_relabun))) #Sample names
merged <- merge(df2, df1, by="StoolID") #Merge preLT metadata with phyloseq object
merged <- sample_data(merged)
sample_names(merged) = merged$StoolID #fix names
sample_data(LT_relabun) <- sample_data(merged)

LT_relabun
```

```
## phyloseq-class experiment-level object
## otu_table()   OTU Table:         [ 878 taxa and 703 samples ]
## sample_data() Sample Data:       [ 703 samples by 7 sample variables ]
## tax_table()   Taxonomy Table:    [ 878 taxa by 8 taxonomic ranks ]
## phy_tree()    Phylogenetic Tree: [ 878 tips and 877 internal nodes ]
```

Ordinate and compare β-diversity

```
set.seed(2)

DistUF <- UniFrac(LT_relabun, parallel = T) # Calculate UniFrac distances
uf.nmds.ord <- ordinate(LT_relabun, "NMDS", DistUF) # UniFrac ordination

# We extract NMDS coordinates for each sample and merge in MDRO metadata
bdiv <- scores(uf.nmds.ord,display=c("sites"))
bdiv <- data.frame(bdiv)
sample_data(LT_relabun)[,3:7] <- lapply(sample_data(LT_relabun)[,3:7], factor)
metadata <- data.frame(sample_data(LT_relabun))
bdiv <- merge(bdiv, metadata, by="row.names")
xmin = min(bdiv$NMDS1)
ymin = min(bdiv$NMDS2)
xmax = max(bdiv$NMDS1) + 0.1
ymax = max(bdiv$NMDS2) + 0.05

# Run the PERMANOVA, extract P and F-statistic for annotation of plots

mdro_perm <- adonis(DistUF ~ MDRO_sample, data=metadata) #P=0.001, F=18.514
esbl_perm <- adonis(DistUF ~ ESBL_sample, data=metadata) #P=0.001, F=8.7764
cre_perm <- adonis(DistUF ~ CRE_sample, data=metadata)  #P=0.001, F=5.4426
vre_perm <- adonis(DistUF ~ VRE_sample, data=metadata)  #P=0.001, F=17.276
```

Plot panels A-D using NMDS coordinates

```
mdro_perm.F = as.character(paste("F=",
                                 round(mdro_perm$aov.tab$F.Model[1], 3),
                                 sep=""))
mdro_perm.P = as.character(paste("P=",
                                 round(mdro_perm$aov.tab$`Pr(>F)`[1], 3),
                                 sep=""))

p1_a <- ggplot() +
  geom_point(data=bdiv,aes(x=NMDS1, y=NMDS2, color=MDRO_sample), size=1.5) +
  # stat_ellipse(data=bdiv,aes(x=NMDS1, y=NMDS2, color=MDRO_sample),level=0.95,
  #              type = "t", linetype = 3, size=0.8, inherit.aes = TRUE)  +
```

```
  scale_color_manual(values= c("#f4eacd","#ffbb00")) +
  labs(color="MDRO\nColonization") +
  xlim(xmin,xmax) +
  ylim(ymin,ymax) +
  annotate(geom="text",x=xmax-0.01, y=ymin+0.05, label=mdro_perm.F, hjust=1) +
  annotate(geom="text",x=xmax-0.01, y=ymin+0.005, label=mdro_perm.P, hjust=1) +
  theme_classic() +
  theme(legend.text = element_text(size=10), legend.title = element_text(size=12))

p1_a
```

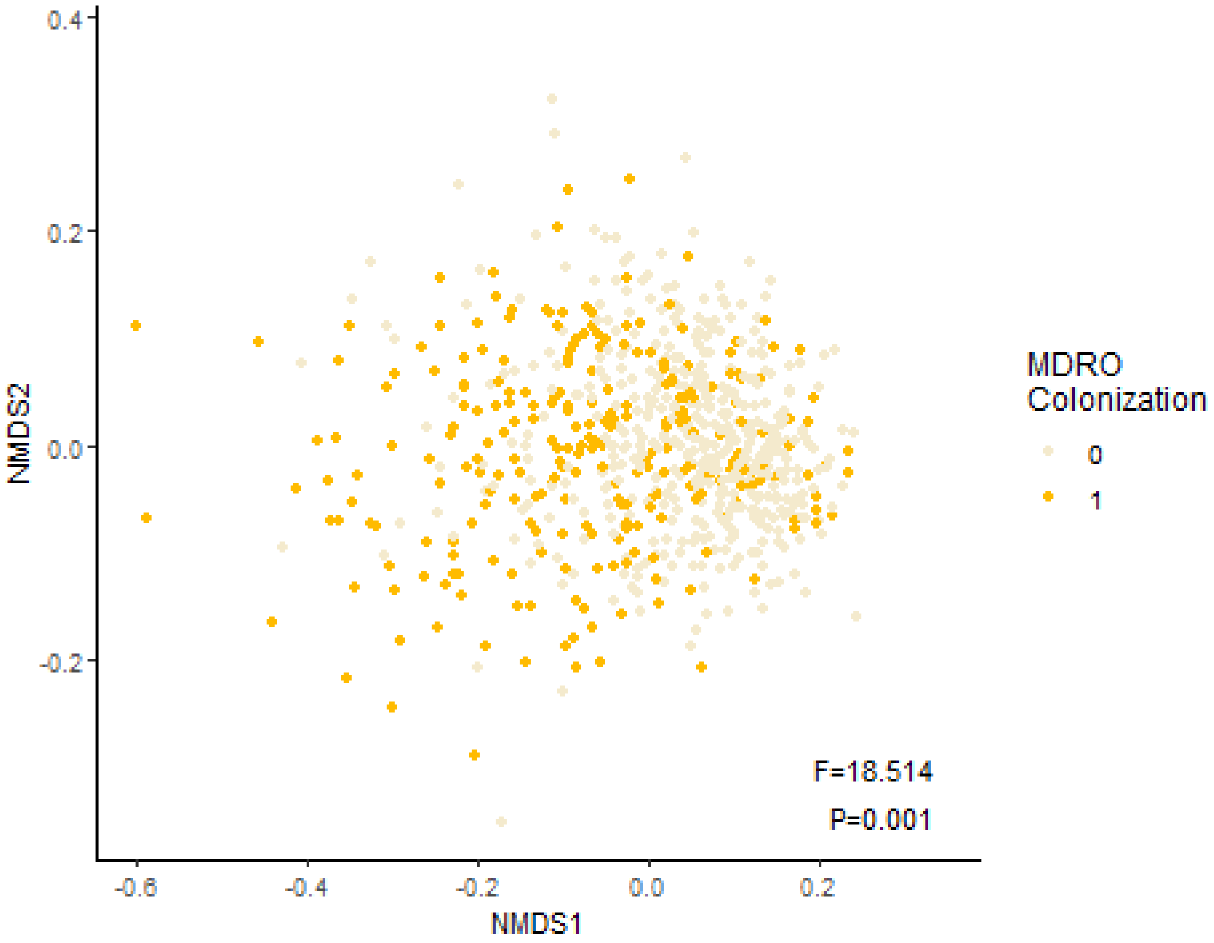


```
esbl_perm.F = as.character(paste("F=",
                                 round(esbl_perm$aov.tab$F.Model[1], 3),
                                 sep=""))
esbl_perm.P = as.character(paste("P=",
                                 round(esbl_perm$aov.tab$`Pr(>F)`[1], 3),
                                 sep=""))

p1_b <- ggplot() +
  geom_point(data=bdiv,aes(x=NMDS1, y=NMDS2, color=ESBL_sample), size=1.5) +
  # stat_ellipse(data=bdiv, aes(x=NMDS1, y=NMDS2, color=ESBL_sample), level=0.95,
  #              type = "t", linetype = 3, size=0.8, inherit.aes = T)  +
  scale_color_manual(values= c("#cee0c9","#387c26")) +
```

```
  labs(color="Ceph-RE\nColonization") +
  xlim(xmin,xmax) +
  ylim(ymin,ymax) +
  annotate(geom="text",x=xmax-0.01, y=ymin+0.05,label=esbl_perm.F, hjust=1) +
  annotate(geom="text",x=xmax-0.01, y=ymin+0.005,label=esbl_perm.P, hjust=1) +
  theme_classic() +
  theme(legend.text = element_text(size=10), legend.title = element_text(size=12))

p1_b
```

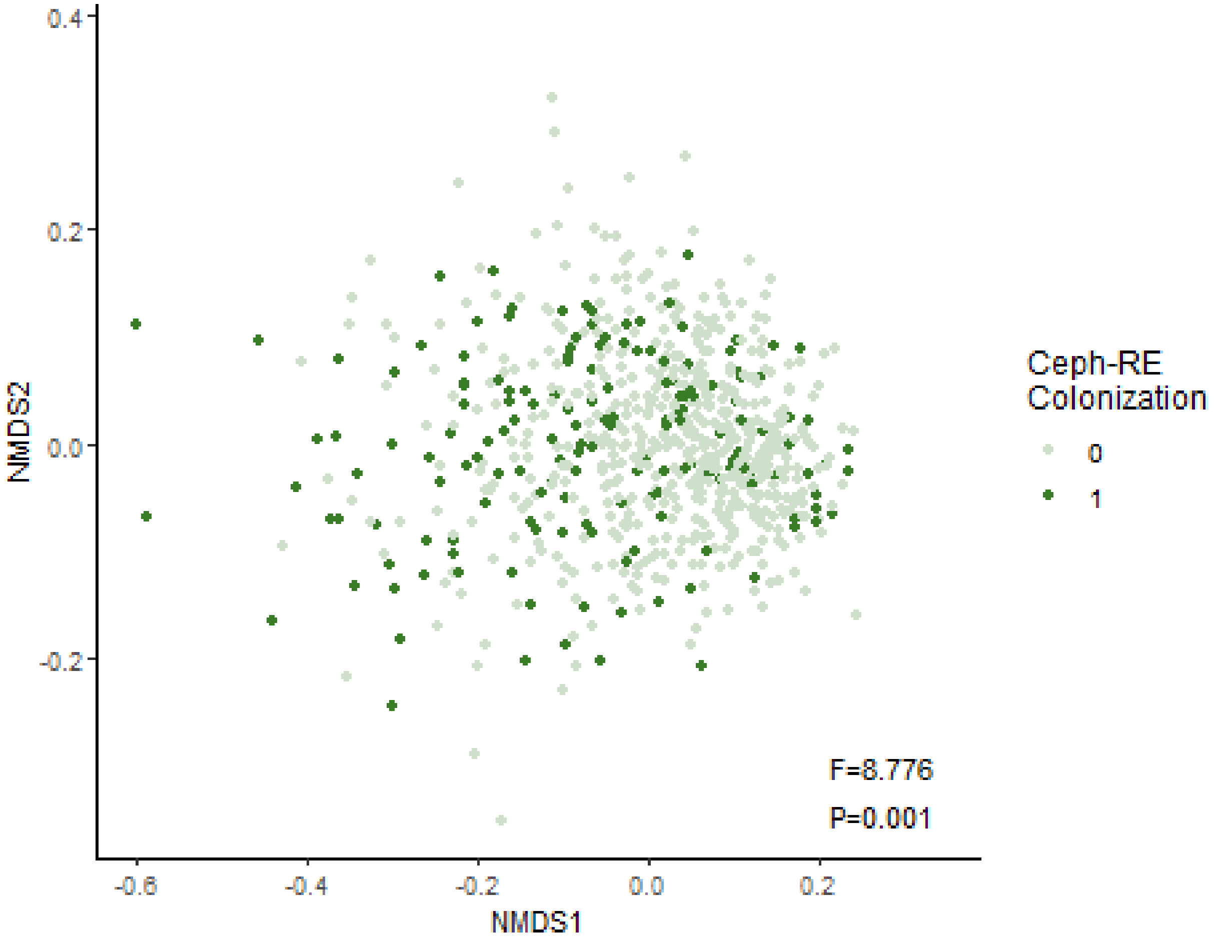


```
cre_perm.F = as.character(paste("F=",
                                round(cre_perm$aov.tab$F.Model[1], 3),
                                sep=""))
cre_perm.P = as.character(paste("P=",
                                round(cre_perm$aov.tab$`Pr(>F)`[1], 3),
                                sep=""))

p1_c <- ggplot() +
  geom_point(data=bdiv,aes(x=NMDS1, y=NMDS2, color=CRE_sample), size=1.5) +
  # stat_ellipse(data=bdiv, aes(x=NMDS1, y=NMDS2, color=CRE_sample), level=0.95,
  #              type = "t", linetype = 3, size=0.8, inherit.aes=T) +
  scale_color_manual(values= c("#e6dce8","#973aa5")) +
  labs(color="CRE\nColonization") +
```

```
  xlim(xmin,xmax) +
  ylim(ymin,ymax) +
  annotate(geom="text",x=xmax-0.01, y=ymin+0.05,label=cre_perm.F, hjust=1) +
  annotate(geom="text",x=xmax-0.01, y=ymin+0.005,label=cre_perm.P, hjust=1) +
  theme_classic() +
  theme(legend.text = element_text(size=10), legend.title = element_text(size=12))

p1_c
```

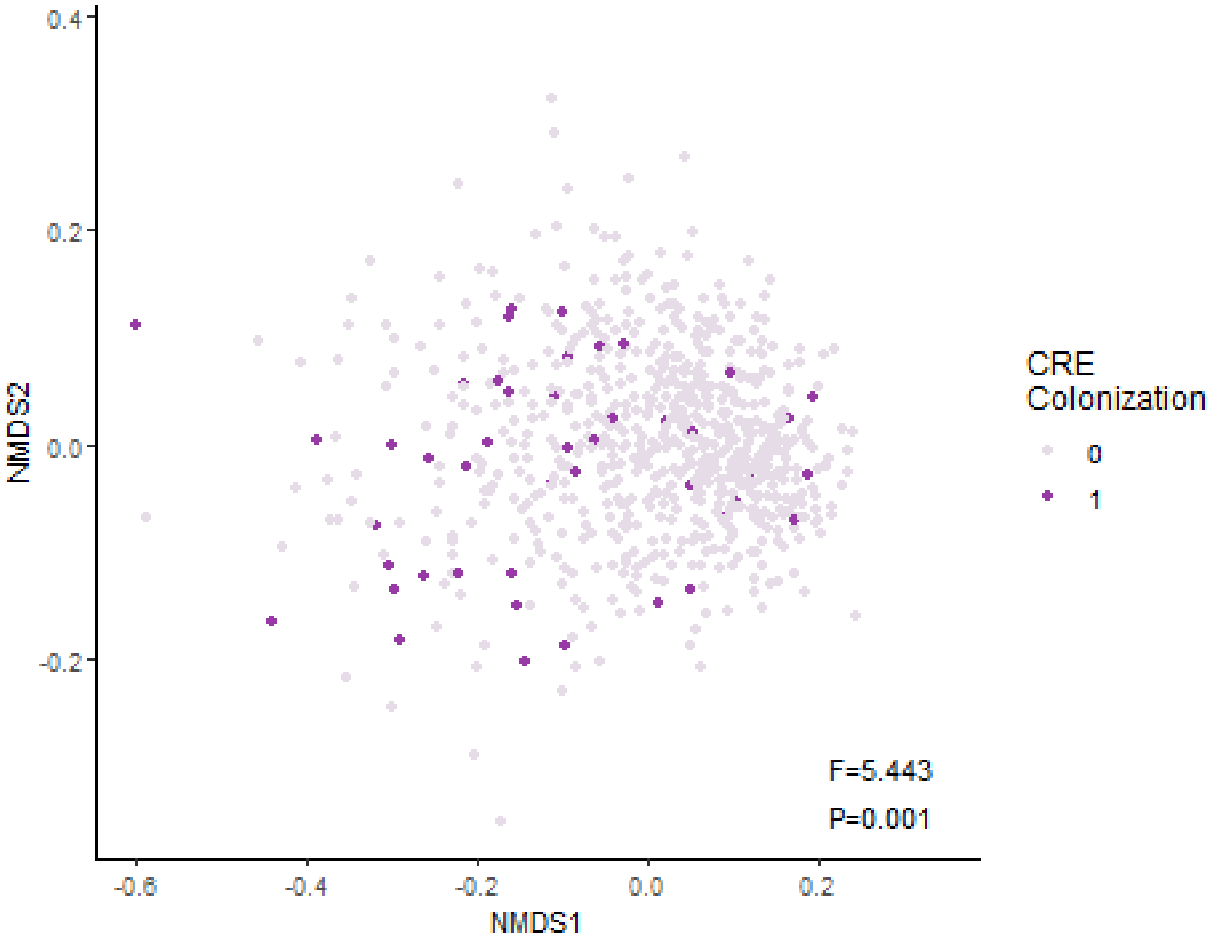


```
vre_perm.F = as.character(paste("F=",
                                  round(vre_perm$aov.tab$F.Model[1], 3),
                                  sep=""))
vre_perm.P = as.character(paste("P=",
                                  round(vre_perm$aov.tab$`Pr(>F)`[1], 3),
                                  sep=""))

p1_d <- ggplot() +
  geom_point(data=bdiv,aes(x=NMDS1, y=NMDS2, color=VRE_sample), size=1.5) +
  # stat_ellipse(data=bdiv, aes(x=NMDS1, y=NMDS2, color=VRE_sample),  level=0.95,
  #              type = "t", linetype = 3, size=0.8, inherit.aes = T) +
  scale_color_manual(values= c("#cebbc0","#c1244c")) +
  labs(color="VRE\nColonization") +
  xlim(xmin,xmax) +
```

```r
  ylim(ymin,ymax) +
  annotate(geom="text",x = xmax-0.01, y=ymin+0.05,label=vre_perm.F, hjust=1) +
  annotate(geom="text",x = xmax-0.01, y=ymin+0.005,label=vre_perm.P, hjust=1) +
  theme_classic() +
  theme(legend.text = element_text(size=10), legend.title = element_text(size=12))

p1_d
```

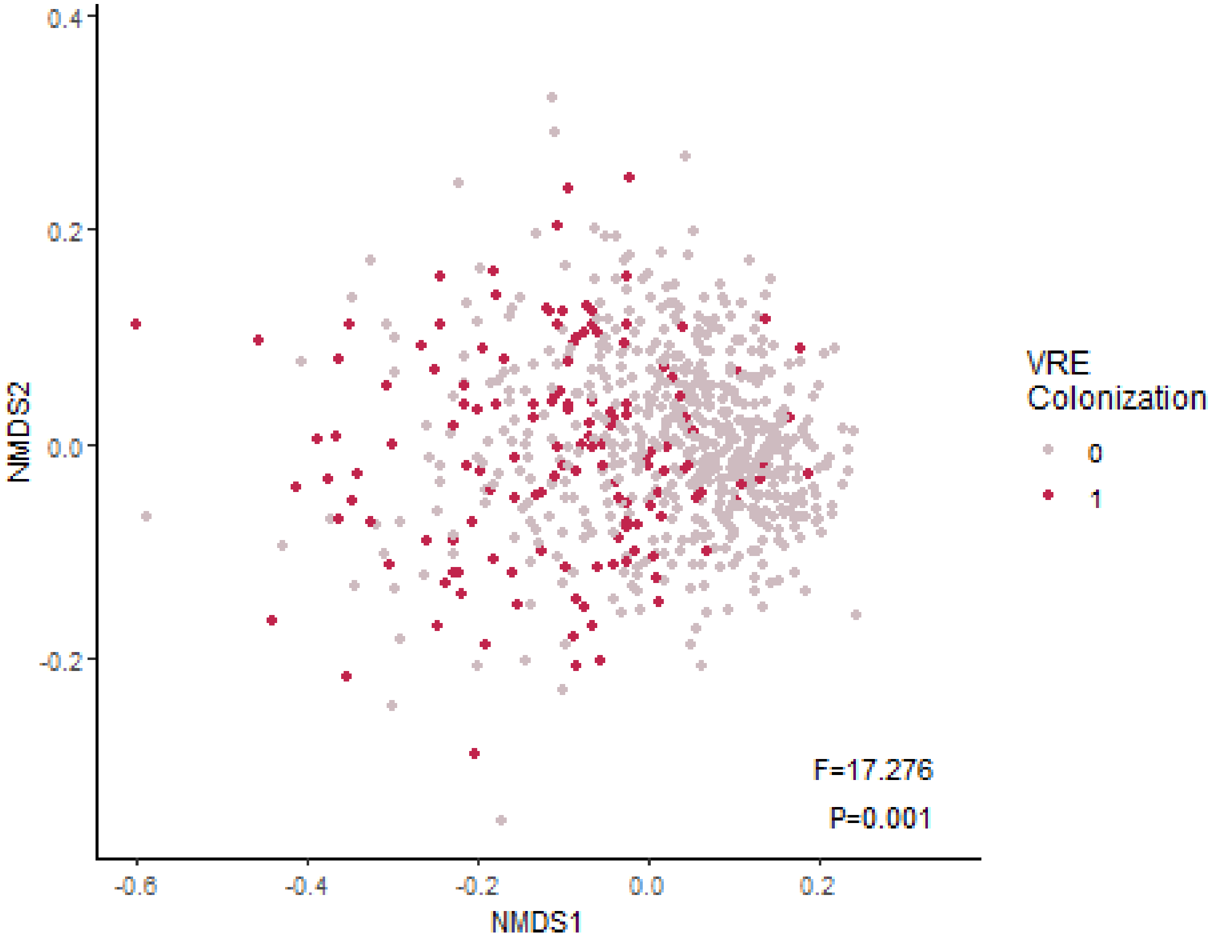

Putting it all together

```r
p1_a <- arrangeGrob(p1_a, top = textGrob("A", x = unit(0, "npc")
          , y   = unit(1, "npc"), just=c("left","top"),
          gp=gpar(col="black", fontsize=18, fontfamily="Arial")))

p1_b <- arrangeGrob(p1_b, top = textGrob("B", x = unit(0, "npc")
          , y   = unit(1, "npc"), just=c("left","top"),
          gp=gpar(col="black", fontsize=18, fontfamily="Arial")))

p1_c <- arrangeGrob(p1_c, top = textGrob("C", x = unit(0, "npc")
          , y   = unit(1, "npc"), just=c("left","top"),
          gp=gpar(col="black", fontsize=18, fontfamily="Arial")))
```

```
p1_d <- arrangeGrob(p1_d, top = textGrob("D", x = unit(0, "npc")
         , y    = unit(1, "npc"), just=c("left","top"),
         gp=gpar(col="black", fontsize=18, fontfamily="Arial")))

grid.arrange(p1_a,p1_b,p1_c,p1_d)
```

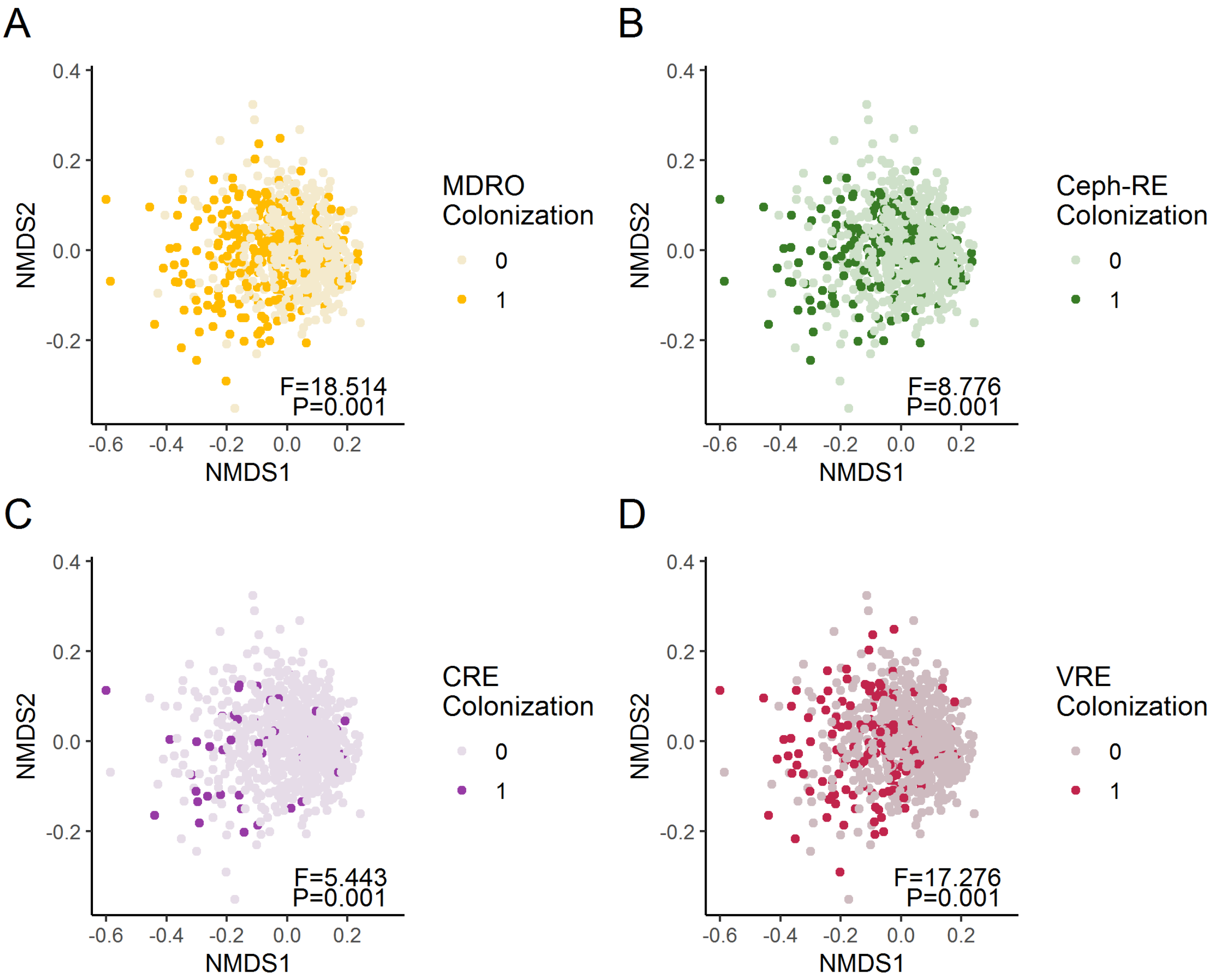

Supplement: Supplementary file 29 — Source Data [file 41467_2019_12633_MOESM29_ESM.zip › Source_Data/Figure1_MDRO_UF.pdf]
