# Supplementary material for: Colonizing multidrug-resistant bacteria and the longitudinal evolution of the intestinal microbiome after liver transplantation
Source: Nat Commun. 2019 Oct 17;10:4715. doi: 10.1038/s41467-019-12633-4 (PMC6797753; doi:10.1038/s41467-019-12633-4)
Supplement: Supplementary file 29 — Source Data [file 41467_2019_12633_MOESM29_ESM.zip › Source_Data/Figure2_PreLT_Diversity.pdf]

Liver transplant microbiome and MDRO

Medini K. Annavajhala

July 19, 2019

Generating Figure 2: Pre-transplant α- and β-diversity

This R Markdown document is part of a series used to analyze data and generate figures for the citation below. The current document includes all code and options used to generate Figure 2 from the manuscript, which looks at pre-liver transplant α- and β-diversity and underlying liver disease etiology and severity.

Title:

Colonizing multidrug-resistant organisms and the longitudinal evolution of the intestinal microbiome after liver transplantation

Authors:

Medini K. Annavajhala, Angela Gomez-Simmonds, Nenad Macesic, Sean B. Sullivan, Anna Kress, Sabrina D. Khan, Marla J. Giddins, Stephania Stump, Grace I. Kim, Ryan Narain, Elizabeth C. Verna, Anne-Catrin Uhlemann

Journal:

Nature Communications **2019**

Load Required Libraries:

```
library("phyloseq"); packageVersion("phyloseq")
```

```
## [1] '1.28.0'
```

```
library("biomformat"); packageVersion("biomformat")
```

```
## [1] '1.12.0'
```

```
library("data.table"); packageVersion("data.table")
```

```
## [1] '1.12.2'
```

```
library("vegan"); packageVersion("vegan")
```

```
## [1] '2.5.5'
```

```
library("ggplot2"); packageVersion("ggplot2")
```

```
## [1] '3.2.1'
```

```
library("grid"); packageVersion("grid")
```

```
## [1] '3.6.1'
```

```
library("gridExtra"); packageVersion("gridExtra")
```

```
## [1] '2.3'
```

```
library("ggsignif"); packageVersion("ggsignif")
```

```
## [1] '0.5.0'
```

```
library("dplyr"); packageVersion("dplyr")
```

```
## [1] '0.8.3'
```

Generate Figure 2 panels A, C, E

First, we import metadata and corresponding α-diversity values for the 83 pre-transplant samples (see R Markdown file "Phyloseq_Objects.Rmd").

In Figure 2, we looked at univariate associations between primary liver disease etiology, model for end-stage liver disease (MELD) at time of transplant (2016 calculator), and Child-Turcotte-Pugh (CTP) score (fixed effects) and Shannon and Chao α-diversity (outcomes).

```
preLT_data <- read.table("inputs/Fig2_metadata.txt", header=T)
colnames(preLT_data)
```

```
## [1] "StoolID"           "Primary_Diagnosis" "MELD_2016_Tx"
## [4] "MELD_2016_HighLow" "Child_Pugh_Score"  "Child_Pugh_Class"
## [7] "variable"          "value"
```

```
# Primary_Diagnosis: primary underlying liver disease etiology (reason for transplant)
#       AIH: Autoimmune hepatitis (n=7)
#       ARLD: Alcohol-related liver disease (n=7)
#       BILIARY: Etiologies related to biliary complications (n=4)
#       HBV: Hepatitis B virus (n=8)
#       HCV: Hepatitis C virus (n=37)
#       NAFLD: Non-alcoholic fatty liver disease (n=14)
#       PCLD: Polycystic liver/kidney disease (n=2)
#       OTHER (n=4)
# MELD_2016_Tx: MELD at time of transplant, 2016 calculator
# MELD_2016_HighLow: above/below median MELD_2016_Tx (18)
# Child_Pugh_Score: CTP score
# Child_Pugh_Class: Standard definitions based on CTP score; A (5-6), B (7-9), C(10-13)
```

Panel A: Chao and Shannon α-diversity by liver disease etiology

We included etiologies with n > 5 in panel A. This excludes the "Biliary", "PCLD", and "Other" categories

```
df_a <- preLT_data[!(preLT_data$Primary_Diagnosis %in% c("BILIARY","PCLD","OTHER")),]
p2_a <- ggplot(data = df_a,
              mapping = aes(Primary_Diagnosis, value,
                            color=Primary_Diagnosis, fill= Primary_Diagnosis)) +
          geom_point(width=0.10, size = 2, alpha=0.6) +
          geom_path() +
          facet_wrap(~variable, ncol = 2, scales = "free_y") +
          geom_boxplot(alpha = 0.6) +
          ylab("") +
          xlab("") +
          labs(color = "Primary\nIndication", fill = "Primary\nIndication") +
          scale_color_manual(values = c("AIH"="#7FC97F","ARLD"="#F0027F","HBV"="#FDC086",
                                        "HCV"="#FFFF99","NAFLD"="#386CB0")) +
          scale_fill_manual(values = c("AIH"="#7FC97F","ARLD"="#F0027F","HBV"="#FDC086",
                                        "HCV"="#FFFF99","NAFLD"="#386CB0")) +
          theme_classic() +
          theme(panel.grid.major = element_blank(), panel.grid.minor = element_blank(),
```

```
                 panel.background = element_blank(), panel.border = element_rect(fill=NA),
                 axis.line = element_line(colour = "black"),
                 axis.text.x = element_blank(),
                 strip.background = element_blank(), strip.text = element_text(size=12),
                 legend.position="right", legend.justification="left",
                 legend.margin=margin(0,0,0,0),legend.box.margin=margin(0,0,0,0))
```

We want to add some annotations for statistical comparisons; for univariate comparisons pre-LT (no repeated measures), we used a linear regression model with underlying diagnosis as the independent and α-diversity as the outcome.

ARLD clearly had the lowest α-diversity upon visual inspection of p2_a; so, we re-factor the Primary_Diagnosis variable to keep ARLD as the Reference level and compare all other etiologies against ARLD.

```
preLT_data$Primary_Diagnosis = relevel(preLT_data$Primary_Diagnosis, ref = "ARLD")

summary(lm(value ~ Primary_Diagnosis, data=preLT_data[(preLT_data$variable=="Shannon"),]))
```

```
##
## Call:
## lm(formula = value ~ Primary_Diagnosis, data = preLT_data[(preLT_data$variable ==
##     "Shannon"), ])
##
## Residuals:
##     Min      1Q  Median      3Q     Max
## -2.6403 -0.5089  0.1284  0.6172  1.3301
##
## Coefficients:
##                            Estimate Std. Error t value Pr(>|t|)
## (Intercept)                  1.4358     0.3355   4.280 5.46e-05 ***
## Primary_DiagnosisAIH         1.2983     0.4745   2.736 0.007751 **
## Primary_DiagnosisBILIARY     0.9100     0.5564   1.636 0.106119
## Primary_DiagnosisHBV         1.3879     0.4594   3.021 0.003442 **
## Primary_DiagnosisHCV         1.4540     0.3659   3.974 0.000161 ***
## Primary_DiagnosisNAFLD       1.4789     0.4109   3.599 0.000570 ***
## Primary_DiagnosisOTHER       2.2935     0.5564   4.122 9.58e-05 ***
## Primary_DiagnosisPCLD        2.0249     0.7117   2.845 0.005719 **
## ---
## Signif. codes:  0 '***' 0.001 '**' 0.01 '*' 0.05 '.' 0.1 ' ' 1
##
## Residual standard error: 0.8876 on 75 degrees of freedom
## Multiple R-squared:   0.24,  Adjusted R-squared:  0.1691
## F-statistic: 3.384 on 7 and 75 DF,  p-value: 0.003417
```

```
summary(lm(value ~ Primary_Diagnosis, data=preLT_data[(preLT_data$variable=="Chao"),]))
```

```
##
## Call:
## lm(formula = value ~ Primary_Diagnosis, data = preLT_data[(preLT_data$variable ==
##     "Chao"), ])
##
## Residuals:
##      Min       1Q   Median       3Q      Max
## -153.716  -56.061   -1.549   45.549  160.137
##
```

```
## Coefficients:
##                           Estimate Std. Error t value Pr(>|t|)
## (Intercept)                 160.21      29.76   5.383 8.07e-07 ***
## Primary_DiagnosisAIH         30.92      42.09   0.735   0.4648
## Primary_DiagnosisBILIARY     31.33      49.35   0.635   0.5274
## Primary_DiagnosisHBV         94.41      40.75   2.317   0.0233 *
## Primary_DiagnosisHCV         58.01      32.46   1.787   0.0779 .
## Primary_DiagnosisNAFLD       48.37      36.45   1.327   0.1886
## Primary_DiagnosisOTHER       76.14      49.35   1.543   0.1271
## Primary_DiagnosisPCLD        95.79      63.13   1.517   0.1334
## ---
## Signif. codes:  0 '***' 0.001 '**' 0.01 '*' 0.05 '.' 0.1 ' ' 1
##
## Residual standard error: 78.74 on 75 degrees of freedom
## Multiple R-squared:  0.08948,    Adjusted R-squared:  0.004497
## F-statistic: 1.053 on 7 and 75 DF,  p-value: 0.4023
```

```
#ARLD has significantly lower Chao and Shannon diversity:
#Shannon: ARLD vs. AIH (p<0.01,**),HBV (p<0.01,**),HCV (p<0.001,***),NAFLD (p<0.001,***)
#Chao: ARLD vs. HBV (p<0.05,*),HCV (p<0.1,+)


annotation_dfa <- data.frame(variable=c("Chao","Chao","Shannon","Shannon",
                                        "Shannon","Shannon"),
                            start=c("ARLD","ARLD","ARLD","ARLD","ARLD","ARLD"),
                            end=c("HBV","HCV","AIH","HBV","HCV","NAFLD"),
                            y=c(90,50,3.45,3.75,0.45,0.05),
                            label=c("*", "+","**","**","***","***"))

p2_a <- ggplot(data = df_a,
              mapping = aes(Primary_Diagnosis, value,
                            color=Primary_Diagnosis, fill= Primary_Diagnosis)) +
          geom_point(width=0.10, size = 2, alpha=0.6) +
          geom_path() +
          geom_signif(inherit.aes = FALSE, data=annotation_dfa,
              aes(xmin=start, xmax=end, annotations=label, y_position=y),
              textsize = 3, vjust = 0.0, tip_length = c(0,0),
              manual=TRUE) +
          facet_wrap(~variable, ncol = 2, scales = "free_y") +
          geom_boxplot(alpha = 0.6) +
          ylab("") +
          xlab("") +
          labs(color = "Primary\nIndication", fill = "Primary\nIndication") +
          scale_color_manual(values = c("AIH"="#7FC97F","ARLD"="#F0027F","HBV"="#FDC086",
                                        "HCV"="#FFFF99","NAFLD"="#386CB0")) +
          scale_fill_manual(values = c("AIH"="#7FC97F","ARLD"="#F0027F","HBV"="#FDC086",
                                        "HCV"="#FFFF99","NAFLD"="#386CB0")) +
          theme_classic() +
          theme(panel.grid.major = element_blank(), panel.grid.minor = element_blank(),
                panel.background = element_blank(), panel.border = element_rect(fill=NA),
                axis.line = element_line(colour = "black"),
                axis.text.x = element_blank(),
                strip.background = element_blank(), strip.text = element_text(size=12),
                legend.position="right", legend.justification="left",
```

```
                    legend.margin=margin(0,0,0,0),legend.box.margin=margin(0,0,0,0))

p2_a
```

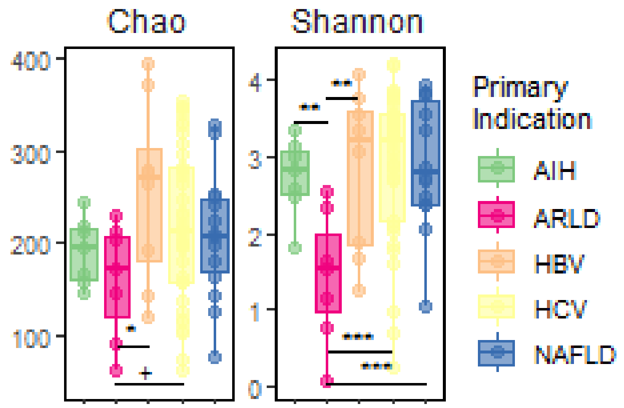

Panel C: Chao and Shannon α-diversity by transplant MELD

Then we plot both Chao and Shannon diversity indices grouped by above/below median transplant MELD (> 18 vs. <=18). As with panel A, we add in the annotation for the statistical comparisons

```
preLT_data$MELD_2016_HighLow = factor(preLT_data$MELD_2016_HighLow,levels=c("LOW","HIGH"))

summary(lm(value ~ MELD_2016_HighLow, data=preLT_data[(preLT_data$variable=="Shannon"),]))
```

```
##
## Call:
## lm(formula = value ~ MELD_2016_HighLow, data = preLT_data[(preLT_data$variable ==
##     "Shannon"), ])
##
## Residuals:
##     Min      1Q  Median      3Q     Max
## -2.3314 -0.5129  0.1329  0.5534  1.9152
##
## Coefficients:
##                       Estimate Std. Error t value Pr(>|t|)
## (Intercept)             3.2907     0.1333  24.678  < 2e-16 ***
## MELD_2016_HighLowHIGH  -0.9860     0.1853  -5.322 8.93e-07 ***
## ---
## Signif. codes:  0 '***' 0.001 '**' 0.01 '*' 0.05 '.' 0.1 ' ' 1
##
## Residual standard error: 0.8433 on 81 degrees of freedom
## Multiple R-squared:  0.2591, Adjusted R-squared:   0.25
## F-statistic: 28.33 on 1 and 81 DF,  p-value: 8.934e-07
```

```
summary(lm(value ~ MELD_2016_HighLow, data=preLT_data[(preLT_data$variable=="Chao"),]))
```

```
##
## Call:
## lm(formula = value ~ MELD_2016_HighLow, data = preLT_data[(preLT_data$variable ==
##     "Chao"), ])
```

```
##
## Residuals:
##     Min      1Q  Median      3Q     Max
## -189.19  -48.38   -2.96   48.00  169.04
##
## Coefficients:
##                       Estimate Std. Error t value Pr(>|t|)
## (Intercept)             253.69      10.91  23.256  < 2e-16 ***
## MELD_2016_HighLowHIGH   -77.73      15.16  -5.129 1.95e-06 ***
## ---
## Signif. codes:  0 '***' 0.001 '**' 0.01 '*' 0.05 '.' 0.1 ' ' 1
##
## Residual standard error: 68.99 on 81 degrees of freedom
## Multiple R-squared:  0.2451, Adjusted R-squared:  0.2358
## F-statistic:  26.3 on 1 and 81 DF,  p-value: 1.953e-06
```

```
#High transplant MELD is associated with significantly lower Shannon (p<0.0001, ****)
#and Chao (p<0.0001, ****) $\alpha$-diversity

annotation_dfc <- data.frame(variable=c("Chao","Shannon"),
                             start=c("LOW","LOW"),
                             end=c("HIGH","HIGH"),
                             y=c(350,4.0),
                             label=c("****","****"))

p2_c <- ggplot(data = preLT_data,
              mapping = aes(MELD_2016_HighLow, value,
                            color=MELD_2016_HighLow, fill= MELD_2016_HighLow)) +
          geom_point(width=0.10, size = 2, alpha=0.6) +
          geom_path() +
          geom_signif(inherit.aes = FALSE, data=annotation_dfc,
              aes(xmin=start, xmax=end, annotations=label, y_position=y),
              textsize = 3, vjust = 0.0, tip_length = c(0,0),
              manual=TRUE) +
          facet_wrap(~variable, ncol = 2, scales = "free_y") +
          geom_boxplot(alpha = 0.6) +
          ylab("") +
          xlab("") +
          labs(color = "MELD\n(2016)", fill = "MELD\n(2016)") +
          scale_color_manual(values = c("LOW"="#2d51b5","HIGH"="#c70643")) +
          scale_fill_manual(values = c("LOW"="#2d51b5","HIGH"="#c70643")) +
          theme_classic() +
          theme(panel.grid.major = element_blank(), panel.grid.minor = element_blank(),
                panel.background = element_blank(), panel.border = element_rect(fill=NA),
                axis.line = element_line(colour = "black"),
                axis.text.x = element_blank(),
                strip.background = element_blank(), strip.text = element_text(size=12),
                legend.position="right", legend.justification="left",
                legend.margin=margin(0,0,0,0),legend.box.margin=margin(0,0,0,0))

p2_c
```

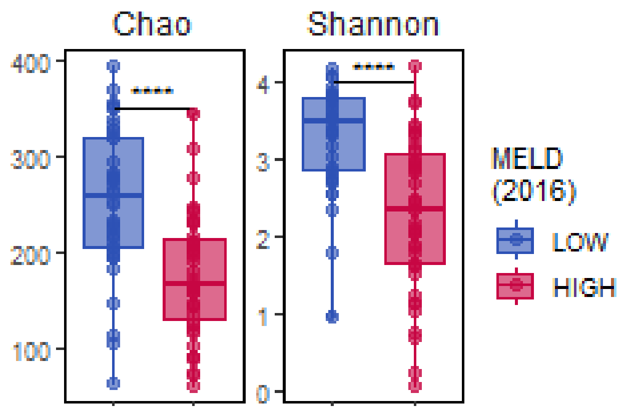

Panel E: Chao and Shannon α-diversity by transplant CTP

Same thing; we then plot both Chao and Shannon diversity indices grouped by Child-Turcotte-Pugh (CTP) class (classes A, B, C)

```
preLT_data$Child_Pugh_Class = relevel(preLT_data$Child_Pugh_Class, ref="A")

summary(lm(value ~ Child_Pugh_Class, data=preLT_data[(preLT_data$variable=="Shannon"),]))
```

```
##
## Call:
## lm(formula = value ~ Child_Pugh_Class, data = preLT_data[(preLT_data$variable ==
##     "Shannon"), ])
##
## Residuals:
##     Min      1Q  Median      3Q     Max
## -2.2240 -0.4372  0.1747  0.5261  1.9115
##
## Coefficients:
##                   Estimate Std. Error t value Pr(>|t|)
## (Intercept)         3.5725     0.2087  17.119  < 2e-16 ***
## Child_Pugh_ClassB  -0.4762     0.2694  -1.768   0.0809 .
## Child_Pugh_ClassC  -1.2641     0.2444  -5.171 1.68e-06 ***
## ---
## Signif. codes:  0 '***' 0.001 '**' 0.01 '*' 0.05 '.' 0.1 ' ' 1
##
## Residual standard error: 0.8347 on 80 degrees of freedom
## Multiple R-squared:  0.2831, Adjusted R-squared:  0.2652
## F-statistic:  15.8 on 2 and 80 DF,  p-value: 1.653e-06
```

```
summary(lm(value ~ Child_Pugh_Class, data=preLT_data[(preLT_data$variable=="Chao"),]))
```

```
##
## Call:
## lm(formula = value ~ Child_Pugh_Class, data = preLT_data[(preLT_data$variable ==
##     "Chao"), ])
##
## Residuals:
##     Min      1Q  Median      3Q     Max
## -161.96  -53.20   -5.86   45.45  172.82
```

```
##
## Coefficients:
##                    Estimate Std. Error t value Pr(>|t|)
## (Intercept)          286.74      16.99  16.881  < 2e-16 ***
## Child_Pugh_ClassB    -60.28      21.93  -2.749  0.00739 **
## Child_Pugh_ClassC   -107.88      19.90  -5.422 6.09e-07 ***
## ---
## Signif. codes:  0 '***' 0.001 '**' 0.01 '*' 0.05 '.' 0.1 ' ' 1
##
## Residual standard error: 67.94 on 80 degrees of freedom
## Multiple R-squared:  0.2769, Adjusted R-squared:  0.2589
## F-statistic: 15.32 on 2 and 80 DF,  p-value: 2.328e-06
```

```
preLT_data$Child_Pugh_Class = relevel(preLT_data$Child_Pugh_Class, ref="B")
summary(lm(value ~ Child_Pugh_Class, data=preLT_data[(preLT_data$variable=="Shannon"),]))
```

```
##
## Call:
## lm(formula = value ~ Child_Pugh_Class, data = preLT_data[(preLT_data$variable ==
##     "Shannon"), ])
##
## Residuals:
##     Min      1Q  Median      3Q     Max
## -2.2240 -0.4372  0.1747  0.5261  1.9115
##
## Coefficients:
##                    Estimate Std. Error t value Pr(>|t|)
## (Intercept)          3.0962     0.1704  18.172  < 2e-16 ***
## Child_Pugh_ClassA    0.4762     0.2694   1.768 0.080923 .
## Child_Pugh_ClassC   -0.7879     0.2127  -3.704 0.000388 ***
## ---
## Signif. codes:  0 '***' 0.001 '**' 0.01 '*' 0.05 '.' 0.1 ' ' 1
##
## Residual standard error: 0.8347 on 80 degrees of freedom
## Multiple R-squared:  0.2831, Adjusted R-squared:  0.2652
## F-statistic:  15.8 on 2 and 80 DF,  p-value: 1.653e-06
```

```
summary(lm(value ~ Child_Pugh_Class, data=preLT_data[(preLT_data$variable=="Chao"),]))
```

```
##
## Call:
## lm(formula = value ~ Child_Pugh_Class, data = preLT_data[(preLT_data$variable ==
##     "Chao"), ])
##
## Residuals:
##     Min      1Q  Median      3Q     Max
## -161.96  -53.20   -5.86   45.45  172.82
##
## Coefficients:
##                    Estimate Std. Error t value Pr(>|t|)
## (Intercept)          226.46      13.87  16.329  < 2e-16 ***
## Child_Pugh_ClassA     60.28      21.93   2.749  0.00739 **
## Child_Pugh_ClassC    -47.60      17.31  -2.749  0.00738 **
## ---
## Signif. codes:  0 '***' 0.001 '**' 0.01 '*' 0.05 '.' 0.1 ' ' 1
```

```
##
## Residual standard error: 67.94 on 80 degrees of freedom
## Multiple R-squared:  0.2769, Adjusted R-squared:  0.2589
## F-statistic: 15.32 on 2 and 80 DF,  p-value: 2.328e-06
```

```r
#CTP Class is associated with significantly lower $\alpha$-diversity:
#Shannon: A vs B (p=0.08, +), A vs C (p<0.0001, ****), B vs C (p<0.001, ***)
#Chao: A vs B (p<0.01, **), A vs C (p<0.0001, ****), B vs C (p<0.01, **)

annotation_dfe <- data.frame(variable=c("Chao","Chao","Chao","Shannon",
                                        "Shannon","Shannon"),
                             start=c("A","A","B","A","A","B"),
                             end=c("B","C","C","B","C","C"),
                             y=c(140,100,60,1.5,1.0,0.5),
                             label=c("**","****","**","+","****","***"))

preLT_data$Child_Pugh_Class = factor(preLT_data$Child_Pugh_Class, levels=c("A","B","C"))

p2_e <- ggplot(data = preLT_data,
              mapping = aes(Child_Pugh_Class, value,
                            color=Child_Pugh_Class, fill= Child_Pugh_Class)) +
          geom_point(width=0.10, size = 2, alpha=0.6) +
          geom_path() +
          geom_signif(inherit.aes = FALSE, data=annotation_dfe,
              aes(xmin=start, xmax=end, annotations=label, y_position=y),
              textsize = 3, vjust = 0.0, tip_length = c(0,0),
              manual=TRUE) +
          facet_wrap(~variable, ncol = 2, scales = "free_y") +
          geom_boxplot(alpha = 0.6) +
          ylab("") +
          xlab("") +
          labs(color = "CTP\nClass", fill = "CTP\nClass") +
          scale_color_manual(values = c("A"="#6FB98F","B"="#2C7873","C"="#004445")) +
          scale_fill_manual(values = c("A"="#6FB98F","B"="#2C7873","C"="#004445")) +
          theme_classic() +
          theme(panel.grid.major = element_blank(), panel.grid.minor = element_blank(),
                panel.background = element_blank(), panel.border = element_rect(fill=NA),
                axis.line = element_line(colour = "black"),
                axis.text.x = element_blank(),
                strip.background = element_blank(), strip.text = element_text(size=12),
                legend.position="right", legend.justification="left",
                legend.margin=margin(0,0,0,0),legend.box.margin=margin(0,0,0,0))

p2_e
```

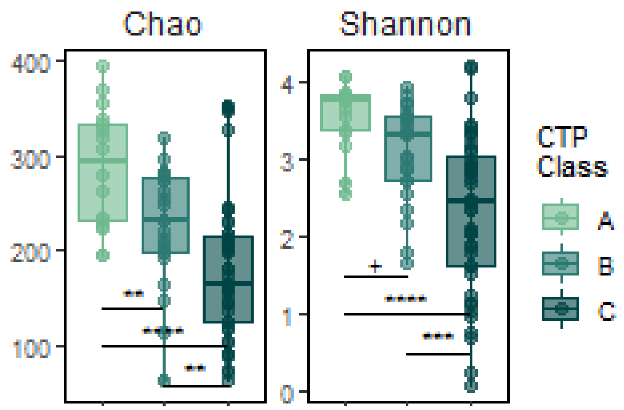

Generate Figure 2 panels B, D, F

First, we import the phyloseq objects we made in our earlier markdown file - we will need the relative abundance data to calculate and plot β-diversity metrics by our metadata categories. After importing, we need to subset our phyloseq objects to retain only pre-LT samples and add in the metadata we used above for panels A, C, and E.

```
phylo_relabun_filtered = readRDS("inputs/phylo_relabun_filtered.RDS")

preLT_relabun = prune_samples(sample_names(phylo_relabun_filtered) %in%
                                preLT_data$StoolID, phylo_relabun_filtered)

df1 <- preLT_data[(preLT_data$variable=="Shannon"),1:6] #Metadata from data we used before
df2 <- data.frame(sample_data(preLT_relabun),
                  "StoolID"=rownames(sample_data(preLT_relabun))) #Sample names
merged <- merge(df2, df1, by="StoolID") #Merge preLT metadata with phyloseq object
merged <- sample_data(merged)
sample_names(merged) = merged$StoolID #fix names
sample_data(preLT_relabun) <- sample_data(merged)
```

Now we can calculate UniFrac β-diversity

```
DistUF = phyloseq::distance(preLT_relabun, "uniFrac")
ordUF = ordinate(preLT_relabun, method = "PCoA", distance = DistUF)
```

The `adonis` function from the `vegan` package is used to run a permutational ANOVA (PERMANOVA) to test for significant clustering of β-diversity by variable, as shown below

Panel B

```
p2_b = plot_ordination(preLT_relabun, ordUF, color = "Primary_Diagnosis") +
  geom_point(size=1.5, alpha=0.2) +
  labs(color="") +
  ylim(-0.50,0.50) +
  xlim(-0.50,0.50)

#AIH and ARLD seem to clearly cluster together, but is this statistically significant?

metadata = data.frame(sample_data(preLT_relabun))
```

```r
#Make binary yes/no AIH and binary yes/no ARLD columns
set.seed(2)
metadata$AIH = with(metadata, ifelse(metadata$Primary_Diagnosis=="AIH","AIH","OTHER"))
metadata$ARLD = with(metadata, ifelse(metadata$Primary_Diagnosis=="ARLD","ARLD","OTHER"))

adonis(DistUF ~ ARLD, data=metadata) #P=0.014; yes (F =  1.842)
```

```
##
## Call:
## adonis(formula = DistUF ~ ARLD, data = metadata)
##
## Permutation: free
## Number of permutations: 999
##
## Terms added sequentially (first to last)
##
##           Df SumsOfSqs MeanSqs F.Model      R2 Pr(>F)
## ARLD       1    0.3362 0.33619   1.842 0.02224  0.013 *
## Residuals 81   14.7834 0.18251         0.97776
## Total     82   15.1196                 1.00000
## ---
## Signif. codes:  0 '***' 0.001 '**' 0.01 '*' 0.05 '.' 0.1 ' ' 1
```

```r
adonis(DistUF ~ AIH, data=metadata) #P=0.312; no! (checked others, also not significant)
```

```
##
## Call:
## adonis(formula = DistUF ~ AIH, data = metadata)
##
## Permutation: free
## Number of permutations: 999
##
## Terms added sequentially (first to last)
##
##           Df SumsOfSqs MeanSqs F.Model      R2 Pr(>F)
## AIH        1    0.1964 0.19636  1.0658 0.01299  0.313
## Residuals 81   14.9233 0.18424         0.98701
## Total     82   15.1196                 1.00000
```

```r
# Make the plot
sample_data(preLT_relabun)$Primary_Diagnosis=relevel(
                          sample_data(preLT_relabun)$Primary_Diagnosis, ref="AIH")
p2_b = p2_b +  stat_ellipse(data = subset(p2_b$data, Primary_Diagnosis=="ARLD"),
               type="norm",linetype = 2, lwd=0.8, geom="polygon", alpha=0.05,
               aes(fill=Primary_Diagnosis), show.legend = FALSE) +
  scale_color_manual(values = c("AIH"="#7FC97F","ARLD"="#F0027F","HBV"="#FDC086",
                                        "HCV"="#FFFF99","NAFLD"="#386CB0",
                                        "BILIARY"="#BEAED4","PCLD"="#f542c5",
                                "OTHER"="#c43996")) +
  scale_fill_manual(values = c("ARLD"="#F0027F"), guide=F) +
  annotate(geom="text",x = -0.10, y=-0.35,label="F(ARLD)=1.842",hjust=0) +
  annotate(geom="text",x = -0.10, y=-0.45,label="P(ARLD)=0.014",hjust=0) +
  theme_classic() +
  theme(legend.margin = margin(0,0,0,0))
```

```
p2_b
```

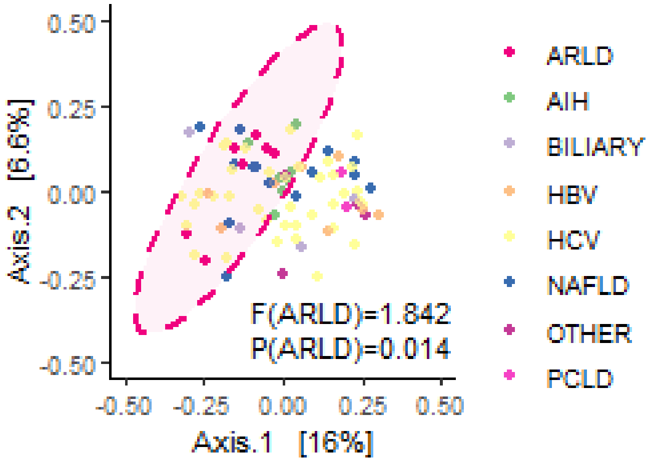

Panel D

```
sample_data(preLT_relabun)$MELD_2016_HighLow = factor(
                            sample_data(preLT_relabun)$MELD_2016_HighLow,
                            levels = c("LOW","HIGH"))

#Do samples cluster by transplant MELD?
adonis(DistUF ~ MELD_2016_HighLow, data=metadata) #P=0.001; yes (F=4.0099)
```

```
##
## Call:
## adonis(formula = DistUF ~ MELD_2016_HighLow, data = metadata)
##
## Permutation: free
## Number of permutations: 999
##
## Terms added sequentially (first to last)
##
##                   Df SumsOfSqs MeanSqs F.Model      R2 Pr(>F)
## MELD_2016_HighLow  1    0.7132 0.71319  4.0099 0.04717  0.001 ***
## Residuals         81   14.4064 0.17786         0.95283
## Total             82   15.1196                 1.00000
## ---
## Signif. codes:  0 '***' 0.001 '**' 0.01 '*' 0.05 '.' 0.1 ' ' 1
```

```
p2_d = plot_ordination(preLT_relabun, ordUF, color = "MELD_2016_HighLow") +
  geom_point(size=1.5, alpha=0.2) +
  labs(color="") +
  ylim(-0.50,0.50) +
  xlim(-0.50,0.50) +
  scale_color_manual(values = c("LOW"="#2d51b5","HIGH"="#c70643")) +
  annotate(geom="text",x = 0.20, y=-0.35,label="F=4.010",hjust=0) +
  annotate(geom="text",x = 0.20, y=-0.45,label="P=0.001",hjust=0) +
  stat_ellipse() +
  theme_classic()

p2_d
```

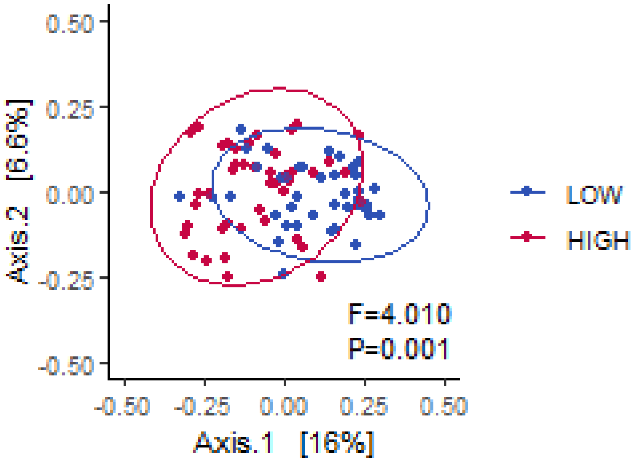

####Panel F

```
#Do samples cluster by transplant CTP?
adonis(DistUF ~ Child_Pugh_Class, data=metadata) #P=0.001; yes (F=2.8806)
```

```
##
## Call:
## adonis(formula = DistUF ~ Child_Pugh_Class, data = metadata)
##
## Permutation: free
## Number of permutations: 999
##
## Terms added sequentially (first to last)
##
##                  Df SumsOfSqs MeanSqs F.Model      R2 Pr(>F)
## Child_Pugh_Class  2    1.0157 0.50784  2.8806 0.06718  0.001 ***
## Residuals        80   14.1039 0.17630         0.93282
## Total            82   15.1196                 1.00000
## ---
## Signif. codes:  0 '***' 0.001 '**' 0.01 '*' 0.05 '.' 0.1 ' ' 1
```

```
p2_f = plot_ordination(preLT_relabun, ordUF, color = "Child_Pugh_Class") +
  geom_point(size=1.5, alpha=0.2) +
  labs(color="") +
  ylim(-0.50,0.50) +
  xlim(-0.50,0.50) +
  scale_color_manual(values = c("A"="#6FB98F","B"="#2C7873","C"="#004445")) +
  annotate(geom="text",x = 0.20, y=-0.35,label="F=2.881", hjust=0) +
  annotate(geom="text",x = 0.20, y=-0.45,label="P=0.001", hjust=0) +
  stat_ellipse() +
  theme_classic()

p2_f
```

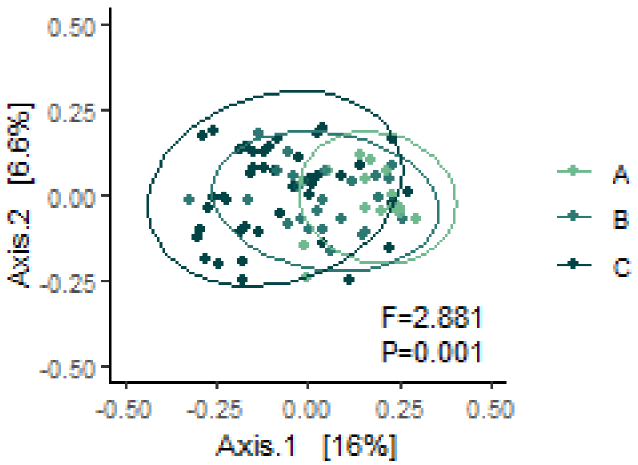

Finishing touches

The final Figure 2 incorporates the 6 plots produced in this markdown file.

```
p2_a <- arrangeGrob(p2_a, top = textGrob("A", x = unit(0, "npc")
         , y   = unit(1, "npc"), just=c("left","top"),
         gp=gpar(col="black", fontsize=18, fontfamily="Arial")))

p2_b <- arrangeGrob(p2_b, top = textGrob("B", x = unit(0, "npc")
         , y   = unit(1, "npc"), just=c("left","top"),
         gp=gpar(col="black", fontsize=18, fontfamily="Arial")))

p2_c <- arrangeGrob(p2_c, top = textGrob("C", x = unit(0, "npc")
         , y   = unit(1, "npc"), just=c("left","top"),
         gp=gpar(col="black", fontsize=18, fontfamily="Arial")))

p2_d <- arrangeGrob(p2_d, top = textGrob("D", x = unit(0, "npc")
         , y   = unit(1, "npc"), just=c("left","top"),
         gp=gpar(col="black", fontsize=18, fontfamily="Arial")))

p2_e <- arrangeGrob(p2_e, top = textGrob("E", x = unit(0, "npc")
         , y   = unit(1, "npc"), just=c("left","top"),
         gp=gpar(col="black", fontsize=18, fontfamily="Arial")))

p2_f <- arrangeGrob(p2_f, top = textGrob("F", x = unit(0, "npc")
         , y   = unit(1, "npc"), just=c("left","top"),
         gp=gpar(col="black", fontsize=18, fontfamily="Arial")))


grid.arrange(p2_a,p2_b,p2_c,p2_d,p2_e,p2_f)
```

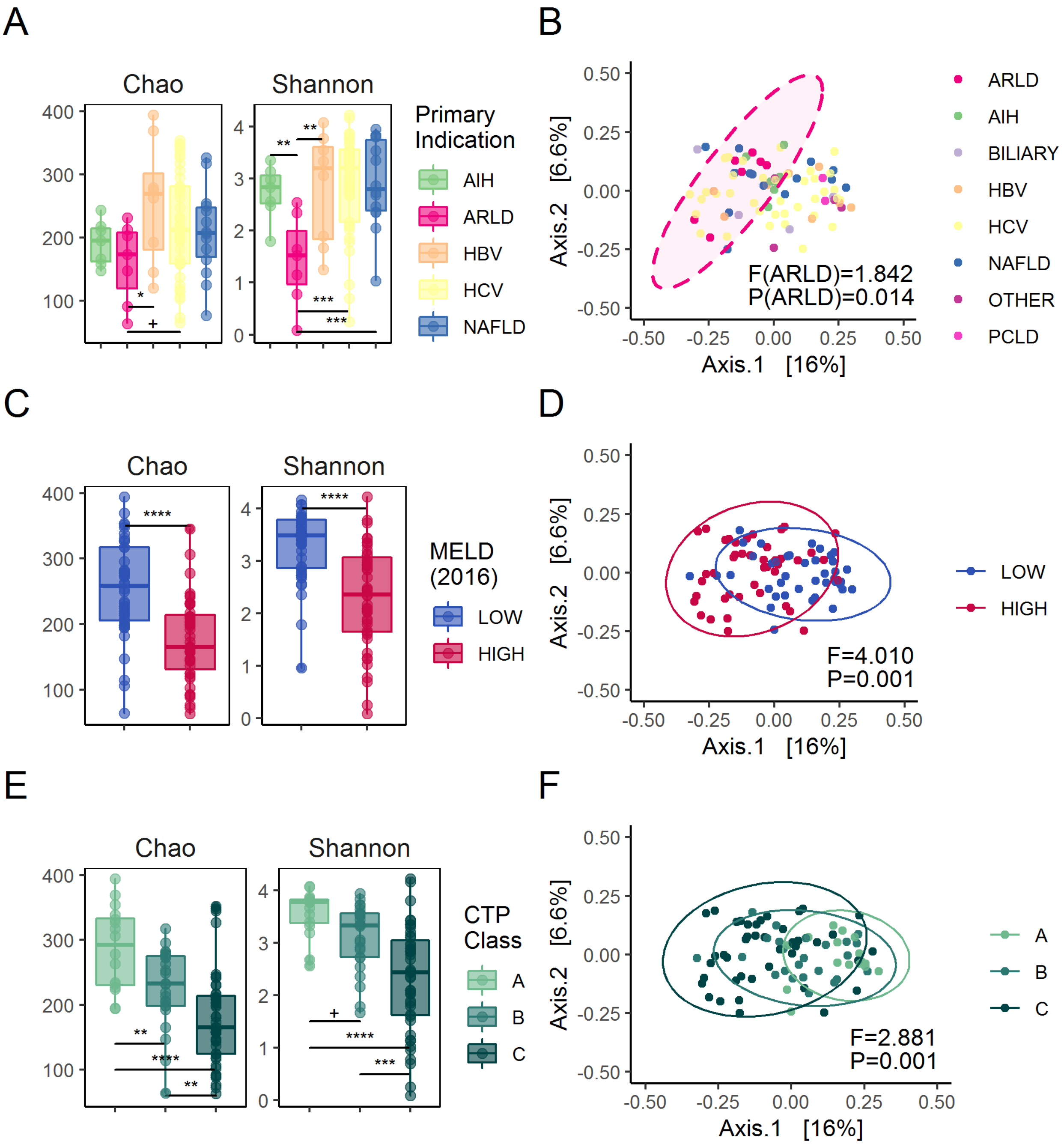
A
Chao
Shannon
Primary Indication
AIH
ARLD
HBV
HCV
NAFLD
B
F(ARLD)=1.842
P(ARLD)=0.014
Axis.1 [16%]
Axis.2 [6.6%]
ARLD
AIH
BILIARY
HBV
HCV
NAFLD
OTHER
PCLD
C
Chao
Shannon
MELD (2016)
LOW
HIGH
D
F=4.010
P=0.001
Axis.1 [16%]
Axis.2 [6.6%]
LOW
HIGH
E
Chao
Shannon
CTP Class
A
B
C
F
F=2.881
P=0.001
Axis.1 [16%]
Axis.2 [6.6%]
A
B
C
