# Supplementary material for: Colonizing multidrug-resistant bacteria and the longitudinal evolution of the intestinal microbiome after liver transplantation
Source: Nat Commun. 2019 Oct 17;10:4715. doi: 10.1038/s41467-019-12633-4 (PMC6797753; doi:10.1038/s41467-019-12633-4)
Supplement: Supplementary file 29 — Source Data [file 41467_2019_12633_MOESM29_ESM.zip › Source_Data/Figure3_PostLT_Diversity.pdf]

Liver transplant microbiome and MDRO

Medini K. Annavajhala

July 19, 2019

Generating Figure 3: Post-LT microbiome diversity

This R Markdown document is part of a series used to analyze data and generate figures for the citation below. The current document includes all code and options used to generate Figure 3 from the manuscript, which shows the Shannon and Chao α–diversity and UniFrac β-diversity for each of the six major underlying liver disease etiologies over the study period. Results of the constrained linear mixed-effect regression (CLME) analyses are also provided as Supplementary Data files 9 and 10 with the citation below.

Title:

Colonizing multidrug-resistant organisms and the longitudinal evolution of the intestinal microbiome after liver transplantation

Authors:

Medini K. Annavajhala, Angela Gomez-Simmonds, Nenad Macesic, Sean B. Sullivan, Anna Kress, Sabrina D. Khan, Marla J. Giddins, Stephania Stump, Grace I. Kim, Ryan Narain, Elizabeth C. Verna, Anne-Catrin Uhlemann

Journal:

Nature Communications **2019**

Load Required Libraries:

```
library("phyloseq"); packageVersion("phyloseq")
```

```
## [1] '1.28.0'
```

```
library("ggplot2"); packageVersion("ggplot2")
```

```
## [1] '3.2.1'
```

```
library("lemon"); packageVersion("lemon")
```

```
## [1] '0.4.3'
```

```
library("CLME"); packageVersion("CLME")
```

```
## [1] '2.0.11'
```

```
library("ggrepel"); packageVersion("ggrepel")
```

```
## [1] '0.8.1'
```

```
library("egg"); packageVersion("egg")
```

```
## [1] '0.4.5'
```

```
library("grid"); packageVersion("grid")
```

```
## [1] '3.6.1'
```

Generate Panels A,B (α-diversity)

First, we import metadata and corresponding α-diversity values for all samples (see R Markdown file "Phyloseq_Objects.Rmd")

```
LT_data <- read.table("inputs/Fig3_metadata.txt", header=T)
colnames(LT_data)
```

```
## [1] "StoolID"            "StudyID"            "Days_Post_LT"
## [4] "Time_Category"      "Time_Category2"     "Primary_Diagnosis"
## [7] "Shannon"            "Chao"
```

```
# Time_Category: One of 10 pre-selected sampling timepoints; pre-LT, then
#                    Weeks 1, 2, 3 and Months 1, 2, 3, 6, 9, and 12 post-LT
# Time_Category2: Timepoints split into 4 periods; pre-LT, peri-LT (Weeks 1-3),
#                    early post-LT (Months 1-3), late post-LT (Months 6-12)
# Primary_Diagnosis: primary underlying liver disease etiology (reason for LT)
#       AIH: Autoimmune hepatitis
#       ARLD: Alcohol-related liver disease
#       BILIARY: Etiologies related to biliary complications
#       HBV: Hepatitis B virus
#       HCV: Hepatitis C virus
#       NAFLD: Non-alcoholic fatty liver disease
#       PCLD: Polycystic liver/kidney disease
#       OTHER
# Shannon: Shannon $\alpha$-diversity index (richness + evenness)
# Chao: Chao $\alpha$-diversity index (richness)
```

Panel A: Shannon α-diversity over time

Next, we want to plot the longitudinal data set for each patient - α-diversity vs. days post-LT for both Shannon (Figure 3A) and Chao (Figure 3B) indices

```
palette_a = c("#8DD3C7","#FFFFB3","#BEBADA","#FB8072","#80B1D3","#FDB462",
            "#B3DE69","#FF7256","#8B8378","#BC80BD","#7FFFD4")

p3_a = ggplot(LT_data, mapping = aes(x=Days_Post_LT, y=Shannon,
                      color=Time_Category, group=factor(StudyID))) +
  geom_line(color="grey") +
  geom_point(size = 1.25) +
  xlab("Days Post-Transplant") + ylab("Shannon Index\n") +
  ggtitle("") +
  facet_rep_wrap(~Primary_Diagnosis, ncol=3, scales = "fixed", repeat.tick.labels = T) +
  scale_color_manual(values=palette_a, labels=c("Pre-LT","Week 1","Week 2",
                                     "Week 3","Month 1","Month 2","Month 3",
                                     "Month 6","Month 9","Month 12")) +
  scale_x_continuous(limits=c(-250,570), breaks=c(-200,0,200,400)) +
  labs(color="Time Category") +
  theme(panel.grid = element_blank(), panel.background = element_blank(),
        panel.border = element_rect(fill=NA),
        axis.line = element_line(colour = "black"),
        strip.background = element_rect(fill=NA), strip.text = element_text(size=10),
        legend.title = element_text(size=10), legend.text= element_text(size=10),
        legend.key = element_blank(),plot.margin = margin(0,0,0,0),
        axis.text = element_text(size=10), axis.title.x=element_text(size=10),
        axis.title.y=element_text(size=12))
```

```
p3_a
```

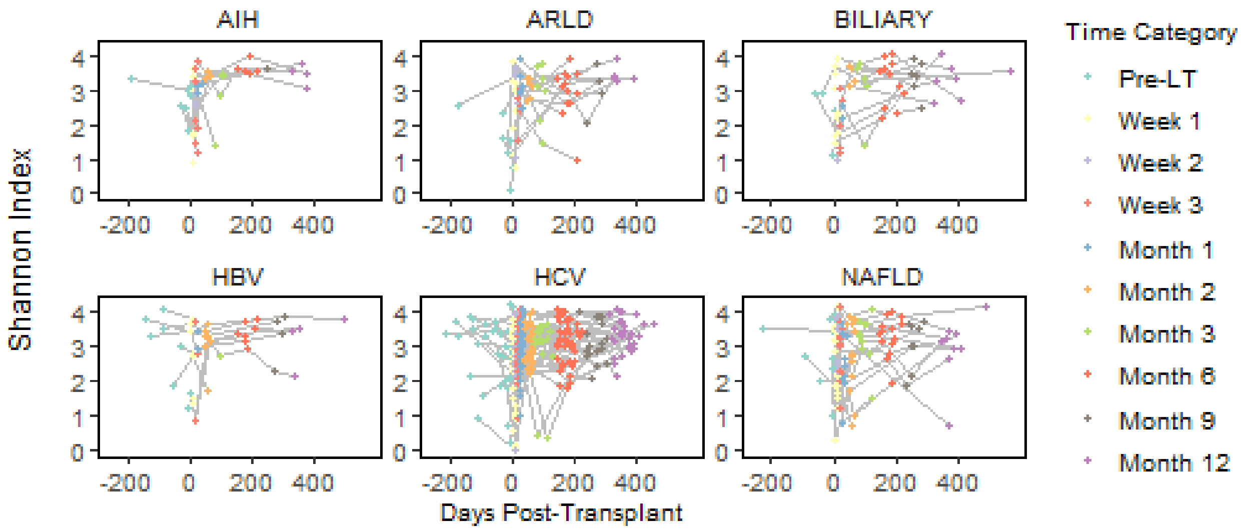

Panel B: Chao α-diversity over time

```
p3_b = ggplot(LT_data, mapping = aes(x=Days_Post_LT, y=Chao,
                        color=Time_Category, group=factor(StudyID))) +
  geom_line(color="grey") +
  geom_point(size = 1.25) +
  xlab("Days Post-Transplant") + ylab("Chao Index") +
  ggtitle("") +
  facet_wrap(~Primary_Diagnosis, ncol=3, scales = "free_x") +
  scale_color_manual(values=palette_a, labels=c("Pre-LT","Week 1","Week 2",
                                     "Week 3","Month 1","Month 2","Month 3",
                                     "Month 6","Month 9","Month 12")) +
  scale_x_continuous(limits=c(-250,570), breaks=c(-200,0,200,400)) +
  labs(color="Time Category") +
  theme(panel.grid = element_blank(), panel.background = element_blank(),
        panel.border = element_rect(fill=NA),
        axis.line = element_line(colour = "black"),
        strip.background = element_rect(fill=NA), strip.text = element_text(size=10),
        legend.title = element_text(size=10), legend.text= element_text(size=10),
        legend.key = element_blank(), plot.margin = margin(0,0,0,0),
        axis.text = element_text(size=10), axis.title.x=element_text(size=10),
        axis.title.y=element_text(size=12))

p3_b
```

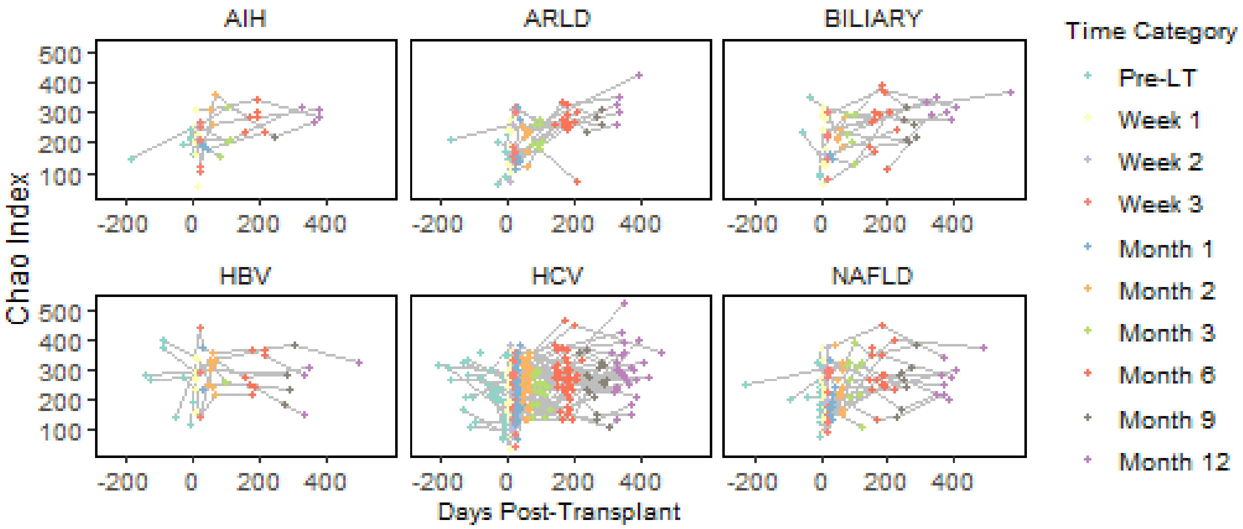

Constrained Linear Mixed-Effect (CLME) modeling of longitudinal α-diversity data

We used the `CLME` package in R to model the change in α-diversity over time for each of the six major diagnoses. Our aim was to determine whether α-diversity changed significantly over time and whether these changes differed across the diagnoses, as it appeared to in the panel A plot.

First we set the constraints; we used an umbrella model, with the index node as Week 1 in most cases (decreasing set to F, as this was the "valley" for α-diversity values). This was selected based on the plot for panel A, where Week 1 has the lowest Shannon and Chao diversity for almost all diagnoses

For ARLD only, the "valley" is at Pre-LT, so we set the index node as Pre-LT and keep all other parameters the same.

```
cons <- list(order = "umbrella", node=2, decreasing = FALSE)

cons_arld <- list(order = "umbrella", node=1, decreasing = FALSE)

# Note: umbrella order with node=1 and decreasing=F is effectively
# the same as simple order with increasing trend (identical outputs)
```

We then run CLME for each diagnosis group, looking at time category (Pre-LT and Week 1, 2, 3, Month 1, 2, 3, 6, 9, 12 post-LT) as the fixed effects (θ) and the Study ID as the random effect to account for repeated measures. The seed was set at 2 and we used 1000 bootstrap samples for the LRT to determine a global p-value for each model.

Shannon α-diversity

First, we run the model for each diagnosis with Shannon diversity as the outcome

AIH

```
clme.aih.shan <- clme(Shannon ~ Time_Category + (1|StudyID),
                  data = LT_data[LT_data$Primary_Diagnosis=="AIH",],
                  constraints = cons, seed = 2, nsim = 1000)
aih.shan <- summary(clme.aih.shan, seed=2)
```

ARLD

```
#Note the change in constraints

clme.arld.shan <- clme(Shannon ~ Time_Category + (1|StudyID),
                  data = LT_data[LT_data$Primary_Diagnosis=="ARLD",],
                  constraints = cons_arld, seed = 2, nsim = 1000)
arld.shan <- summary(clme.arld.shan, seed=2)
```

BILIARY

```
clme.bil.shan <- clme(Shannon ~ Time_Category + (1|StudyID),
                     data = LT_data[LT_data$Primary_Diagnosis=="BILIARY",],
                     constraints = cons, seed = 2, nsim = 1000)
bil.shan <- summary(clme.bil.shan, seed=2)
```

HBV

```
clme.hbv.shan <- clme(Shannon ~ Time_Category + (1|StudyID),
                 data = LT_data[LT_data$Primary_Diagnosis=="HBV",],
                 constraints = cons, seed = 2, nsim = 1000)
hbv.shan <- summary(clme.hbv.shan, seed=2)
```

HCV

```
clme.hcv.shan <- clme(Shannon ~ Time_Category + (1|StudyID),
                 data = LT_data[LT_data$Primary_Diagnosis=="HCV",],
                 constraints = cons, seed = 2, nsim = 1000)
hcv.shan <- summary(clme.hcv.shan, seed=2)
```

NAFLD

```
clme.nafld.shan <- clme(Shannon ~ Time_Category + (1|StudyID),
                   data = LT_data[LT_data$Primary_Diagnosis=="NAFLD",],
                   constraints = cons, seed = 2, nsim = 1000)
nafld.shan <- summary(clme.nafld.shan, seed=2)
```

Chao α-diversity

Then, we run the model for each diagnosis with Chao diversity as the outcome

AIH

```
clme.aih.chao <- clme(Chao ~ Time_Category + (1|StudyID),
                 data = LT_data[LT_data$Primary_Diagnosis=="AIH",],
                 constraints = cons, seed = 2, nsim = 1000)
aih.chao <- summary(clme.aih.chao, seed=2)
```

ARLD

```
clme.arld.chao <- clme(Chao ~ Time_Category + (1|StudyID),
                  data = LT_data[LT_data$Primary_Diagnosis=="ARLD",],
                  constraints = cons_arld, seed = 2, nsim = 1000)
arld.chao <- summary(clme.arld.chao, seed=2)
```

BILIARY

```
clme.bil.chao <- clme(Chao ~ Time_Category + (1|StudyID),
                      data = LT_data[LT_data$Primary_Diagnosis=="BILIARY",],
                      constraints = cons, seed = 2, nsim = 1000)
bil.chao <- summary(clme.bil.chao, seed=2)
```

HBV

```
clme.hbv.chao <- clme(Chao ~ Time_Category + (1|StudyID),
                  data = LT_data[LT_data$Primary_Diagnosis=="HBV",],
                  constraints = cons, seed = 2, nsim = 1000)
hbv.chao <- summary(clme.hbv.chao, seed=2)
```

HCV

```
clme.hcv.chao <- clme(Chao ~ Time_Category + (1|StudyID),
                  data = LT_data[LT_data$Primary_Diagnosis=="HCV",],
                  constraints = cons, seed = 2, nsim = 1000)
hcv.chao <- summary(clme.hcv.chao, seed=2)
```

NAFLD

```
clme.nafld.chao <- clme(Chao ~ Time_Category + (1|StudyID),
                    data = LT_data[LT_data$Primary_Diagnosis=="NAFLD",],
                    constraints = cons, seed = 2, nsim = 1000)
nafld.chao <- summary(clme.nafld.chao, seed=2)
```

Adding stats to Panels A and B

Finally, we add the resultant global p values to each facet in panel A and B

- Note: Thanks to Adam H. Sparks' script `add_p_r2_eqn.R` on Github and to Kamil Slowikowski on Stack Overflow for excellent inspirations/explanations on how to do the following!

```
clme.globalp <- function(model) {
  label <- substitute(
      italic(p) == globalp,
      list(globalp <- model$p.value)
    )
  as.character(as.expression(format(globalp, nsmall=3)))
}

aih.shan.globalp <- paste("CLME P=",clme.globalp(aih.shan))
arld.shan.globalp <- paste("CLME P=",clme.globalp(arld.shan))
bil.shan.globalp <- paste("CLME P=",clme.globalp(bil.shan))
hbv.shan.globalp <- paste("CLME P=",clme.globalp(hbv.shan))
hcv.shan.globalp <- paste("CLME P=",clme.globalp(hcv.shan))
nafld.shan.globalp <- paste("CLME P=",clme.globalp(nafld.shan))

aih.chao.globalp <- paste("CLME P=",clme.globalp(aih.chao))
arld.chao.globalp <- paste("CLME P=",clme.globalp(arld.chao))
bil.chao.globalp <- paste("CLME P=",clme.globalp(bil.chao))
hbv.chao.globalp <- paste("CLME P=",clme.globalp(hbv.chao))
hcv.chao.globalp <- paste("CLME P=",clme.globalp(hcv.chao))
nafld.chao.globalp <- paste("CLME P=",clme.globalp(nafld.chao))
```

```
annotation_panela = data.frame(label = c(aih.shan.globalp, arld.shan.globalp,
                                         bil.shan.globalp, hbv.shan.globalp,
                                         hcv.shan.globalp, nafld.shan.globalp),
                        Primary_Diagnosis = levels(LT_data$Primary_Diagnosis),
                        x = c(rep(550,6)),
                        y = c(rep(0.2,6)))

annotation_panelb = data.frame(label = c(aih.chao.globalp, arld.chao.globalp,
                                         bil.chao.globalp, hbv.chao.globalp,
                                         hcv.chao.globalp, nafld.chao.globalp),
                        Primary_Diagnosis = levels(LT_data$Primary_Diagnosis),
                        x = c(rep(550,6)),
                        y = c(rep(50,6)))

p3_a = p3_a + geom_text_repel(data = annotation_panela, inherit.aes = F,
                        mapping = aes(x = x, y = y, label = label),
                        hjust = 1, size=3, point.padding = NA,
                        direction = "x")

p3_b = p3_b + geom_text_repel(data = annotation_panelb, inherit.aes = F,
                        mapping = aes(x = x, y = y, label = label),
                        hjust = 1, size=3, point.padding = NA,
                        direction = "x")
```

Final panels A and B

```
p3_a
```

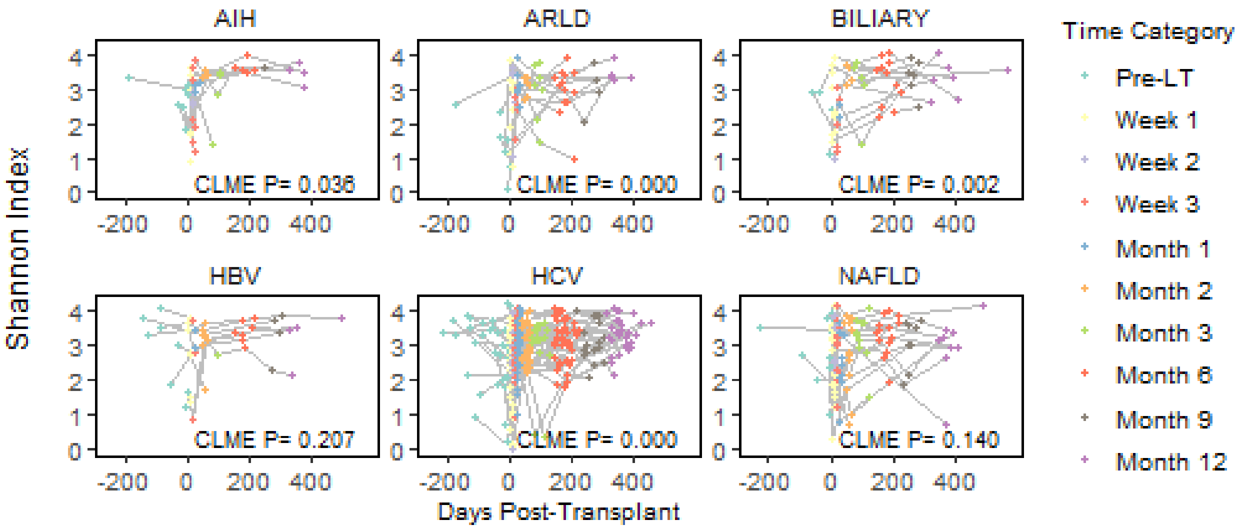


```
p3_b
```

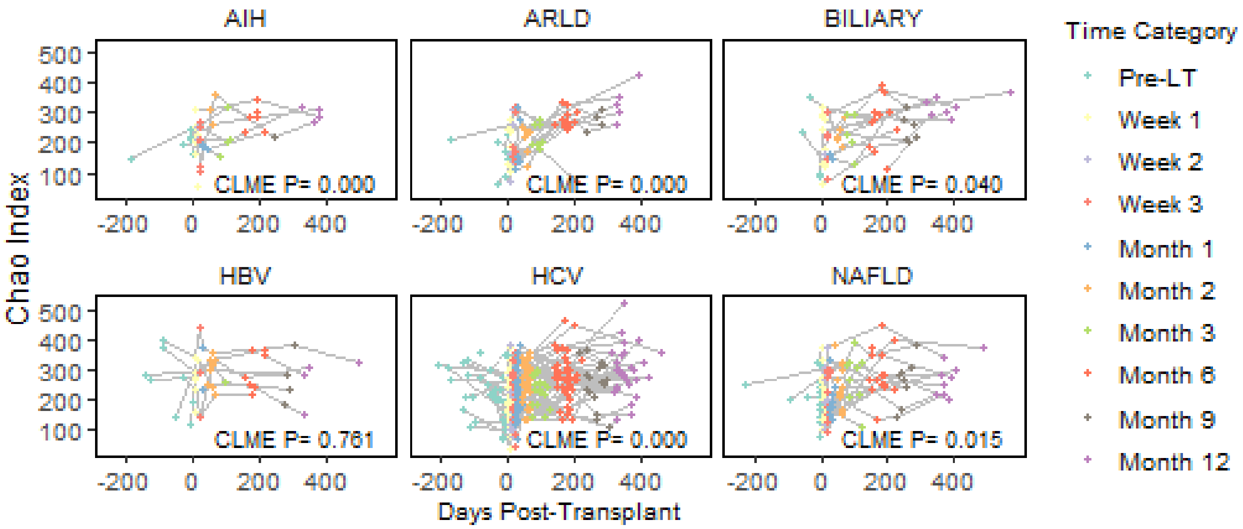

Generate Panel C (β-diversity across time periods)

Panel C shows the UniFrac β-diversity across time periods (Pre-LT, Peri-LT, Early Post-LT (Months 1-3), and Late Post-LT (Months 6-12)) for each diagnosis. We want to see if there is clustering of microbial communities by pre-/post-transplant phase. UniFrac distances were calculated for all samples first; the resulting ordination was faceted for plotting to easily view longitudinal progression within each diagnosis group. First, the data has to be imported in the phyloseq format, and our Figure 3 metadata can be merged in

```
phylo_relabun_filtered = readRDS("inputs/phylo_relabun_filtered.RDS")

LT_relabun = prune_samples(sample_names(phylo_relabun_filtered) %in%
                             LT_data$StoolID, phylo_relabun_filtered)

df1 <- LT_data[,1:6] #Metadata from data we used before
df2 <- data.frame(sample_data(LT_relabun),
                  "StoolID"=rownames(sample_data(LT_relabun))) #Sample names
merged <- merge(df2, df1, by="StoolID") #Merge metadata with phyloseq object
merged <- sample_data(merged)
sample_names(merged) = merged$StoolID #fix names
sample_data(LT_relabun) <- sample_data(merged)
```

Then, we calculate UniFrac distances, ordinate, and plot

```
palette_c = c("#f45342","#f7bd23","#1e8c69","#c19aff")

DistUF = phyloseq::distance(LT_relabun, "uniFrac")
ordUF = ordinate(LT_relabun, method = "PCoA", distance = DistUF)

p3_c = plot_ordination(LT_relabun, ordUF, color = "Time_Category2") +
  facet_wrap(~Primary_Diagnosis, ncol=3, scales="free_x") +
  labs(color="") +
  scale_color_manual(values=palette_c,
                     labels = c("Pre-LT","Peri-LT","Post-LT (M 1-3)", "Post-LT (M 6-12)")) +
  scale_x_continuous(limits=c(-0.4,0.4)) + theme_classic() +
  xlab("Axis 1 (14.5%)") +
```

```
  ylab("Axis 2 (5.6%)") +
  theme(panel.grid.major = element_blank(), panel.grid.minor = element_blank(),
        panel.background = element_blank(), panel.border = element_rect(fill=NA),
        axis.line = element_line(colour = "black"), strip.background = element_blank(),
        strip.text = element_text(size=10), legend.text = element_text(size=10),
        plot.margin = margin(2,0,0,0))

p3_c
```

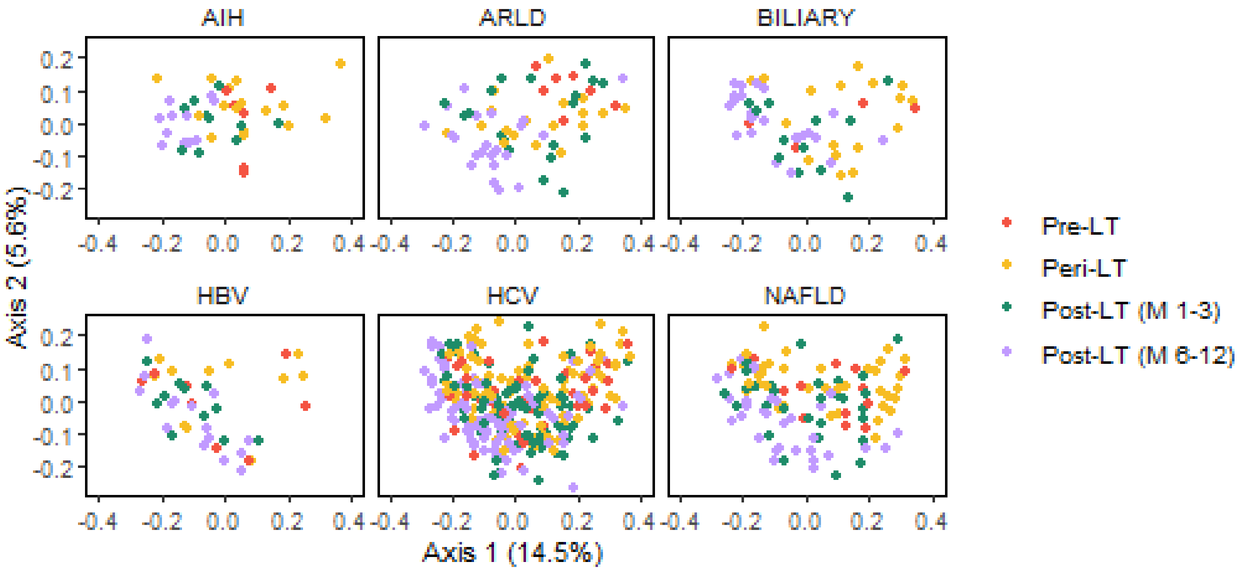

Putting it all together

```
egg::ggarrange(p3_a, p3_b, p3_c, ncol = 1,
          labels = c("A","B","C"),
          heights = c(1.15,1.15,1.25),
               label.args = list(gp=gpar(fontsize=18,
                                         fontfamily = "sans",
                                         fontface = "plain"),
                                 vjust=1.25, hjust=0))
```

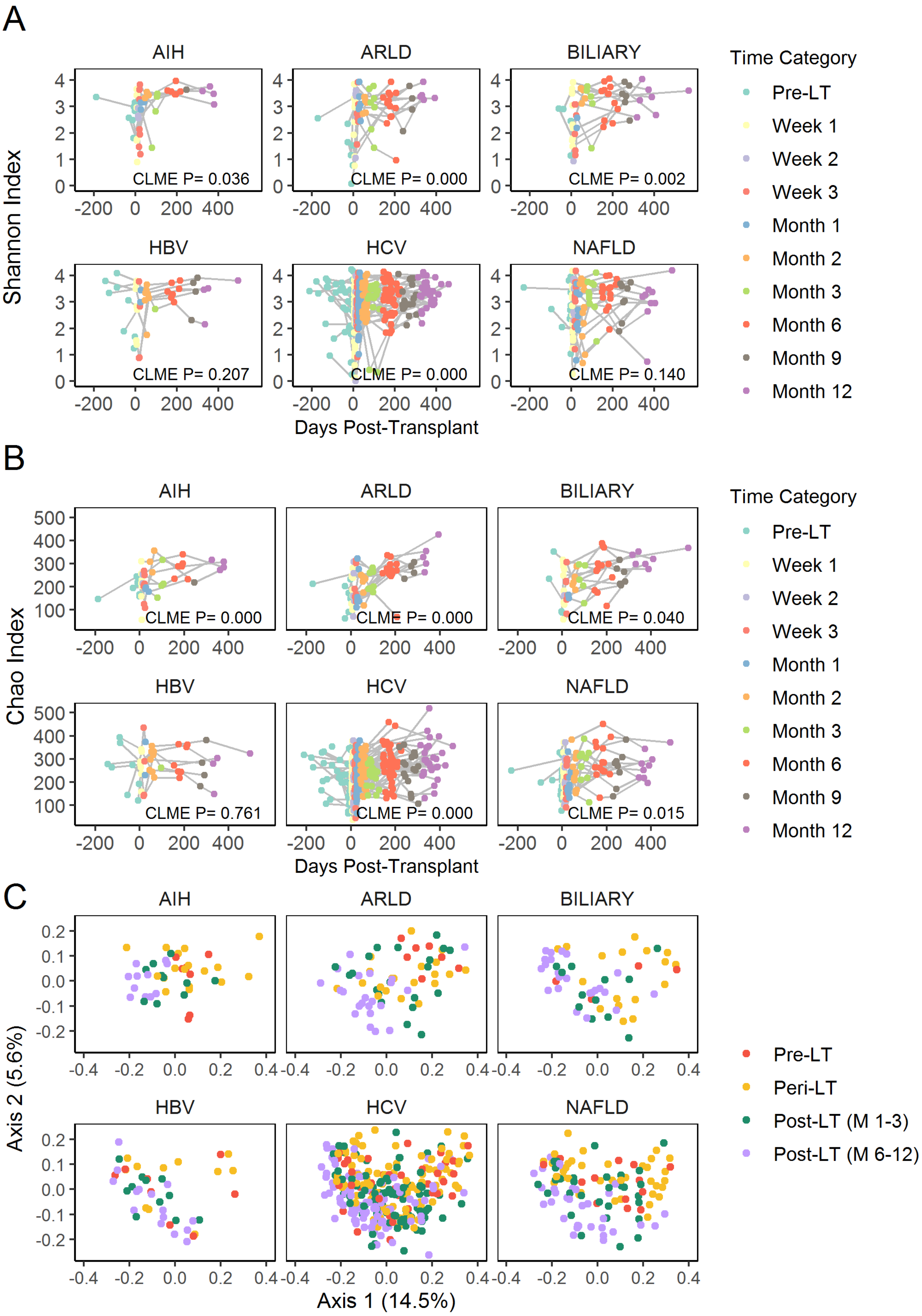
A
Shannon Index
AIH
CLME P= 0.036
ARLD
CLME P= 0.000
BILIARY
CLME P= 0.002
HBV
CLME P= 0.207
HCV
CLME P= 0.000
NAFLD
CLME P= 0.140
Days Post-Transplant
Time Category
Pre-LT
Week 1
Week 2
Week 3
Month 1
Month 2
Month 3
Month 6
Month 9
Month 12
B
Chao Index
AIH
CLME P= 0.000
ARLD
CLME P= 0.000
BILIARY
CLME P= 0.040
HBV
CLME P= 0.761
HCV
CLME P= 0.000
NAFLD
CLME P= 0.015
Days Post-Transplant
Time Category
Pre-LT
Week 1
Week 2
Week 3
Month 1
Month 2
Month 3
Month 6
Month 9
Month 12
C
Axis 2 (5.6%)
AIH
ARLD
BILIARY
HBV
HCV
NAFLD
Axis 1 (14.5%)
Pre-LT
Peri-LT
Post-LT (M 1-3)
Post-LT (M 6-12)
