# Supplementary material for: Colonizing multidrug-resistant bacteria and the longitudinal evolution of the intestinal microbiome after liver transplantation
Source: Nat Commun. 2019 Oct 17;10:4715. doi: 10.1038/s41467-019-12633-4 (PMC6797753; doi:10.1038/s41467-019-12633-4)
Supplement: Supplementary file 29 — Source Data [file 41467_2019_12633_MOESM29_ESM.zip › Source_Data/Figure4_Hypotheses.pdf]

Liver transplant microbiome and MDRO

Medini K. Annavajhala

July 19, 2019

Generating Figure 4: Overview of findings from multivariate models

This R Markdown document is part of a series used to analyze data and generate figures for the citation below. The current document includes all code and options used to generate the top panel of Figure 4 from the manuscript, which shows the average Shannon α–diversity for each of the six major underlying liver disease etiologies at each sampling period.

Title:

Colonizing multidrug-resistant organisms and the longitudinal evolution of the intestinal microbiome after liver transplantation

Authors:

Medini K. Annavajhala, Angela Gomez-Simmonds, Nenad Macesic, Sean B. Sullivan, Anna Kress, Sabrina D. Khan, Marla J. Giddins, Stephania Stump, Grace I. Kim, Ryan Narain, Elizabeth C. Verna, Anne-Catrin Uhlemann

Journal:

Nature Communications **2019**

Load Required Libraries:

```
library("data.table"); packageVersion("data.table")
```

```
## [1] '1.12.2'
```

```
library("ggplot2"); packageVersion("ggplot2")
```

```
## [1] '3.2.1'
```

```
library("dplyr"); packageVersion("dplyr")
```

```
## [1] '0.8.3'
```

Import and Format Data

We need to take Shannon diversity values for all samples from patients who had one of the 6 major underlying liver diseases (AIH, ARLD, BILIARY, HBV, HCV, NAFLD) and merge the α-diversity by both diagnosis and time category. This gives us an average Shannon index for each time category for each diagnosis, which we can then plot over time

```
alphadt_fig4 = read.table("inputs/Fig4_metadata.txt",sep="\t",header=T)

merged_alphadt_fig4 = aggregate(Shannon~Primary_Diagnosis+Time_Category,
                                alphadt_fig4, FUN=mean)
```

Plot and save Figure 4

```
palette = c("AIH"="#7FC97F","ARLD"="#F0027F","BILIARY"="#BEAED4",
            "HBV"="#FDC086","HCV"="#FFFF99","NAFLD"="#386CB0")

p4 = ggplot(merged_alphadt_fig4,mapping = aes(x=Time_Category, y=Shannon,
                                              color=Primary_Diagnosis,
                                              group=factor(Primary_Diagnosis))) +
  geom_line(mapping = aes(color=Primary_Diagnosis)) +
  geom_point(size = 1.5) +
  xlab("") + ylab("Shannon Index\n") +
  ggtitle("") +
  scale_color_manual(values=palette) +
  labs(color="Primary Underlying Etiology") +
  scale_x_discrete(labels= c("Pre-LT","W 1","W 2","W 3",
                             "M 1","M 2","M 3","M 6","M 9","M 12")) +
  theme(legend.key = element_rect(fill = "white", colour = "white"),
        panel.grid.major = element_blank(), panel.grid.minor = element_blank(),
        legend.background = element_blank(), panel.background = element_blank(),
        panel.border = element_rect(fill=NA), axis.line = element_line(colour = "black"),
        strip.background = element_rect(fill=NA), strip.text = element_text(size=12),
        legend.title = element_text(size=10), legend.text= element_text(size=8),
        axis.text = element_text(size=10), axis.title=element_text(size=12),
        legend.position = c(.95, .6), legend.justification = c("right", "top"),
        legend.box.just = "right", legend.margin = margin(1, 1, 1, 1)) +
  guides(color=guide_legend(ncol=2))

p4
```

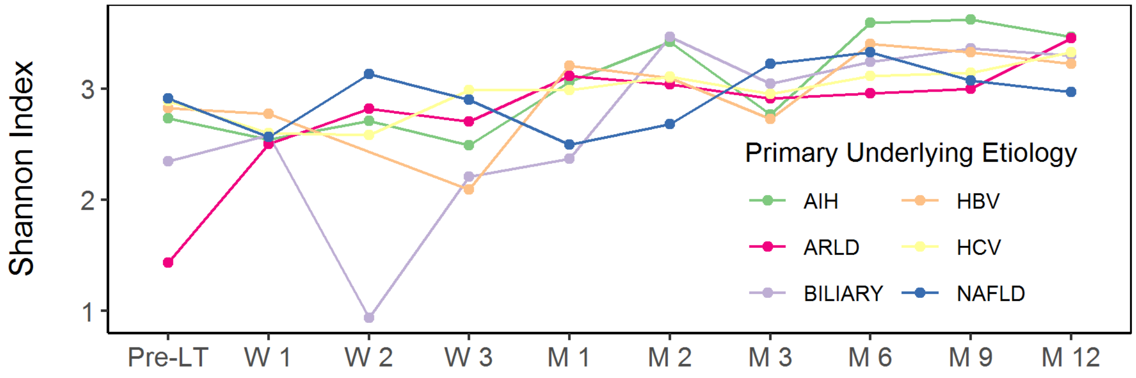

The additional components of Figure 4 were drawn and compiled by the author using Inkscape 0.92.2
