# Supplementary material for: Colonizing multidrug-resistant bacteria and the longitudinal evolution of the intestinal microbiome after liver transplantation
Source: Nat Commun. 2019 Oct 17;10:4715. doi: 10.1038/s41467-019-12633-4 (PMC6797753; doi:10.1038/s41467-019-12633-4)
Supplement: Supplementary file 29 — Source Data [file 41467_2019_12633_MOESM29_ESM.zip › Source_Data/Phyloseq_Objects.pdf]

Liver transplant microbiome and MDRO

Medini K. Annavajhala

July 17, 2019

Generating Phyloseq Objects and α-Diversity Metrics after QIIME1 OTU-calling

This R Markdown document is part of a series used to analyze data and generate figures for the citation below. The current document includes all code and options used to generate phyloseq objects from QIIME1-based OTU calling outputs and to calculate α-diversity metrics for further analysis.

Title:

Colonizing multidrug-resistant organisms and the longitudinal evolution of the intestinal microbiome after liver transplantation

Authors:

Medini K. Annavajhala, Angela Gomez-Simmonds, Nenad Macesic, Sean B. Sullivan, Anna Kress, Sabrina D. Khan, Marla J. Giddins, Stephania Stump, Grace I. Kim, Ryan Narain, Elizabeth C. Verna, Anne-Catrin Uhlemann

Journal:

Nature Communications **2019**

Author's Note: I learned so much from Paul J. McMurdie's site for all things `phyloseq` here; truly a fantastic resource for those getting started with microbiome data analysis

Load Required Libraries:

```
library("phyloseq"); packageVersion("phyloseq")
```

```
## [1] '1.28.0'
```

```
library("biomformat"); packageVersion("biomformat")
```

```
## [1] '1.12.0'
```

```
library("data.table"); packageVersion("data.table")
```

```
## [1] '1.12.2'
```

Import and parse input files

The biom file was generated using Greengenes 97 closed-reference OTU-calling through the NIAID Nephele platform, with default quality-filtering and paired-end-joining parameters

```
biom_file <- "inputs/merged.biom"
biomRead <- biomformat::read_biom(biom_file)
biom <- import_biom(biomRead, parseFunction = parse_taxonomy_greengenes)
rank_names(biom)
```

```
## [1] "Kingdom" "Phylum"  "Class"   "Order"   "Family"  "Genus"   "Species"
```

```
tax_table(biom) <- tax_table(biom)[, 1:7]
```

The tree is just the Greengenes 97 representative sequence tree as default, since we performed closed-reference OTU-calling (if we run open-reference OTU-calling, this would require a custom tree generated using the representative sequence from each called OTU)

```
tree <- read_tree_greengenes("inputs/tree.tre")
```

We had to re-sequence some samples due to low read counts the first time around. We gave them different names to avoid duplicate records in the output biom file, which would throw an error. Here, the original (low-read-count) sample IDs are removed. To do this, we create a phyloseq object with artificial metadata (only the sample names), remove the sample names we don't want, and then substring the remaining sample IDs to the original 5-digit numeric code that we use in our lab

```
fake_metadata = data.frame(sample_names(biom)) #get just sample names as 'metadata'
fake_metadata = sample_data(fake_metadata) #convert to phyloseq sample_data type
sample_names(fake_metadata) = fake_metadata$sample_names.biom

dups <- unlist(read.table("inputs/dups.txt")) #list of duplicated sample IDs

phylo_init <- merge_phyloseq(biom,tree,fake_metadata) #generate artificial phyloseq object
phylo = prune_samples(!(sample_data(phylo_init)$sample_names.biom %in% dups), phylo_init)

sample_names(phylo) <- substr(sample_names(phylo),1,5) #substring names to 5-digit codes
phylo #We finally ended up with 703 samples which passed all QC
```

```
## phyloseq-class experiment-level object
## otu_table()   OTU Table:         [ 1478 taxa and 703 samples ]
## sample_data() Sample Data:       [ 703 samples by 1 sample variables ]
## tax_table()   Taxonomy Table:    [ 1478 taxa by 7 taxonomic ranks ]
## phy_tree()    Phylogenetic Tree: [ 1478 tips and 1477 internal nodes ]
```

I like to make a new taxonomy column in the phyloseq object's `tax_table` titled Genus_Species for easier labeling of figures, etc. down the line

```
tax = as.data.frame(tax_table(phylo))
tax$Genus_Species = paste(tax$Genus,tax$Species,sep=" ")
tax$Genus_Species = gsub(" NA","", tax$Genus_Species) #remove "NA" for OTUs with
#no species-level classification
taxtable = tax_table(as.matrix(tax))
rownames(taxtable) = taxa_names(phylo)
colnames(taxtable) = c("Kingdom","Phylum","Class","Order","Family",
                       "Genus","Species","Genus_Species")
tax_table(phylo) = taxtable
```

Generate and Filter Final Phyloseq Objects

Now we convert from counts to relative abundance. Some downstream analyses are based on raw counts (i.e. α-diversity, differential abundance testing) and some on relative abundance (i.e. β-diversity)

```
phylo_relabun <- transform_sample_counts(phylo, function(x) x / sum(x))
```

However, before we begin, we have to filter out extremely low-abundance OTUs; these are likely artefacts from sequencing and add noise to the analyses. There are no standardized, published cutoffs for this - we use a very conservative cutoff of 0.005% average relative abundance across all samples in order to prevent over-filtering of the data

```
phylo_filter = filter_taxa(phylo_relabun, function(x) mean(x) < .00005,TRUE)
rmtaxa = taxa_names(phylo_filter) #these are the taxa that will be removed
alltaxa = taxa_names(phylo_relabun)
```

```r
myTaxa = alltaxa[!alltaxa %in% rmtaxa]

#Filter both count- and relative abundance-based phyloseq objects

phylo_relabun_filtered <- prune_taxa(myTaxa,phylo_relabun)
phylo_relabun_filtered
```

```
## phyloseq-class experiment-level object
## otu_table()   OTU Table:         [ 878 taxa and 703 samples ]
## sample_data() Sample Data:       [ 703 samples by 1 sample variables ]
## tax_table()   Taxonomy Table:    [ 878 taxa by 8 taxonomic ranks ]
## phy_tree()    Phylogenetic Tree: [ 878 tips and 877 internal nodes ]
```

```r
phylo_filtered <- prune_taxa(myTaxa,phylo)
phylo_filtered
```

```
## phyloseq-class experiment-level object
## otu_table()   OTU Table:         [ 878 taxa and 703 samples ]
## sample_data() Sample Data:       [ 703 samples by 1 sample variables ]
## tax_table()   Taxonomy Table:    [ 878 taxa by 8 taxonomic ranks ]
## phy_tree()    Phylogenetic Tree: [ 878 tips and 877 internal nodes ]
```

We go from 1478 taxa in phylo and phylo_relabun to 878 taxa in phylo_filtered and phylo_relabun_filtered

This is a good time to save the filtered phyloseq objects for future reference!

```r
saveRDS(phylo_relabun_filtered, "inputs/phylo_relabun_filtered.RDS")
saveRDS(phylo_filtered, "inputs/phylo_filtered.RDS")
```

Lastly, we can use the `plot_richness` function to calculate α-diversity (Shannon, Chao) for each sample and then save the values for subsequent plotting and statistical analyses

```r
pAlpha = plot_richness(phylo_filtered, measures = c("Chao1", "Shannon"))
alphadt = data.table(pAlpha$data)
write.table(alphadt, "inputs/LT_alpha_div.txt", row.names=F, sep="\t")
```
