# Supplementary material for: Colonizing multidrug-resistant bacteria and the longitudinal evolution of the intestinal microbiome after liver transplantation
Source: Nat Commun. 2019 Oct 17;10:4715. doi: 10.1038/s41467-019-12633-4 (PMC6797753; doi:10.1038/s41467-019-12633-4)
Supplement: Supplementary file 29 — Source Data [file 41467_2019_12633_MOESM29_ESM.zip › Source_Data/Supp_Figure2_Key_Taxa_Abundance.pdf]

Liver transplant microbiome and MDRO

Medini K. Annavajhala

July 24, 2019

Generating Supplementary Figure 2: Relative abundance of key taxa in patients who did vs. did not develop MDRO up to 1-year post-LT

This R Markdown document is part of a series used to analyze data and generate figures for the citation below. The current document includes the code used to generate Supplementary Figure 2 from the manuscript, which shows the average relative abundance of 12 key taxa throughout the pre- to 1-year post-LT time period, stratified by patients who did vs. did not develop MDRO (at any point during the study period).

Title:

Colonizing multidrug-resistant organisms and the longitudinal evolution of the intestinal microbiome after liver transplantation

Authors:

Medini K. Annavajhala, Angela Gomez-Simmonds, Nenad Macesic, Sean B. Sullivan, Anna Kress, Sabrina D. Khan, Marla J. Giddins, Stephania Stump, Grace I. Kim, Ryan Narain, Elizabeth C. Verna, Anne-Catrin Uhlemann

Journal:

Nature Communications **2019**

Load Required Libraries and Functions:

```
library("phyloseq"); packageVersion("phyloseq")
```

```
## [1] '1.28.0'
```

```
library("ggplot2"); packageVersion("ggplot2")
```

```
## [1] '3.2.1'
```

```
library("gridExtra"); packageVersion("gridExtra")
```

```
## [1] '2.3'
```

```
merge_samples_mean <- function(physeq, group){

  group_sums <- as.matrix(table(sample_data(physeq)[ ,group]))[,1]

  merged <- merge_samples(physeq, group)

  x <- as.matrix(otu_table(merged))
  if(taxa_are_rows(merged)){ x<-t(x) }
  out <- t(x/group_sums)

  out <- otu_table(out, taxa_are_rows = TRUE)
  otu_table(merged) <- out
```

```
  return(merged)
}
```

Load data:

```
LT_data <- read.table("inputs/Supp_Fig2_metadata.txt", header=T)
colnames(LT_data)
```

```
## [1] "StoolID"        "Time_Category"  "MDRO_within1yr" "TimeCat_MDRO"
```

```
# Time_Category: One of 10 pre-selected sampling timepoints; pre-LT, then
#                  Weeks 1, 2, 3 and Months 1, 2, 3, 6, 9, and 12 post-LT
#
# MDRO_within1yr: binary (0=no; 1=yes) variable indicating whether the patient associated
#                 with the sample was culture-positive for CRE, VRE, and/or Ceph-RE at any
#                 point up to 1-yr post-LT
#
# TimeCat_MDRO: Concatenation of first two columns (this won't be used directly,
#                but makes merging relative abundance data easier


# Read in phyloseq object (see Phyloseq_Objects.Rmd)
LT_relabun = readRDS("inputs/phylo_relabun_filtered.RDS")

df <- data.frame(sample_data(LT_relabun),
                 "StoolID"=rownames(sample_data(LT_relabun))) #Sample names
merged <- merge(df, LT_data, by="StoolID") #Merge metadata with phyloseq object
merged <- sample_data(merged)
sample_names(merged) = merged$StoolID #fix names
sample_data(LT_relabun) <- sample_data(merged)
```

Merge and clean data

```
LT_relabun_MDRO = merge_samples_mean(LT_relabun, "TimeCat_MDRO")
            # Merge by timepoint + MDRO within 1 yr
sample_data(LT_relabun_MDRO)$TimeCat_MDRO = factor(sample_names(LT_relabun_MDRO))
LT_relabun_MDRO_genus = tax_glom(LT_relabun_MDRO, "Genus")
            # Merge relative abundance at genus level
otu_tax = cbind(otu_table(LT_relabun_MDRO_genus), tax_table(LT_relabun_MDRO_genus))
totu = t(otu_tax)
colnames(totu)=totu["Genus",]
df = data.frame(totu)
df$samples = factor(rownames(df))
df = df[1:20,] # First 20 rows (10 time points x 2 levels of MDRO (yes/no))
df$timepoints = c(rep("Pre-LT",2),rep("Week 1",2),rep("Week 2",2),rep("Week 3",2),
                  rep("Month 1",2),rep("Month 2",2),rep("Month 3",2),rep("Month 6",2),
                  rep("Month 9",2),rep("Month 12",2))
df$timepoints = factor(df$timepoints, levels=c("Pre-LT","Week 1","Week 2","Week 3",
                                               "Month 1","Month 2","Month 3","Month 6",
                                               "Month 9","Month 12"))
df$mdro = rep(c("FALSE","TRUE"),10)

df[1:5,1:5]
```

```
##                    Campylobacter           Comamonas             Delftia
## a-Pre_0   7.77966046943941e-05                    0                   0
## a-Pre_1   0.000926560161990164  3.2381825765053e-07                   0
## b-Week1_0  0.00053930230366015                    0 2.06576571737846e-06
## b-Week1_1 0.000245764051084476 0.000491202137638932  3.2100667693888e-07
## c-Week2_0   6.794148445916e-06                    0                   0
##                     Sutterella          Pseudomonas
## a-Pre_0   0.00112898426267897                    0
## a-Pre_1   0.00165152102367257 1.40801700003621e-06
## b-Week1_0  0.0010901020827251 2.06576571737846e-06
## b-Week1_1   0.001031829861426  0.00110862902327066
## c-Week2_0  0.0082379558706806                    0
```

Plot relative abundance over time

Next, we plot relative abundance of 12 selected taxa over time, stratified by development of MDRO within 1-year of transplant. These were primarily selected from differential abundance results using results from `DESeq2` and `ANCOM` analyses (see Differential_Abundance.Rmd)

```r
# Convert relative abundance to numeric data for our 12 taxa

df$Bacteroides = as.numeric(as.character(df$Bacteroides))
df$Bifidobacterium = as.numeric(as.character(df$Bifidobacterium))
df$Lachnospira = as.numeric(as.character(df$Lachnospira))
df$Enterobacter= as.numeric(as.character(df$Enterobacter))
df$Enterococcus = as.numeric(as.character(df$Enterococcus))
df$Faecalibacterium = as.numeric(as.character(df$Faecalibacterium))
df$Klebsiella = as.numeric(as.character(df$Klebsiella))
df$Lactobacillus = as.numeric(as.character(df$Lactobacillus))
df$Prevotella = as.numeric(as.character(df$Prevotella))
df$X.Ruminococcus. = as.numeric(as.character(df$X.Ruminococcus.))
df$Streptococcus = as.numeric(as.character(df$Streptococcus))
df$Veillonella = as.numeric(as.character(df$Veillonella))

palpha_1yr_bac = ggplot(df, mapping = aes(x=timepoints, y=Bacteroides*100,
                                    shape=mdro, group=factor(mdro))) +
                                    geom_line(color="blue") +
                                    geom_point(color="blue",size = 3) +
                                    guides(shape=F) + xlab("") + ggtitle("") +
                                    theme_classic() + ylab("") +
                                    theme(axis.text.x = element_blank(),
                                          axis.text.y=element_text(size=12))

palpha_1yr_bif = ggplot(df, mapping = aes(x=timepoints, y=Bifidobacterium*100,
                                    shape=mdro, group=factor(mdro))) +
                                    geom_line(color="purple") +
                                    geom_point(color="purple",size = 3) +
                                    guides(shape=F) + xlab("") +  ggtitle("") +
                                    theme_classic() + ylab("") +
                                    theme(axis.text.x = element_blank(),
                                          axis.text.y=element_text(size=12))

palpha_1yr_lachno = ggplot(df, mapping = aes(x=timepoints, y=Lachnospira*100,
                                    shape=mdro, group=factor(mdro))) +
                                    geom_line(color="green") +
```

```
                                    geom_point(color="green",size = 3) +
                                    guides(shape=F) + xlab("") + ggtitle("") +
                                    theme_classic() + ylab("") +
                                    theme(axis.text.x = element_blank(),
                                          axis.text.y=element_text(size=12))

palpha_1yr_entb = ggplot(df, mapping = aes(x=timepoints, y=Enterobacter*100,
                                    shape=mdro, group=factor(mdro))) +
                                    geom_line(color="red") +
                                    geom_point(color="red",size = 3) +
                                    guides(shape=F) + xlab("") + ggtitle("") +
                                    theme_classic() + ylab("") +
                                    theme(axis.text.x = element_blank(),
                                          axis.text.y=element_text(size=12))

palpha_1yr_entc = ggplot(df, mapping = aes(x=timepoints, y=Enterococcus*100,
                                    shape=mdro, group=factor(mdro))) +
                                    geom_line(color="grey") +
                                    geom_point(color="grey",size = 3) +
                                    guides(shape=F) + xlab("") + ggtitle("") +
                                    theme_classic() + ylab("") +
                                    theme(axis.text.x = element_blank(),
                                          axis.text.y=element_text(size=12))

palpha_1yr_faec = ggplot(df, mapping = aes(x=timepoints, y=Faecalibacterium*100,
                                    shape=mdro, group=factor(mdro))) +
                                    geom_line(color="orange") +
                                    geom_point(color="orange",size = 3) +
                                    guides(shape=F) + xlab("") +  ggtitle("") +
                                    theme_classic() + ylab("") +
                                    theme(axis.text.x = element_blank(),
                                          axis.text.y=element_text(size=12))

palpha_1yr_kleb = ggplot(df, mapping = aes(x=timepoints, y=Klebsiella*100,
                                    shape=mdro, group=factor(mdro))) +
                                    geom_line(color="#ffcc00") +
                                    geom_point(color="#ffcc00",size = 3) +
                                    guides(shape=F) + xlab("") + ggtitle("") +
                                    theme_classic() + ylab("") +
                                    theme(axis.text.x = element_blank(),
                                          axis.text.y=element_text(size=12))

palpha_1yr_lacto = ggplot(df, mapping = aes(x=timepoints, y=Lactobacillus*100,
                                    shape=mdro, group=factor(mdro))) +
                                    geom_line(color="pink") +
                                    geom_point(color="pink",size = 3) +
                                    guides(shape=F) + xlab("") +  ggtitle("") +
                                    theme_classic() + ylab("") +
                                    theme(axis.text.x = element_blank(),
                                          axis.text.y=element_text(size=12))

palpha_1yr_prev = ggplot(df, mapping = aes(x=timepoints, y=Prevotella*100,
                                    shape=mdro, group=factor(mdro))) +
```

```
                                    geom_line(color="black") +
                                    geom_point(color="black",size = 3) +
                                    guides(shape=F) + xlab("") + ggtitle("") +
                                    theme_classic()+ ylab("") +
                                    theme(axis.text.x = element_blank(),
                                          axis.text.y=element_text(size=12))

palpha_1yr_rumino = ggplot(df, mapping = aes(x=timepoints, y=X.Ruminococcus.*100,
                                    shape=mdro, group=factor(mdro))) +
                                    geom_line(color="mediumorchid1") +
                                    geom_point(color="mediumorchid1",size = 3) +
                                    guides(shape=F) + xlab("") +  ggtitle("") +
                                    theme_classic() + ylab("") +
                                    theme(axis.text.x = element_blank(),
                                          axis.text.y=element_text(size=12))

palpha_1yr_strep = ggplot(df, mapping = aes(x=timepoints, y=Streptococcus*100,
                                    shape=mdro, group=factor(mdro))) +
                                    geom_line(color="brown") +
                                    geom_point(color="brown",size = 3) +
                                    guides(shape=F) + xlab("") +  ggtitle("") +
                                    theme_classic() + ylab("") +
                                    theme(axis.text.x = element_blank(),
                                          axis.text.y=element_text(size=12))

palpha_1yr_veill = ggplot(df, mapping = aes(x=timepoints, y=Veillonella*100,
                                    shape=mdro, group=factor(mdro))) +
                                    geom_line(color="burlywood1") +
                                    geom_point(color="burlywood1",size = 3) +
                                    guides(shape=F) + xlab("") +  ggtitle("") +
                                    theme_classic() + ylab("") +
                                    theme(axis.text.x = element_blank(),
                                          axis.text.y=element_text(size=12))
```

Putting it all together

```
grid.arrange(palpha_1yr_bac, palpha_1yr_bif, palpha_1yr_lachno, palpha_1yr_entb,
             palpha_1yr_entc, palpha_1yr_faec, palpha_1yr_kleb, palpha_1yr_lacto,
             palpha_1yr_prev, palpha_1yr_rumino, palpha_1yr_strep, palpha_1yr_veill,
             ncol=3)
```

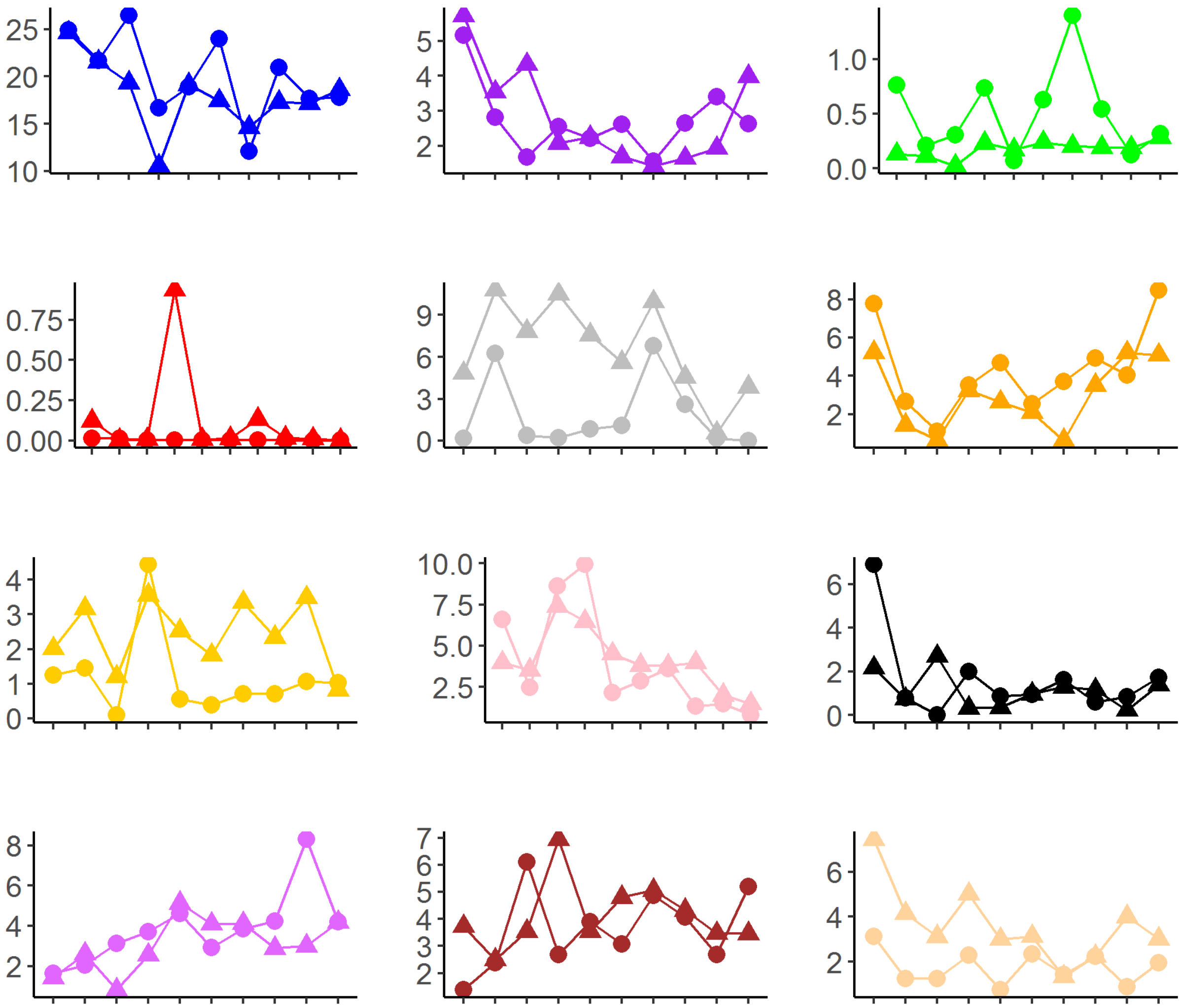

Note: Cosmetic additions (better-formatted legend, axis titles, panel titles) added in Inkscape.
