# Supplementary figures and images for: Colonizing multidrug-resistant bacteria and the longitudinal evolution of the intestinal microbiome after liver transplantation
Source: Nat Commun. 2019 Oct 17;10:4715. doi: 10.1038/s41467-019-12633-4 (PMC6797753; doi:10.1038/s41467-019-12633-4)

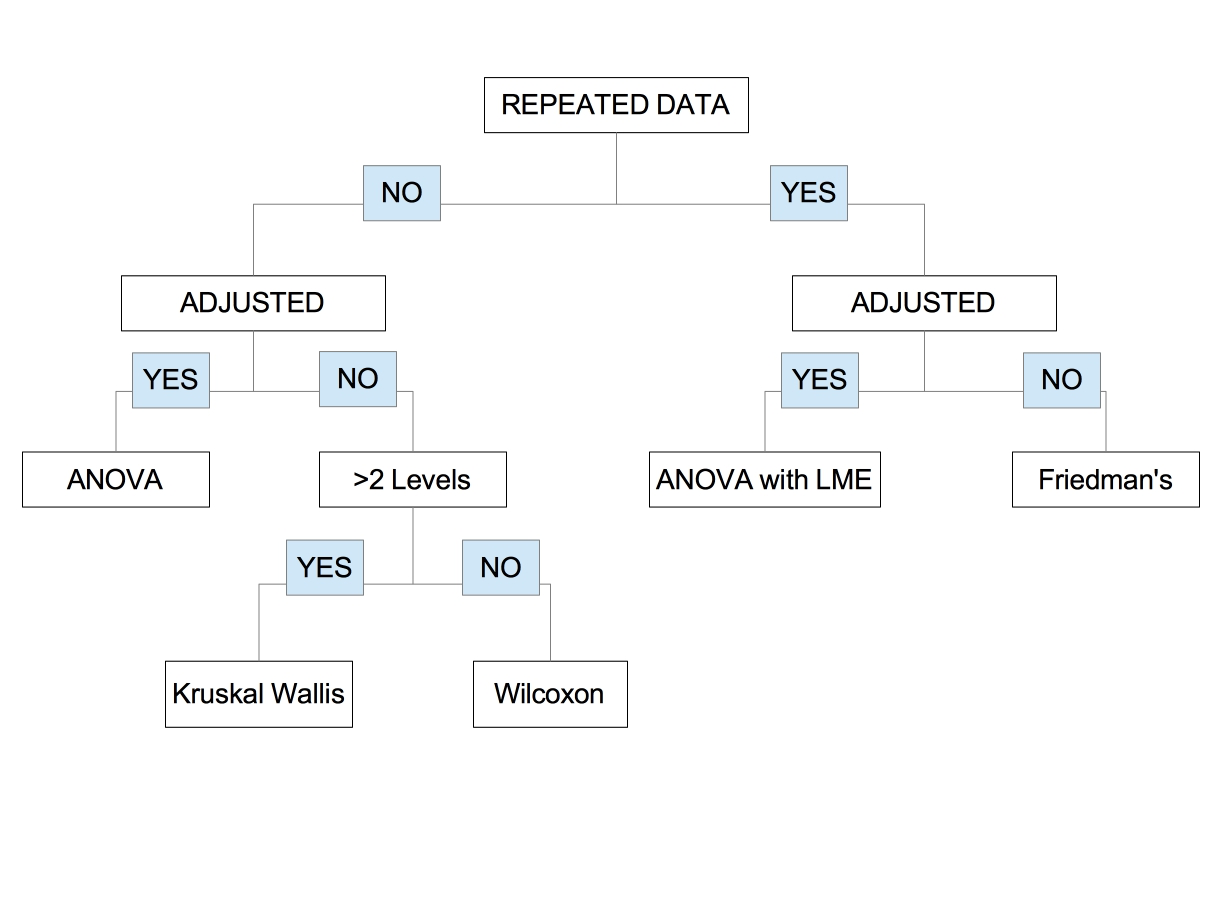

Supplement: Supplementary file 29 — Source Data [file 41467_2019_12633_MOESM29_ESM.zip › Source_Data/inputs/ancom_flowchart.png]
